# Supplementary material for: Learning low-rank latent mesoscale structures in networks
Source: Nat Commun. 2024 Jan 3;15:224. doi: 10.1038/s41467-023-42859-2 (PMC10764844; doi:10.1038/s41467-023-42859-2)
Supplement: Supplementary file 1 — Supplementary Information [file 41467_2023_42859_MOESM1_ESM.pdf]

Supplementary Information:

LEARNING LOW-RANK LATENT MESOSCALE STRUCTURES IN NETWORKS

In this supplement, we present our algorithms for network dictionary learning (NDL) and network denoising and reconstruction (NDR), and we prove theoretical results about their convergence and error bounds. In Appendix A, we define a variety of technical terms and overview our theoretical results. In Supplementary Table 1 in Appendix A.5, we highlight key differences between the present paper and Ref. [13]. In Appendix B, we discuss Markov-chain Monte Carlo (MCMC) motif-sampling algorithms. We give the complete NDL algorithm (see Algorithm NDL) in Appendix C. We introduce the notion of 'latent-motif dominance' in Appendix C.2 to measure the significance of each latent motif that we learn from a network. In Appendix C.4, we show that various mesoscale structures of the networks we study in the present paper emerge in the latent motifs at various scales $k \in \{6, 11, 21, 51\}$. In Appendix D, we give the complete NDR algorithm (see Algorithm NDR). We give experimental details in Appendix E. In Appendix F, we present a rigorous analysis of the NDL and NDR algorithms. In Appendix G, we state auxiliary algorithms that we use in the present paper. In Appendix H, we show additional figures.

Appendix A. Problem formulation and overview of theoretical results

A.1. **Definitions and notation.** To facilitate our discussions, we use terminology and notation from [55, Ch. 3]. We represent a network as a graph $G = (V, E)$ with a node set V and an edge set E without directed or multi-edges, but possibly with self-edges. An unordered pair $\{x, y\}$ of nodes of G is an *edge* of G if $\{x, y\} \in E$; it is a *self-edge* at x if $\{x\} \in E$. One can characterize the edge set E of $\mathcal{G}$ using an *adjacency matrix* $A_G : V^2 \to \{0, 1\}$, where $A(x, y) = \mathbb{1}(\{x, y\} \in E)$ for each $x, y \in V$. The function $\mathbb{1}(S)$ denotes the indicator of the event S; it takes the value 1 if S occurs and takes the value 0 if S does not occur. In this supplement, we formulate our NDL framework in the more general setting in which the edges of a network can have weights. Although one can extend the above definition of networks to include weighted edges by adjoining an additional object to $G = (V, E)$ for edge weights, it is convenient to instead extend the range of adjacency matrices from $\{0, 1\}$ to the interval $[0, \infty)$.

We define a *network* as a pair $\mathcal{G} = (V, A_\mathcal{G})$ with a node set V and a *weight matrix* (which is also often called a 'weighted adjacency matrix') $A_\mathcal{G} : V^2 \to [0, \infty)$ that encodes the weights of the edges between nodes. For simplicity, we often drop the subscript $\mathcal{G}$ in $A_\mathcal{G}$ and denote it by A. A graph $G = (V, E)$ determines a unique network $\mathcal{G} = (V, A_G)$, where A_G is the adjacency matrix of G. The set $V(\mathcal{G})$ is the node set of the network $\mathcal{G}$, which has *size* $|V(\mathcal{G})|$, where $|S|$ is the number of elements in the set S. An unordered pair $\{x, y\}$ of nodes of $\mathcal{G}$ is an *edge* if $A(x, y) > 0$ or $A(y, x) > 0$; it is a *nonedge if* $A(x, y) = 0$*;* it is a *self-edge* if $x = y$ and $A(x, x) > 0$. An ordered pair (x, y) of nodes of $\mathcal{G}$ is called a *directed edge* if $A(x, y) > 0$.

We say that a network $\mathcal{G} = (V, A)$ is *symmetric* if its weight matrix is symmetric (i.e., $A(x, y) = A(y, x)$ for all $x, y \in V$), and we say that it is *binary* (i.e., unweighted) if $A(x, y) \in \{0, 1\}$ for all $x, y \in V$. The network $\mathcal{G}$ is *bipartite* if it admits a 'bipartition', which is a partition $V = V_1 \cup V_2$ of the node set V such that $V = V_1 \cup V_2$ and $A(x, y) = 0$ if $x, y \in V_1$ or $x, y \in V_2$ for each $x, y \in V$. If two networks $\mathcal{G} = (V, A)$ and $\mathcal{G}' = (V', A')$ satisfy $V' \subseteq V$ and $A'(x, y) \le A(x, y)$ for all $x, y \in V'$, then we say that $\mathcal{G}'$ is a *subgraph* of $\mathcal{G}$ and write $\mathcal{G}' \subseteq \mathcal{G}$. If $A'(x, y) = A(x, y)$

for all $x, y \in V'$, then we say that $\mathcal{G}'$ is an *induced subgraph of* $\mathcal{G}$ that is induced by the node set V'.

For an integer $k \geq 2$ and nodes $x, y \in V$, we refer to a sequence $(x_1, \ldots, x_k)$ of (not necessarily distinct) nodes of $\mathcal{G}$ as a *k-walk from x to y* if $A(x_i, x_{i+1}) > 0$ for all $i \in \{1, \ldots, k-1\}$ and $(x_1, x_k) = (x, y)$. A k-walk $(x_1, \ldots, x_k)$ is a *k-path* if all nodes $x_1, \ldots, x_k$ are distinct. We say that a network $\mathcal{G}$ is *connected* if for any nodes $x, y \in V$, there exists a k-path from x to y for some $k \geq 1$. If $\mathcal{G}$ is connected, then for any two distinct nodes $x, y \in V$, we define $d_{\mathcal{G}}(x, y)$ to be the smallest integer $k \geq 1$ such that there exists a k-walk from x to y. The quantity $d_{\mathcal{G}}(x, y)$ is called the *shortest-path distance* between x and y. The maximum of $d_{\mathcal{G}}(x, y)$ over all node pairs (x, y) is the *shortest-path diameter* $\mathrm{diam}(\mathcal{G})$ of $\mathcal{G}$. It equals the minimum number of edges in a walk on $\mathcal{G}$ that connects nodes x and y.

Suppose that we are given N elements $\mathbf{v}_1, \ldots, \mathbf{v}_m$ in some vector space. When we say that we take their *mean*, we refer to their sample mean $\bar{\mathbf{v}} = N^{-1} \sum_{i=1}^{N} \mathbf{v}_i$. When we say that we take a *weighted average* of them, we refer to the expectation $\sum_{i=1}^{N} \mathbf{v}_i p_i$, where $(p_1, \ldots, p_N)$ is a probability distribution on the set of N elements.

A.2. **Homomorphisms between networks and motif sampling.** The ability to sample from a complex data set according to a known probability distribution (e.g., a uniform distribution) is a crucial ingredient in dictionary-learning problems. For instance, in image-processing applications [22, 23, 53], it is straightforward to uniformly randomly sample a $k \times k$ patch from an image. However, it is not straightforward to uniformly randomly sample a connected k-node subgraph of a network [56–59]. To develop dictionary learning for networks, we use motif sampling, which was introduced recently in [21]. In motif sampling, instead of directly sampling a connected subgraph, one samples a random function that maps the node set of a smaller network (i.e., a motif) to the node set of a target network while preserving adjacency relationships. One then uses the subgraph that is induced by the nodes in the image of the function. As we discuss below, such a function between networks is a homomorphism.

Fix an integer $k \geq 1$ and a weight matrix $A_F : [k]^2 \to [0, \infty)$, where we use the shorthand notation $[k] = \{1, \ldots, k\}$. We use the term *motif* for the corresponding network $F = ([k], A_F)$. A motif is a network, and we use motifs to sample from a given (and much larger) network. The type of motif that particularly interests us is a *k-chain*, for which $A_F = \mathbb{1}(\{(1,2), (2,3), \ldots, (k-1, k)\})$. A k-chain is a directed path with node set $[k]$. For simplicity, we refer to the k-chain motif with the corresponding network $F = ([k], A_F)$ as the *k-chain motif* $F = ([k], A_F)$. For a general k-node motif $F = ([k], A_F)$ and a network $\mathcal{G} = (V, A)$, we define the probability distribution $\pi_{F \to \mathcal{G}}$ on the set $V^{[k]}$ of all node maps $\mathbf{x} : [k] \to V$ (i.e., functions between node sets) by

$$\pi_{F \to \mathcal{G}}(\mathbf{x}) := \frac{1}{\mathtt{Z}} \left(\prod_{i,j \in \{1, \ldots, k\}} A(\mathbf{x}(i), \mathbf{x}(j))^{A_F(i,j)} \right), \tag{2}$$

where $\mathtt{Z} = \mathtt{Z}(F, \mathcal{G})$ is a normalization constant that we call the *homomorphism density* of F in $\mathcal{G}$ [55]. A node map $\mathbf{x} : [k] \to V$ is a *homomorphism* $F \to \mathcal{G}$ if $\pi_{F \to \mathcal{G}}(\mathbf{x}) > 0$, which is the case if and only if $A(\mathbf{x}(a), \mathbf{x}(b)) > 0$ for all $a, b \in [k]$ with $A_F(a, b) > 0$ (with the convention that $\zeta^0 = 1$ for all $\zeta \in \mathbb{R}$). Informally, this means that if (i, j) is a directed edge of the motif F, then $(\mathbf{x}(i), \mathbf{x}(j))$ is a directed edge of the network $\mathcal{G}$. The term 'motif sampling' refers to the problem of sampling a random homomorphism $\mathbf{x} : F \to \mathcal{G}$ according to the distribution (2).

To learn interpretable latent motifs, it is important to sample a homomorphism $\mathbf{x} : F \to \mathcal{G}$ such that $\mathbf{x}$ is injective. This ensures that the nodes $\mathbf{x}(1), \ldots, \mathbf{x}(k)$ in V that correspond to nodes $1, \ldots, k$ in the motif F through the homomorphism $\mathbf{x}$ are all distinct. When $\mathbf{x} : F \to \mathcal{G}$ is an injective homomorphism, we write $\mathbf{x} : F \hookrightarrow \mathcal{G}$. Using the subgraph of $\mathcal{G}$ that is induced by the

node set $\{\mathbf{x}(1), \dots, \mathbf{x}(k)\}$ when $\mathbf{x}$ is injective returns a k-node subgraph of $\mathcal{G}$. For convenience, we define the probability distribution

$$\pi_{F \hookrightarrow \mathcal{G}}(\mathbf{x}) := C\, \pi_{F \to \mathcal{G}}(\mathbf{x}) \cdot \mathbb{1}(\mathbf{x}(1), \dots, \mathbf{x}(k) \text{ are distinct})\,, \tag{3}$$

where $C > 0$ is a normalization constant. Injective motif sampling refers to the problem of sampling a random injective homomorphism $\mathbf{x} : F \hookrightarrow \mathcal{G}$ according to the distribution (3). The probability distribution (3) is well-defined as long as there exists an injective homomorphism $\mathbf{x} : F \to \mathcal{G}$. As a special case of interest, for a symmetric and binary motif F and a network $\mathcal{G}$, the distributions $\pi_{F \to \mathcal{G}}$ and $\pi_{F \hookrightarrow \mathcal{G}}$ are the uniform distribution among all homomorphisms $F \to \mathcal{G}$ and among all injective homomorphisms $F \hookrightarrow \mathcal{G}$, respectively. That is,

$$F,\, \mathcal{G} \text{ are symmetric and binary} \quad \Longrightarrow \quad \begin{array}{l} \pi_{F \to \mathcal{G}} = \text{Uniform}(\{\mathbf{x} : F \to \mathcal{G}\})\,, \\ \pi_{F \hookrightarrow \mathcal{G}} = \text{Uniform}(\{\mathbf{x} : F \hookrightarrow \mathcal{G}\})\,, \end{array} \tag{4}$$

which is the case for all of our examples in the main manuscript. In Appendix B, we discuss three MCMC algorithms for motif sampling and propose corresponding algorithms for injective motif sampling by combining them with rejection sampling.

A.3. **Mesoscale patches of networks.** A homomorphism $F \to \mathcal{G}$ is a node map $V(F) \to V(\mathcal{G})$ that maps the edges of a motif F to edges of a network $\mathcal{G}$, so it maps F onto a subgraph of $\mathcal{G}$. (The subgraph can be a proper subgraph of G.) It thereby maps F into $\mathcal{G}$. For each homomorphism $\mathbf{x} : F \to \mathcal{G}$ from a motif $F = ([k], A_F)$ into a network $\mathcal{G} = (V, A)$, we define a $k \times k$ matrix

$$A_{\mathbf{x}}(a, b) := A\big(\mathbf{x}(a), \mathbf{x}(b)\big) \quad \text{for all} \quad a, b \in \{1, \dots, k\}\,. \tag{5}$$

We say that $A_{\mathbf{x}}$ in (5) is the *mesoscale patch* of $\mathcal{G}$ that is induced by the homomorphism $\mathbf{x} : F \to \mathcal{G}$. The matrix $A_{\mathbf{x}}$ is specified uniquely by the homomorphism $\mathbf{x} : F \to \mathcal{G}$ and the weight matrix A. Given a $k \times k$ matrix B and a homomorphism $\mathbf{x} : F \to \mathcal{G}$, we say that the (a, b) entries of B are *on-chain* if $A_F(a, b) > 0$ and are *off-chain* otherwise. The condition $A_F(a, b) > 0$ implies that $A(\mathbf{x}(a), \mathbf{x}(b)) > 0$ by the definition of the homomorphism $\mathbf{x}$, so the on-chain entries of $A_{\mathbf{x}}$ are always positive (and are always 1 if $\mathcal{G}$ is unweighted). However, the off-chain entries of $A_{\mathbf{x}}$ are not necessarily positive, so they encode meaningful information about a network that one 'detects' with the homomorphism $\mathbf{x} : F \to \mathcal{G}$. As an illustration, suppose that F is the 6-chain motif and that $\mathcal{G} = (V, A)$ is an undirected and binary graph. For any homomorphism $\mathbf{x} : F \to \mathcal{G}$, we have

$$A_{\mathbf{x}} = \begin{bmatrix} 0 & 1 & * & * & * & * \\ 1 & 0 & 1 & * & * & * \\ * & 1 & 0 & 1 & * & * \\ * & * & 1 & 0 & 1 & * \\ * & * & * & 1 & 0 & 1 \\ * & * & * & * & 1 & 0 \end{bmatrix},$$

where each entry $*$ of $A_{\mathbf{x}}$ is either 0 or 1. In this example, the entries that we mark as 1 are the on-chain entries of $A_{\mathbf{x}}$ and the other entries are off-chain entries.

Let $\mathcal{G}_{\mathbf{x}}$ denote the induced subgraph of $\mathcal{G}$ whose node set is the image $\text{Im}(\mathbf{x}_t) = \{\mathbf{x}(a) \,|\, a \in \{1, \dots, k\}\}$ of the homomorphism $\mathbf{x} : F \to \mathcal{G}$. If $\mathbf{x}$ has k distinct nodes in its image, then the weight matrix of $\mathcal{G}_{\mathbf{x}}$ is exactly the mesoscale patch $A_{\mathbf{x}}$. However, this is not the case when $\mathbf{x}$ has fewer than k distinct nodes. In that situation, we cannot interpret the mesoscale patch $A_{\mathbf{x}}$ as the weight matrix of the induced subgraph $\mathcal{G}_{\mathbf{x}}$ of $\mathcal{G}$. (For example, see Figure 10.) This motivates us to sample an injective homomorphism $\mathbf{x} : F \to \mathcal{G}$ according to the distribution (3), instead of according to the distribution (2).

A.4. **Problem formulation for network dictionary learning (NDL).** The goal of the *NDL problem* is to learn, for a fixed integer $r \geq 1$, a set of r nonnegative matrices $\mathcal{L}_1, \dots, \mathcal{L}_r$, with size $k \times k$ and Frobenius norms of at most 1, such that

$$A_{\mathbf{x}} \approx a_1(\mathbf{x})\mathcal{L}_1 + \cdots + a_r(\mathbf{x})\mathcal{L}_r \tag{6}$$

for each injective homomorphism $\mathbf{x} : F \hookrightarrow \mathcal{G}$ for some coefficients $a_1(\mathbf{x}), \dots, a_r(\mathbf{x}) \geq 0$. For each injective homomorphism $\mathbf{x} : F \to \mathcal{G}$, this implies that one can approximate the mesoscale patch $A_{\mathbf{x}}$ of $\mathcal{G}$ that is induced by $\mathbf{x}$ as a linear combination of the r matrices $\mathcal{L}_1, \dots, \mathcal{L}_r$. We say that the tuple $(\mathcal{L}_1, \dots, \mathcal{L}_r)$ is a *network dictionary* for $\mathcal{G}$, and we say that each $\mathcal{L}_i$ is a *latent motif* of $\mathcal{G}$. We identify a network dictionary $(\mathcal{L}_1, \dots, \mathcal{L}_r)$ with the nonnegative matrix $W \in \mathbb{R}^{k^2 \times r}_{\geq 0}$ whose j^{th} column is the vectorization of the jth latent motif $\mathcal{L}_j$ for $j \in \{1, \dots, r\}$. The choice of vectorization $\mathbb{R}^{k \times k} \to \mathbb{R}^{k^2}$ is arbitrary, but we use a column-wise vectorization in Algorithm A4. One can interpret each $\mathcal{L}_i$ as the k-node weighted network with node set $\{1, \dots, k\}$ and weight matrix $\mathcal{L}_i$.

For the latent motifs $\mathcal{L}_i$ to be interpretable as subgraphs of $\mathcal{G}$, we require both their entries and the coefficients $a_i(\mathbf{x})$ to be nonnegative. The nonnegativity constraint on each latent motif $\mathcal{L}_i$ allows one to interpret each $\mathcal{L}_i$ as the weight matrix of a k-node network. Additionally, because the coefficients $a_j(\mathbf{x})$ are also nonnegative, the approximate decomposition (6) implies that $a_i(\mathbf{x})\mathcal{L}_i \lessapprox A_{\mathbf{x}}$. Therefore, if $a_i(\mathbf{x}) > 0$, any network structure (e.g., large-degree nodes, communities, and so on) in the latent motif $\mathcal{L}_i$ must also exist in $A_{\mathbf{x}}$. Therefore, one can consider the latent motifs as approximate k-node subgraphs $\mathcal{G}$ that exhibit 'typical' network structures of $\mathcal{G}$ at scale k. In the spirit of Lee and Seung [25], one can view the latent motifs as 'parts'[1] of a network $\mathcal{G}$.

As a more precise formulation of (6), consider the stochastic optimization problem

$$\underset{\substack{\mathcal{L}_1, \dots, \mathcal{L}_r \in \mathbb{R}^{k \times k}_{\geq 0} \\ \|\mathcal{L}_1\|_F, \dots, \|\mathcal{L}_r\|_F \leq 1}}{\arg\min} \; \mathbb{E}_{\mathbf{x} \sim \pi_{F \hookrightarrow \mathcal{G}}} \left[\inf_{a_1(\mathbf{x}), \dots, a_r(\mathbf{x}) \geq 0} \left\| A_{\mathbf{x}} - \sum_{i=1}^{r} a_i(\mathbf{x}) \mathcal{L}_i \right\|_F \right], \tag{7}$$

where $\pi_{F \hookrightarrow \mathcal{G}}$ is the probability distribution that we defined in (3) and $\|\cdot\|_F$ denotes the matrix Frobenius norm. The choice of the probability distribution $\pi_{F \hookrightarrow \mathcal{G}}$ for the injective homomorphisms $\mathbf{x} : F \hookrightarrow \mathcal{G}$ is natural because it becomes the uniform distribution on the set of all injective homomorphisms $F \hookrightarrow \mathcal{G}$ when the adjacency matrices of $\mathcal{G}$ and F are both unweighted. Exactly solving the NDL optimization problem (7) is computationally difficult because the objective function in it is non-convex and it is not obvious how to sample an injective homomorphism $F \hookrightarrow \mathcal{G}$ according to the distribution $\pi_{F \hookrightarrow \mathcal{G}}$ that we defined in (3). In Appendix C, we give an algorithm for NDL that approximately solves (7).

A.5. **Overview of our algorithms and their theoretical guarantees.** We now overview our algorithms and their theoretical guarantees. Our main theoretical results (which are all novel) are Theorems F.4 and F.7 for NDL and Theorems F.10 and F.14 for NDR. We summarize our algorithms and main results in Supplementary Table 1, and we compare and contrast them to the results in [13].

Algorithm NDL: Given a network $\mathcal{G}$, the NDL algorithm (see Algorithm NDL) computes a sequence $(W_t)_{t \geq 0}$ of network dictionaries (which take the form of $k^2 \times r$ matrices) of latent motifs.

[1] Lee and Seung [25] discussed a similar nonnegative decomposition in which the $A_{\mathbf{x}}$ are images of faces. In that scenario, the learned factors capture parts of human faces (such as eyes, noses, and mouths).

NDL	Sampling	Convergence		Efficient MCMC
Lyu et al. [13]	k-walks	Non-bipartite networks		✗
The present work	k-paths (Alg. IM)	Non-bipartite networks (Thm. F.4)	Bipartite networks (Thm. F.7)	✓ (Prop. F.2)

NDR	Sampling	`denoising`	Convergence	Error bound
Lyu et al. [13]	k-walks	`F`	✗	✗
The present work	k-walks<br>k-paths	`F`, `T`	✓<br>(Thm. F.10(**i**)–(**ii**))<br>(Thm. F.14(**i**)–(**ii**))	✓<br>(Thm. F.10(**iii**))<br>(Thm. F.14(**iii**))

Supplementary Table 1. A comparison of the algorithms and theoretical contributions of the present paper to those in [13]. In the table for NDR, `denoising` refers to the Boolean variable in our NDR algorithm (see Algorithm NDR). The special case of the NDR algorithm with k-walk sampling (i.e., `InjHom` = `F`) and `denoising` = `F` is the network-reconstruction algorithm in [13].

Algorithm NDR: Given a network $\mathcal{G}$, a network dictionary W, the NDR algorithm (see Algorithm NDR) computes a sequence of weighted networks $\mathcal{G}_{\text{recons}}$.

Theorem F.4: Given a non-bipartite network $\mathcal{G}$ and a choice of the parameters in Algorithm NDL, we prove that the sequence $(W_t)_{t\geq 0}$ of network dictionaries converges almost surely to the set of stationary points of the objective function in (7).

Theorem F.7: Given a bipartite network $\mathcal{G}$ and a choice of the parameters in Algorithm NDL, we prove a convergence result that is analogous to the one in Theorem F.4.

Theorem F.10: Given a non-bipartite target network $\mathcal{G}$ and a network dictionary W, we show that **(i)** the sequence of weighted reconstructed networks $\mathcal{G}_{\text{recons}}$ that we obtain using the NDR algorithm (see Algorithm NDR) converges almost surely to some limiting network and **(ii)** we obtain a closed-form expression for the weight matrix of this limiting network. We also show that **(iii)** a suitable Jaccard reconstruction error between the original network $\mathcal{G}$ and the limiting reconstructed network satisfies

$$\text{Jaccard reconstruction error} \leq \frac{\text{mesoscale approximation error}}{2(k-1)},$$

where k denotes the mesoscale parameter (i.e., the number of nodes of a k-chain motif) and the mesoscale approximation error is the mean L_1 distance between the $k \times k$ mesoscale patches of $\mathcal{G}$ and their nonnegative linear approximations from the latent motifs in W.

Theorem F.14: We show a convergence result that is analogous to the one in Theorem F.14 for a bipartite target network $\mathcal{G}$.

Appendix B. Markov-Chain Monte Carlo (MCMC) Motif-Sampling Algorithms

In Appendix A.4, we mentioned that one of the main difficulties in solving the optimization problem (7) is to directly sample an injective homomorphism $\mathbf{x} : F \hookrightarrow \mathcal{G}$ from the distribution $\pi_{F\hookrightarrow\mathcal{G}}$ (see (3)). To overcome this difficulty, we use (and extend to one new variant) the Markov-chain Monte Carlo (MCMC) algorithms that were introduced in [21]. Although the algorithms in [21] apply to networks with edge weights and/or node weights, we only use the simplified forms of them that we give in Algorithms MP and MG. Algorithm MP with the option `AcceptProb` =

`Approximate` is a novel algorithm of the present paper. Using these MCMC sampling algorithms, we generate a sequence $(\mathbf{x}_t)_{t\geq 0}$ of homomorphisms $F \to \mathcal{G}$ such that the distribution of $\mathbf{x}_t$ converges to $\pi_{F\to\mathcal{G}}$ under some mild conditions on $\mathcal{G}$ and F [13, Thm. 5.7].

Once we have an iterative motif-sampling algorithm, we combine it with a standard rejection-sampling algorithm to shrink the support of the probability distribution $\pi_{F\to\mathcal{G}}$ to injective homomorphisms $\mathbf{x} : F \hookrightarrow \mathcal{G}$. (See, e.g., [60] for background information about rejection sampling.) The key idea is to ignore (i.e., 'reject') the unwanted instances in the trajectory $(\mathbf{x}_t)_{t\geq 0}$. In our case, the instances that we reject are the homomorphisms $\mathbf{x}_t$ that are not injective. That is, we reject situations in which $\mathbf{x}_t(1), \ldots, \mathbf{x}_t(k)$ are not all distinct. In Algorithm IM, we state our algorithm for injective motif sampling.

Algorithm IM. Injective MCMC motif sampling

1: **Input:** Network $\mathcal{G} = (V, A)$, motif $F = ([k], A_F)$, and homomorphism $\mathbf{x} : F \to \mathcal{G}$
2: **While:** $\mathbf{x} : F \to \mathcal{G}$ is injective (i.e., $\mathbf{x}'(1), \ldots, \mathbf{x}'(k)$ are distinct)
3: Update $\mathbf{x}$ to a new homomorphism $F \to \mathcal{G}$ using either Algorithm MP or Algorithm MG
4: **Output:** Injective homomorphism $\mathbf{x} : F \hookrightarrow \mathcal{G}$

Algorithm IM restricts the state space of the MCMC motif-sampling algorithms (see Algorithms MG and MP) to the subset of injective homomorphisms $F \hookrightarrow \mathcal{G}$. By the strong Markov property, this restriction is a Markov chain. Therefore, Algorithm IM is an MCMC algorithm for the injective motif-sampling problem. (See Proposition F.3 for details.) When there are only a few injective homomorphisms $F \hookrightarrow \mathcal{G}$ relative to the number of homomorphisms $F \to \mathcal{G}$, the rejection step (i.e., the while loop) in Algorithm IM may take a while to terminate. (The number of rejections until termination is inversely proportional to the probability that a random homomorphism under the probability distribution $\pi_{F\to\mathcal{G}}$ is injective.) For example, this is the case when $\mathcal{G}$ is the network `Coronavirus PPI` and F is a k-chain motif with $k \geq 21$.

We now give more details about the MCMC algorithms that we employ for non-injective motif sampling. In the *pivot chain* (see Algorithm MP with `AcceptProb` = `Exact`), for each update $\mathbf{x}_t \mapsto \mathbf{x}_{t+1}$, the *pivot* $\mathbf{x}_t(1)$ first performs a random-walk move on $\mathcal{G}$ (see (8)) to move to a new node $\mathbf{x}_{t+1}(1) \in V$. It accepts this move with a suitable acceptance probability (see (9)) according to the Metropolis–Hastings algorithm (see, e.g., [61, Sec. 3.2]), so the stationary distribution is exactly the target distribution. After the move $\mathbf{x}_t(1) \mapsto \mathbf{x}_{t+1}(1)$, we sample each $\mathbf{x}_{t+1}(i) \in V$ for $i \in \{2, 3, \ldots, k\}$ successively from the conditional distribution (10). This ensures that the desired distribution $\pi_{F\to\mathcal{G}}$ in (2) is a stationary distribution of the resulting Markov chain. In the *Glauber chain* (see Algorithm MG), we select one node $i \in [k]$ of F uniformly at random, and we resample its location $\mathbf{x}_t(i) \in V(\mathcal{G})$ at time t to $\mathbf{x}_{t+1}(i) \in V$ from the conditional distribution (11) (see Supplementary Figure 1**a**). See [61, Sec. 3.3] for discussions of the Metropolis–Hastings algorithm and Glauber-chain MCMC sampling.

Let Δ denote the maximum degree (i.e., number of neighbors) of the nodes of the network $\mathcal{G} = (V, A)$. We also say that the network $\mathcal{G}$ itself has a maximum degree of Δ. The Glauber chain has an efficient local update (with a computational complexity of $O(\Delta)$). It converges quickly to the stationary distribution $\pi_{F\to\mathcal{G}}$ for networks that are dense enough so that two homomorphisms that differ at one node have a probability of at least $1/(2\Delta)$ to coincide after a single Glauber-chain update. See [21, Thm. 6.1] for a precise statement of this fact.

The pivot chain (see Algorithm MP with `AcceptProb` = `Exact`) has more computationally expensive local updates than the Glauber chain. The pivot chain has a computational complexity of $O(\Delta^{k-1})$ (as discussed in [21, Remark 5.6]), but it converges as fast as a 'lazy' random walk on a network. (In a lazy random walk, each move has a chance to be rejected; see [21, Thm.

Algorithm MP. Pivot-Chain Update

Input: Symmetric network $\mathcal{G} = (V, A)$, a k-chain motif $F = ([k], A_F)$, and homomorphism $\mathbf{x} : F \to \mathcal{G}$

Parameters: $\texttt{AcceptProb} \in \{\texttt{Exact}, \texttt{Approximate}\}$

Do: $\mathbf{x}' \leftarrow \mathbf{x}$

  If $\sum_{c \in V} A(\mathbf{x}(1), c) = 0$: **Terminate**

  Else:

    Sample $\iota \in V$ at random from the distribution

$$p_1(w) = \frac{A(\mathbf{x}(1), w)}{\sum_{c \in V} A(\mathbf{x}(1), c)} \,, \quad w \in V \tag{8}$$

    Compute the acceptance probability $\alpha \in [0, 1]$ by

$$\alpha \leftarrow \begin{cases} \min\left\{ \frac{\sum_{c \in [n]} A^{k-1}(\iota, c)}{\sum_{c \in [n]} A^{k-1}(\mathbf{x}(1), c)} \frac{\sum_{c \in V} A(c, \mathbf{x}(1))}{\sum_{c \in V} A(\mathbf{x}(1), c)}, \, 1 \right\} \,, & \text{if} \quad \texttt{AcceptProb} = \texttt{Exact} \\ \min\left\{ \frac{\sum_{c \in V} A(c, \mathbf{x}(1))}{\sum_{c \in V} A(\mathbf{x}(1), c)}, \, 1 \right\} \,, & \text{if} \quad \texttt{AcceptProb} = \texttt{Approximate} \end{cases} \tag{9}$$

    Sample $U \in [0, 1]$ uniformly at random, independently of everything else

    $\iota \leftarrow \mathbf{x}(1)$ if $U > \lambda$ and $\mathbf{x}'(1) \leftarrow \iota$

    For $i = 2, 3, \ldots, k$:

      Sample $\mathbf{x}'(i) \in V$ from the distribution

$$p_i(w) = \frac{A(\mathbf{x}(i-1), w)}{\sum_{c \in V} A(\mathbf{x}(i-1), c)} \,, \quad w \in V \tag{10}$$

Output: Homomorphism $\mathbf{x}' : F \to \mathcal{G}$

Algorithm MG. Glauber-Chain Update

Input: Network $\mathcal{G} = (V, A)$, a k-chain motif $F = ([k], A_F)$, and homomorphism $\mathbf{x} : F \to \mathcal{G}$

Do: Sample $v \in [k]$ uniformly at random

  Sample $z \in V$ at random from the distribution

$$p(w) = \frac{1}{Z} \left(\prod_{u \in [k]} A(\mathbf{x}(u), w)^{A_F(u,v)} \right) \left(\prod_{u \in [k]} A(w, \mathbf{x}(u))^{A_F(v,u)} \right) \,, \quad w \in V \tag{11}$$

  where $Z = \sum_{c \in V} \left(\prod_{u \in [k]} A(\mathbf{x}(u), c)^{A_F(u,v)} \right) \left(\prod_{u \in [k]} A(c, \mathbf{x}(u))^{A_F(v,u)} \right)$ is the normalization constant

  Define a new homomorphism $\mathbf{x}' : F \to \mathcal{G}$ by $\mathbf{x}'(w) = z$ if $w = v$ and $\mathbf{x}'(w) = \mathbf{x}(w)$ otherwise

Output: Homomorphism $\mathbf{x}' : F \to \mathcal{G}$

6.2].) In our computational experiments, we find that the Glauber chain is slow, especially for sparse networks (e.g., for `COVID PPI`, which has an edge density of 0.0010, and `UCLA`, which has an edge density of 0.0037) and that the pivot chain is too expensive to compute for chain motifs with $k \geq 21$. As a compromise, to simultaneously have low computational complexity and fast convergence (it is as fast as the standard random walk), we employ an approximate pivot chain, which is Algorithm MP with the option `AcceptProb` = `Approximate`. Specifically, we compute the acceptance probability α in (9) only approximately and thereby reduce the computational cost to $O(\Delta)$. Our compromise, which we discuss in the next paragraph, is that the stationary distribution of the approximate pivot chain may be slightly different from our target distribution $\pi_{F\to\mathcal{G}}$.

Define a probability distribution $\hat{\pi}_{F\to\mathcal{G}}$ on the set of all node maps $\mathbf{x} : [k] \to V$ by

$$\hat{\pi}_{F\to\mathcal{G}}(\mathbf{x}) := \frac{\prod_{i=1}^{k} A(\mathbf{x}(i-1), \mathbf{x}(i))}{|V| \sum_{y_2,\dots,y_k \in V} A(\mathbf{x}(1), y_2) \prod_{i=3}^{k} A(y_{i-1}, y_i)} . \tag{12}$$

According to Proposition F.2, the stationary distribution of the approximate pivot chain is (12). The distribution (12) is different from the desired target distribution $\pi_{F\to\mathcal{G}}$. Specifically, $\pi_{F\to\mathcal{G}}(\mathbf{x})$ is proportional only to the numerator in (12); the sum in the denominator of (12) is a weighted count of the homomorphisms $\mathbf{y} : F \to \mathcal{G}$ for which $\mathbf{y}(1) = \mathbf{x}(1)$. Therefore, under $\hat{\pi}_{F\to\mathcal{G}}$, we penalize the probability of each homomorphism $\mathbf{x} : F \to \mathcal{G}$ according to the number of k-walks of $\mathcal{G}$ that start from $\mathbf{x}(1) \in V$. (The exact acceptance probability in (9) neutralizes this penalty.) It follows that $\hat{\pi}_{F\to\mathcal{G}}$ is close to $\pi_{F\to\mathcal{G}}$ when the k-step-walk counts that start from each node of $\mathcal{G}$ do not differ too much for different nodes. For example, on degree-regular networks like lattices, such counts do not depend on the starting node, and it thus follows that $\hat{\pi}_{F\to\mathcal{G}} = \pi_{F\to\mathcal{G}}$. Nevertheless, despite the potential discrepancy between $\pi_{F\to\mathcal{G}}$ and $\hat{\pi}_{F\to\mathcal{G}}$, the approximate pivot chain gives good results for the reconstruction and denoising experiments that we showed in Figures 5 and 8 of the main manuscript.

Appendix C. Algorithm for Network Dictionary Learning (NDL)

C.1. **Algorithm overview and statement.** The essential idea behind our algorithm for NDL (see Algorithm NDL) is as follows. Suppose that we compute all possible injective homomorphisms $\mathbf{x}_1, \dots, \mathbf{x}_M : F \hookrightarrow \mathcal{G}$ and their corresponding mesoscale patches $A_{\mathbf{x}_t}$ for $t \in \{1, \dots, M\}$. These M mesoscale patches of $\mathcal{G}$ form the data set in which we apply a dictionary-learning algorithm. To do this, we column-wise vectorize each of these $k \times k$ matrices (using Algorithm A4) and obtain a $k^2 \times M$ data matrix X, and we then apply nonnegative matrix factorization (NMF) [25] to obtain a $k^2 \times r$ nonnegative matrix W for some fixed integer $r \geq 1$ to yield an approximate factorization $X \approx WH$ for some nonnegative matrix H. From this procedure, we approximate each column of X by the nonnegative linear combination of the r columns of W with coefficients that are given by entries of the rth column of H. Therefore, if we let $\mathcal{L}_i$ be the $k \times k$ matrix that we obtain by reshaping the ith column of W (using Algorithm A5), then $(\mathcal{L}_1, \dots, \mathcal{L}_r)$ is an approximate solution of (7). We give the precise meaning of 'approximate solution' in Theorems F.4 and F.7.

The scheme in the paragraph above requires one to store all M mesoscale patches, entailing a memory requirement that is at least of order $k^2 M$, where M is the number of all possible injective homomorphisms $F \hookrightarrow \mathcal{G}$. Because M grows with the number of nodes of $\mathcal{G}$, we need unbounded memory to handle arbitrarily large networks. To address this issue, Algorithm NDL implements the above scheme in the setting of 'online learning', where subsets (so-called 'minibatches') of data arrive in a sequential manner and one does not store previous subsets of the data before processing new subsets. Specifically, at each iteration $t \in \{1, 2, \dots, T\}$, we process a sample

Algorithm NDL. Network Dictionary Learning (NDL)

Input: Network $\mathcal{G} = (V, A)$

Parameters: $F = ([k], A_F)$ (a k-chain motif), $T \in \mathbb{N}$ (the number of iterations), $N \in \mathbb{N}$ (the number of homomorphisms per iteration), $r \in \mathbb{N}$ (the number of latent motifs), $\lambda \geq 0$ (the coefficient of an L_1-regularizer)

Options: `MCMC` $\in$ {`Pivot`, `PivotApprox`, `Glauber`}

Requirement: There exists at least one injective homomorphism $F \hookrightarrow \mathcal{G}$

Initialization:

  Sample a homomorphism $\mathbf{x} : F \to \mathcal{G}$ using the rejection sampling (see Algorithm A3)

  W = matrix of size $k^2 \times r$ with independent entries that we sample uniformly from $[0, 1]$

  P_0 = matrix of size $r \times r$ whose entries are 0

  Q_0 = matrix of size $r \times k^2$ whose entries are 0

For $t = 1, 2, \dots, T$**:**

  MCMC update and sampling mesoscale patches:

    Successively generate N injective homomorphisms $\mathbf{x}_{N(t-1)+1}, \mathbf{x}_{N(t-1)+2}, \dots, \mathbf{x}_{Nt}$ by applying Algorithm IM with

Algorithm MP with `AcceptProb` = `Exact`	if	`MCMC` = `Pivot`
Algorithm MP with `AcceptProb` = `Approximate`	if	`MCMC` = `PivotApprox`
Algorithm MG with `AcceptProb` = `Glauber`	if	`MCMC` = `Glauber`

    For $s = N(t-1) + 1, \dots, Nt$:

      $A_{\mathbf{x}_s} \leftarrow k \times k$ mesoscale patch of $\mathcal{G}$ that is induced by $\mathbf{x}_s$ (see (5))

      $X_t \leftarrow k^2 \times N$ matrix whose j^{th} column is $\texttt{vec}(A_{\mathbf{x}_\ell})$ with $\ell = N(t-1) + j$ (where $\texttt{vec}(\cdot)$ denotes the vectorization operator that we defined in Algorithm A4)

  Single iteration of online nonnegative matrix factorization:

$$\begin{cases} H_t \leftarrow \arg\min_{H \in \mathbb{R}^{r \times N}_{\geq 0}} \|X_t - W_{t-1}H\|_F^2 + \lambda \|H\|_1 & \text{(using Algorithm A1)} \\ P_t \leftarrow (1 - t^{-1})P_{t-1} + t^{-1} H_t H_t^T \\ Q_t \leftarrow (1 - t^{-1})Q_{t-1} + t^{-1} H_t X_t^T \\ W_t \leftarrow \arg\min_{W \in \mathcal{C}^{\text{dict}} \subseteq \mathbb{R}^{k^2 \times r}_{\geq 0}} \left(\operatorname{tr}(W P_t W^T) - 2\operatorname{tr}(W Q_t)\right) & \text{(using Algorithm A2)}, \end{cases} \tag{13}$$

where $\mathcal{C}^{\text{dict}} = \{W \in \mathbb{R}^{k^2 \times r}_{\geq 0} \mid \text{columns of } W \text{ have a Frobenius norm of at most } 1\}$

Output: Network dictionary $W_T \in \mathbb{R}^{k^2 \times r}_{\geq 0}$

matrix X_t that is smaller than the full matrix X and includes only $N \ll M$ mesoscale patches, where one can take N to be independent of the network size. Instead of using a standard NMF algorithm for a fixed matrix [51], we use an 'online' NMF algorithm [13, 52] that one can use on sequences of matrices, where the intermediate dictionary matrices W_t that we obtain by factoring the sample matrix X_t typically improves as we iterate (see [13, 52]). In Algorithm NDL, we give a complete implementation of the NDL algorithm.

We now explain how our NDL algorithm works. It combines one of the three MCMC algorithms — a pivot chain (in which we use Algorithm MP with `AcceptProb` = `Exact`), an approximate pivot chain (in which we use Algorithm MP with `AcceptProb` = `Approximate`), and a Glauber chain (in which we use Algorithm MG) — for injective motif sampling that we

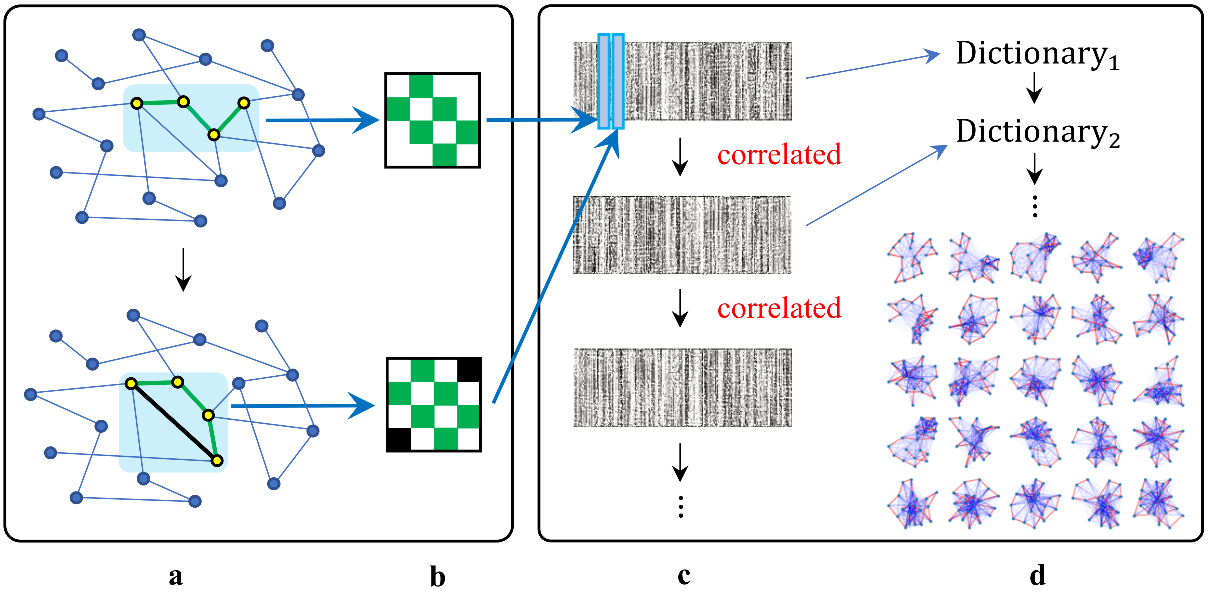

Supplementary Figure 1. Illustration of our network dictionary learning (NDL) algorithm (see Algorithm NDL). (**a**) Homomorphisms $\mathbf{x}_t : F \to \mathcal{G}$ from a k-chain motif into a target network $\mathcal{G}$ evolve as a Markov chain to yield a sequence of k-chain subgraphs (the green edges) in $\mathcal{G}$. (**b**) Each copy of the k-chain motif in $\mathcal{G}$ induces a k-node subgraph (i.e., the mesoscale patch $A_{\mathbf{x}_t}$ that we defined in (5)). (**c**) We form a sequence of matrices X_t of size $k^2 \times N$, where the N columns of each X_t are vectorizations of the N consecutive $k \times k$ mesoscale patches in panel **b**. The matrices $X_1, X_2, \ldots$ are correlated with each other because we sample their columns from the Markov chain $\mathbf{x}_t$. (**d**) Using an online nonnegative matrix factorization (NMF) algorithm, we progressively learn the desired number of latent motifs as the data matrix of mesoscale patches X_t arrives.

presented in Appendix B with the online NMF algorithm from [13]. Suppose that we have an undirected and unweighted graph $\mathcal{G} = (V, A)$ and a k-chain motif $F = ([k], A_F)$. Furthermore, assume that we satisfy the requirement in Algorithm NDL that there exists at least one injective homomorphism $F \hookrightarrow \mathcal{G}$. At each iteration $t \in \{1, 2, \ldots, T\}$, the injective MCMC motif-sampling algorithm generates a sequence $\mathbf{x}_s : F \to \mathcal{G}$ of N injective homomorphisms and corresponding mesoscale patches $A_{\mathbf{x}_s}$ (see Supplementary Figure 1**a**). We summarize this sequence in the $k^2 \times N$ data matrix X_t. The online NMF algorithm in (13) learns a nonnegative factor matrix W_t of size $k^2 \times r$ by improving the previous factor matrix W_{t-1} by using the new data matrix X_t. It is an 'online' NMF algorithm because it factorizes a sequence $(X_t)_{t \in \{1,\ldots,T\}}$ of data matrices, rather than a single matrix as in conventional NMF algorithms [51]. As it proceeds, the algorithm only needs to store auxiliary matrices P_t and Q_t of fixed sizes $r \times r$ and $r \times k^2$, respectively; it does not need the previous data matrices $X_1, \ldots, X_{t-1}$. Therefore, NDL is efficient in memory and scales well with network size. It is also applicable to time-dependent networks because of its online nature, although we do not study such networks in the present paper.

In (13), we solve convex optimization problems to find matrices $H_t \in \mathbb{R}^{r \times N}$ and $W_t \in \mathbb{R}^{k^2 \times r}$. The subproblem in (13) of computing H_t is a 'coding problem'. Given two matrices X_t and W_{t-1}, we seek to find a factor matrix (i.e., a 'code matrix') H_t such that $X_t \approx W_{t-1} H_t$. The parameter $\lambda \geq 0$ is an L_1-regularizer, which encourages H_t to have a small L_1 norm. One can solve the coding problem efficiently by using Algorithm A1 or one of a variety of existing algorithms (e.g., layer-wise adaptive-rate scaling (LARS) [62], LASSO [63], or feature-sign search [64]). The second and third lines in (13) update the 'aggregate matrices' $P_{t-1} \in \mathbb{R}^{r \times r}$ and $Q_{t-1} \in \mathbb{R}^{r \times k^2}$

by taking a weighted average of them with the new information $X_t H_t^T \in \mathbb{R}^{r\times r}$ and $H_t X_t^T$, respectively. We weight the old aggregate matrices by $1 - t^{-1}$ and the new information by t^{-1}. By induction, $P_t = t^{-1}\sum_{s=1}^{t} H_s H_s^T$ and $Q_t = t^{-1}\sum_{s=1}^{t} H_t X_t^T$. We use the updated aggregate matrices, P_t and Q_t, in the subproblem in (13) of computing W_t. The subproblem in (13) of computing W_t is a constrained quadratic problem; we can solve it using projected gradient descent (see Algorithm A2). In all of our experiments, we take the compact and convex constraint set $\mathbb{R}_{\geq 0}^{k^2\times r}$ to be the set of $W \in \mathbb{R}_{\geq 0}^{k^2\times r}$ whose columns have a Frobenius norm of at most 1 (as required in (7)).

C.2. **Dominance scores of latent motifs.** In this subsection, we introduce a quantitative measurement of the 'prevalence' of latent motifs in the network dictionary W_T that we compute using NDL (see Algorithm NDL) for a network $\mathcal{G}$.

Given a network $\mathcal{G}$ and a k-chain motif, recall that the output of the NDL algorithm is a network dictionary W_T of r latent motifs $\mathcal{L}_1, \ldots, \mathcal{L}_r$ of size $k \times k$. Recall as well that the NDL algorithm yields data matrices $X_1, \ldots, X_T$ of size $k^2 \times N$. Suppose that we have code matrices $H_1^\star, \ldots, H_T^\star$ such that $X_t \approx W_T H_t^\star$ for all $t \in \{1, \ldots, T\}$. More precisely, we let

$$H_t^\star := \arg\min_{H\geq 0}(\|X_t - W_T H\|^2 + \lambda\|H\|_1)\,, \tag{14}$$

where we take the arg min over all $H \in \mathbb{R}_{\geq 0}^{k^2\times N}$ The columns of $H_t^\star$ encode how to nonnegatively combine the latent motifs in W_T to approximate the mesoscale patches in $X_t \in \mathbb{R}_{\geq 0}^{k^2\times N}$, so the rows of $H_t^\star$ encode the linear coefficients of each latent motif in W_T that we use to approximate the columns of X_t. Consequently, the means of the Euclidean norms of the rows of H_t for each $t \in \{1, \ldots, T\}$ encode the mean prevalences in $\mathcal{G}$ of the latent motifs in W_T. This motivates us to consider the mean Gramian matrix [65]

$$P_T^\star := \frac{1}{T}\sum_{t=1}^{T} H_t^\star (H_t^\star)^T \in \mathbb{R}^{r\times r}\,.$$

The square root of the diagonal entries of $P_T^\star$ yield the mean prevalences in $\mathcal{G}$ of the latent motifs in W_T. Accordingly, for each $i \in \{1, \ldots, r\}$, we define the *dominance score* of the latent motif $\mathcal{L}_i$ to be $\sqrt{P_T^\star(i,i)}$.

Computing $P_T^\star$ requires us to store the previous data matrices $X_1, \ldots, X_T$ and to determine $H_1^\star, \ldots, H_T^\star$ by solving (14) for $t \in \{1, \ldots, T\}$. This way of computing $P_T^\star$ is very expensive because of its extensive memory and computational requirements. To address this issue, we instead use the aggregate matrix P_T that we compute as part of Algorithm NDL. We then do not require an extra computation. Note that

$$P_T = \frac{1}{T}\sum_{t=1}^{T} H_t H_t^T\,,$$

where $H_t = \arg\min_{H\geq 0}(\|X_t - W_{t-1}H\|^2 + \lambda\|H\|_1) \in \mathbb{R}_{\geq 0}^{r\times N}$ is the code matrix. The matrix P_T is an approximation of $P_T^\star$ because the defining equation of H_t is the same as that of $H_t^\star$ in (14) with W_T replaced by W_{t-1}. The approximation error of using P_T instead of $P_T^\star$ vanishes as $T \to \infty$ under mild conditions. Specifically, under the hypotheses of Theorems F.4 and F.7, the network dictionary W_t converges almost surely to some limiting dictionary. It follows that $\|P_T^\star - P_T\|_F \to 0$ almost surely as $T \to \infty$.

C.3. **Community sizes in subgraph samples and latent motifs.** By comparing the 21-node latent motifs of UCLA and CALTECH (we extract $r = 25$ of each) in Figure 4 of the main manuscript, we observe that most latent motifs of CALTECH have larger communities than those of UCLA. To what extent does the community structure of the latent motifs carry over to the subgraph samples in these networks? Latent motifs are k-node networks with nonnegative edge weights, so we can examine this question quantitatively by performing community detection using a standard approach on these k-node networks.

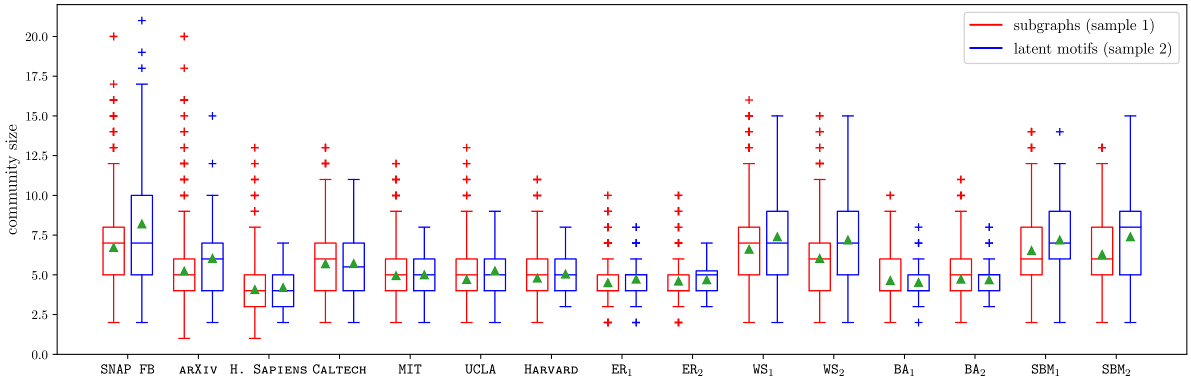

	SNAP FB	ARXIV	H. SAPIENS	CALTECH	MIT	UCLA	HARVARD	ER_1	ER_2	WS_1	WS_2	BA_1	BA_2	SBM_1	SBM_2
p-value	(0.388)	(0.119)	(0.477)	(0.734)	(0.717)	(0.143)	(0.563)	(0.108)	(0.382)	(0.334)	(0.131)	(0.565)	(0.368)	(0.209)	(0.014)
sample 1	29746	38241	49134	35127	40425	42517	41701	44399	43419	30257	33169	43106	42300	30649	31818
sample 2	64	87	125	92	105	100	104	111	112	71	73	116	112	73	71

SUPPLEMENTARY FIGURE 2. A comparison of community sizes in subgraphs and latent motifs. We compare box plots of community sizes for 10000 sampled subgraphs that are induced by uniformly random paths with $k = 21$ nodes (in red) to corresponding box plots from $r = 25$ latent motifs of $k = 21$ nodes for various real-world and synthetic networks. We obtain communities of the subgraphs and latent motifs by using the Louvain modularity-maximization algorithm [66]. The triangles inside the boxes indicate the sample means. Under each network label, we show the p-value of Mood's median test [67] and the number of samples in each population.

To detect communities, we use the locally greedy Louvain method for modularity maximization [66]. For most of our example networks, the community-size statistics of the learned latent motifs are close approximations of the corresponding statistics for the subgraph samples from the networks. In Supplementary Figure 2, we compare box plots of community sizes of 10000-node subgraphs that are induced by uniformly randomly sampled k-paths to the corresponding box plots from community detection of $r = 25$ latent motifs of various networks. In our calculations, the median community sizes in the subgraphs and latent motifs differ by 2 for the network SBM_2; differ by 1 for ARXIV, WS_2, and SBM_1; differ by 0.5 for CALTECH; and coincide for the other networks. Our experiments also demonstrate that there is no statistically significant difference between the medians of the two samples, except for SBM_2 at significance level 0.1. See Appendix E for more details.

C.4. **Latent motifs of networks at various mesoscales.** As we discussed in Appendix C.2, we associate a scalar 'dominance score' to each latent motif to measure its total contribution in our reconstruction of the sampled k-node subgraphs. In Supplementary Figure 3, we show the two most-dominant latent motifs (i.e., the two with the largest dominance scores) that we learn from each of the example networks at various scales (specifically, for $k = 6$, $k = 11$, $k = 21$, and $k = 51$) when we use a dictionary with $r = 25$ latent motifs.

For each network, as we increase the scale parameter k, various mesoscale structures emerge in the latent motifs in Supplementary Figure 3. For instance, SNAP FB, ARXIV, and WS_1 all have

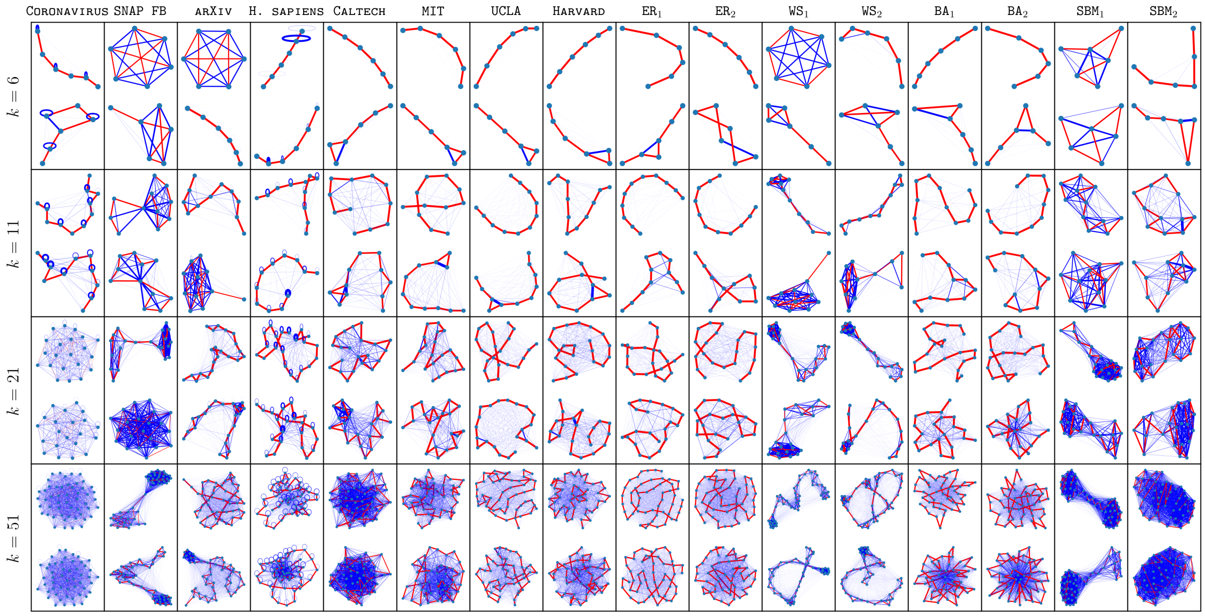

Supplementary Figure 3. The two most dominant latent motifs of various networks at several scales. The latent motifs that we learn from our 16 networks (eight real-world networks and eight synthetic networks, which include two distinct instantiations of each of four random-graph models) at four different scales (specifically, for $k = 6$, $k = 11$, $k = 21$, and $k = 51$), have distinct mesoscale structures in the networks. Using NDL, we learn network dictionaries of $r = 25$ latent motifs with k nodes for each of the 16 networks. For each network at each scale, we show the (top) first and (bottom) second most-dominant latent motif from each dictionary. See Appendix C.2 for details about how we measure latent-motif dominance.

fully connected top (i.e., most-dominant) latent motifs at scale $k = 6$ but their second most-dominant latent motifs are distinct. In SNAP FB, Caltech, and MIT at scales $k \in \{6, 11, 21\}$ and in the BA networks at scales $k \in \{11, 21, 51\}$, the two most-dominant latent motifs in Supplementary Figure 3 have nodes that are adjacent to many other nodes of the latent motif. Hubs (i.e., nodes that are adjacent to many other nodes) are characteristic of both BA networks (which have heavy-tailed degree distributions) [37] and most social networks (which typically have heavy-tailed degree distributions) [1]. We also observe hubs in the network dictionaries of the latent motifs of the Facebook networks UCLA and Harvard (see Supplementary Figure 8).

The community sizes (i.e., the numbers of nodes) in latent motifs reflect the community sizes of actual subgraphs in a network. (See Supplementary Figure 2 in Appendix C.3.) The type of community structure that we examine is different from typical network community structure. For example, consider the WS networks. The top latent motif of the network WS_1 at scale $k = 6$ is fully connected, but the top latent motif of WS_2 is not fully connected because of its larger rewiring probability. At larger scales (i.e., for larger k), both WS networks have latent motifs with multiple communities. The WS networks have locally densely connected nodes on a ring of nodes and random 'shortcut' edges that can connect distant nodes of the ring. Therefore, when one samples a k-path uniformly at random, it is very likely to use at least one shortcut edge. When a k-path uses a shortcut edge, we expect the resulting induced subgraph to have two distinct densely connected communities. This local 'community structure' in the WS networks is rather different than standard types of community structure [28, 29]. Although we do observe such community structure in subgraphs that are induced by k-paths (see, e.g., Figure 2 of the main manuscript), this observation does not imply that the entire node set of the WS networks is

partitioned into a few communities. We also see the difference between our mesoscale structures and community structure by examining the latent motifs of the SBM networks at different scales in Supplementary Figure 3. The SBM networks have three (equal-sized) communities by construction, but their latent motifs do not have three communities at any of the scales, because the uniformly sampled k-paths do not always intersect with all three communities. For example, the six 20-paths from $\texttt{SBM}_1$ in Figure 2 of the main manuscript intersect with only one or two of the network's planted communities.

Appendix D. Algorithm for Network Denoising and Reconstruction (NDR)

D.1. **Algorithm overview and statement.** The standard pipeline for image denoising and reconstruction [22, 23, 53] is to uniformly randomly sample a large number of $k \times k$ overlapping patches of an image and then average their associated approximations at each pixel to obtain a reconstructed version of the original image. (See the Methods section of the main manuscript for more details about image reconstruction.) A network analogue of this pipeline proceeds as follows. Given a network $\mathcal{G} = (V, A)$, a k-chain motif $F = ([k], A_F)$, and a network dictionary with latent motifs $(\mathcal{L}_1, \ldots, \mathcal{L}_r)$, we compute a weighted network $\mathcal{G}_{\text{recons}} = (V, A_{\text{recons}})$. To do this, we first uniformly randomly sample a large number T of (not necessarily injective) homomorphisms $\mathbf{x}_t : F \to \mathcal{G}$ and determine the corresponding mesoscale patches $A_{\mathbf{x}_0}, \ldots, A_{\mathbf{x}_T}$ using (5). We then approximate each mesoscale patch $A_{\mathbf{x}_t}$ by a nonnegative linear combination $\hat{A}_{\mathbf{x}_t}$ of the latent motifs $\mathcal{L}_i$. Finally, for each $x, y \in V$, we define $A_{\text{recons}}(x, y)$ as the mean of $\hat{A}_{\mathbf{x}_t}(a, b)$ over all $t \in \{0, \ldots, T\}$ and all $a, b \in \{1, \ldots, k\}$ such that $\mathbf{x}_t(a) = x$ and $\mathbf{x}_t(b) = y$.

Our network denoising and reconstruction (NDR) algorithm (see Algorithm NDR) uses the idea in the preceding paragraph. Suppose that we have a network $\mathcal{G} = (V, A)$, a k-chain motif $F = ([k], A_F)$, and a network dictionary W that consists of r nonnegative $k \times k$ matrices $\mathcal{L}_1, \ldots, \mathcal{L}_r$. We provide two options to reconstruct $\mathcal{G}$. In one option ($\texttt{InjHom} = \texttt{F}$), we use uniformly random homomorphisms from the distribution $\pi_{F \to \mathcal{G}}$ in (2). In the other option ($\texttt{InjHom} = \texttt{T}$), we use only the injective homomorphisms, so we instead use the distribution $\pi_{F \hookrightarrow \mathcal{G}}$ in (3). The latter option has a larger computational cost, but it has better theoretical properties for the NDL algorithm (see Algorithm NDL). To sketch how the NDR algorithm with $\texttt{InjHom} = \texttt{F}$ works, suppose that we sample homomorphisms $\mathbf{x}_0, \ldots, \mathbf{x}_T$ from the distribution $\pi_{F \to \mathcal{G}}$. For each $t \geq 0$, we approximate the mesoscale patch $A_{\mathbf{x}_t}$ (see (5)) by a nonnegative linear combination of latent motifs $\mathcal{L}_i$ and we then take the mean of the values of each entry $A(a, b)$ for all $t \in \{1, \ldots, T\}$. However, because sampling a homomorphism $\mathbf{x}_t : F \to \mathcal{G}$ from $\pi_{F \to \mathcal{G}}$ is not as straightforward as uniformly randomly sampling $k \times k$ patches of an image, we generate a sequence $(\mathbf{x}_t)_{t \in \{0, \ldots, T\}}$ of homomorphisms using an MCMC motif-sampling algorithm (see Algorithms MG and MP). The NDR algorithm with $\texttt{InjHom} = \texttt{T}$ works similarly, but it uses injective homomorphisms that are generated from the injective MCMC motif-sampling algorithm (see Algorithm IM).

For network reconstruction, it is important to sample homomorphisms $\mathbf{x}_1, \ldots, \mathbf{x}_T : F \to \mathcal{G}$ that cover an entire network $\mathcal{G}$ (or at least a large portion of it). A node x of G is 'covered' by the homomorphisms $\mathbf{x}_1, \ldots, \mathbf{x}_T$ if it is contained in the image of $\mathbf{x}_t$ for some $t \in \{1, \ldots, T\}$. In Proposition F.9, we show that one can cover all nodes of $\mathcal{G}$ by the images of injective homomorphisms $F \hookrightarrow \mathcal{G}$ if $2(k-1) \leq \text{diam}(\mathcal{G})$ if $\mathcal{G}$ is symmetric and connected. However, even when this inequality is satisfied, we have to sample more homomorphisms using one of our MCMC motif-sampling algorithms (see Algorithms MG and MP) to cover the same portion of the network $\mathcal{G}$ than when we use all sampled homomorphisms. This gives a computational advantage to using $\texttt{InjHom} = \texttt{F}$ instead of $\texttt{InjHom} = \texttt{T}$ in our NDR algorithm.

Algorithm NDR. Network Denoising and Reconstruction (NDR)

Input: Network $\mathcal{G} = (V, A)$, network dictionary $W \in \mathbb{R}^{k^2 \times r}_{\geq 0}$

Parameters: $F = ([k], A_F)$ (a k-chain motif), $T \in \mathbb{N}$ (number of iterations), $\lambda \geq 0$ (the coefficient of an L_1-regularizer), $\theta \in [0, 1]$ (an edge threshold)

Options: `denoising` $\in \{$`T`, `F`$\}$, `MCMC` $\in \{$`Pivot`, `PivotApprox`, `Glauber`$\}$, `InjHom` $\in \{$`T`, `F`$\}$

Requirement: There exists at least one homomorphism $F \to \mathcal{G}$

Initialization:

A_{recons}, $A_{\text{count}} : V^2 \to \{0\}$ (matrices with 0 entries)

Sample a (not necessarily injective) homomorphism $\mathbf{x}_0 : F \to \mathcal{G}$ using the rejection-sampling algorithm in Algorithm A3

For $t = 1, 2, \ldots, T$**:**

MCMC update and mesoscale patch extraction:

$\mathbf{x}_t \leftarrow$ Updated homomorphism that we obtain by applying

$$\begin{array}{rll} \text{Algorithm MP with } \texttt{AcceptProb} = \texttt{Exact} & \text{if} & \texttt{MCMC} = \texttt{Pivot} \\ \text{Algorithm MP with } \texttt{AcceptProb} = \texttt{Approximate} & \text{if} & \texttt{MCMC} = \texttt{PivotApprox} \\ \text{Algorithm MG with } \texttt{AcceptProb} = \texttt{Glauber} & \text{if} & \texttt{MCMC} = \texttt{Glauber} \end{array}$$

(If `InjHom` = `T`, set $\mathbf{x}_t \leftarrow$ Updated injective homomorphism by applying Algorithm IM with the specified MCMC algorithm.)

$A_{\mathbf{x}_t} \leftarrow k \times k$ mesoscale patch of $\mathcal{G}$ that is induced by $\mathbf{x}_t$ (see (5))

$X_t \leftarrow k^2 \times 1$ matrix that we obtain by vectorizing $A_{\mathbf{x}_t}$ (using Algorithm A4)

Mesoscale reconstruction:

$$\begin{cases} \widetilde{X}_t \leftarrow X_t \text{ and } \widetilde{W} \leftarrow W & \text{if } \texttt{denoising} = \texttt{F} \\ \widetilde{X}_t \leftarrow (X_t)_{\text{off}} \text{ and } \widetilde{W} \leftarrow (W)_{\text{off}} \text{ using Algorithm 2a} & \text{if } \texttt{denoising} = \texttt{T} \end{cases}$$

$H_t \leftarrow \underset{H \in \mathbb{R}^{r \times 1}_{\geq 0}}{\arg\min} (\|\widetilde{X}_t - \widetilde{W} H\|_F^2 + \lambda \|H\|_1)$ and $\hat{X}_t \leftarrow \widetilde{W} H_t$

$\hat{A}_{\mathbf{x}_t;W} \leftarrow k \times k$ matrix that we obtain by reshaping the $k^2 \times 1$ matrix $\hat{X}_t$ using Algorithm A5

Update reconstruction:

For $a, b \in \{1, \ldots, k\}$:

If (`denoising` = `F` or $A_F(a, b) = 0$):

$$A_{\text{count}}(\mathbf{x}_t(a), \mathbf{x}_t(b)) \leftarrow A_{\text{count}}(\mathbf{x}_t(a), \mathbf{x}_t(b)) + 1$$

$$j \leftarrow A_{\text{count}}(\mathbf{x}_t(a), \mathbf{x}_t(b))$$

$$A_{\text{recons}}(\mathbf{x}_t(a), \mathbf{x}_t(b)) \leftarrow (1 - j^{-1}) A_{\text{recons}}(\mathbf{x}_t(a), \mathbf{x}_t(b)) + j^{-1} \hat{A}_{\mathbf{x}_t;W}(\mathbf{x}_t(a), \mathbf{x}_t(b))$$

Output: Reconstructed network $\mathcal{G}_{\text{recons}} = (V, A_{\text{recons}})$

Algorithm 2a. Off-Chain Projection

Input: Matrix $Y \in \mathbb{R}^{k^2 \times m}$, k-chain motif $F = ([k], A_F)$

Do: Let Y' be a $k \times k \times m$ tensor that we obtain by reshaping each column of Y using Algorithm A5

Let Y'' be a $k \times k \times m$ tensor that we obtain from Y' by calculating

$$Y''(a,b,c) = Y'(a,b,c)\mathbb{1}(A_F(a,b) = 0) \quad \text{for all} \quad a, b \in \{1,\dots,k\} \text{ and } c \in \{1,\dots,m\}$$

Let Y_{off} be a $k^2 \times m$ matrix that we obtain from Y'' by vectorizing each of its slices using Algorithm A4: $Y''[:,:,c]$ for all $c \in \{1,\dots,m\}$

Output: Matrix $(Y)_{\text{off}} \in \mathbb{R}^{k^2 \times m}$

Despite the computational disadvantage of using `InjHom` = `T`, this choice has a nice theoretical advantage. Recall that the NDL algorithm (see Algorithm NDL) computes latent motifs $\mathcal{L}_1, \dots, \mathcal{L}_r$ from mesoscale patches $A_{\mathbf{y}_1}, \dots, A_{\mathbf{y}_M}$ of $\mathcal{G}$ for injective homomorphisms $\mathbf{y}_t : F \to \mathcal{G}$ for $t \in \{1,\dots,M\}$ such that $\mathcal{L}_1, \dots, \mathcal{L}_r$ give an approximate solution of (7). Consequently, when linearly approximating a mesoscale patch $A_{\mathbf{x}}$ of $\mathcal{G}$, these latent motifs $\mathcal{L}_1, \dots, \mathcal{L}_r$ are less effective if the homomorphism $\mathbf{x}$ is non-injective than if $\mathbf{x}$ is injective. We need to linearly approximate multiple mesoscale patches $A_{\mathbf{x}_1}, \dots, A_{\mathbf{x}_T}$ for homomorphisms $\mathbf{x}_t : F \to \mathcal{G}$ for $t \in \{1,\dots,T\}$, so we expect the reconstructed network that we obtain using Algorithm NDR with only injective homomorphisms to be more accurate than when using all sampled homomorphisms. In Theorem F.10**(iii)**, we obtain an upper bound for the Jaccard reconstruction error (which we define in (31)). This upper bound is optimized by using the latent motifs that we obtain with the NDL algorithm using only injective homomorphisms. Therefore, the NDR algorithm with the option `InjHom` = `T` has a theoretical advantage over the NDR algorithm with the option `InjHom` = `F`.

As in the first line of (13), the problem of determining H_t in line 15 of Algorithm NDR is a standard convex problem, which one can solve by using Algorithm A1. There are two variants of the NDR algorithm. The variant is specified by the Boolean variable `denoising`. The NDR algorithm with `denoising` = `F` is identical to the network-reconstruction algorithm in [13], except for the thresholding step. The NDR algorithm with `denoising` = `T` is a new variant of NDR that we present in this paper for network denoising.

D.2. **Further discussion of the denoising variant of the NDR algorithm.** We now give a detailed discussion of Algorithm NDR with `denoising` = `T` for network-denoising applications. Recall that our network-denoising problem is to reconstruct a true network $\mathcal{G}_{\text{true}} = (V, A)$ from an observed network $\mathcal{G}_{\text{obs}} = (V, A')$. The scheme that we used to produce Figure 8 is the following:

- **D.1** Learn a network dictionary $W \in \mathbb{R}^{k^2 \times r}_{\geq 0}$ from an observed network $\mathcal{G}_{\text{obs}} = (V, A)$ using NDL (see Algorithm NDL).
- **D.2** Compute a reconstructed network $\mathcal{G}_{\text{recons}} = (V, A_{\text{recons}})$ using NDR (see Algorithm NDR) with inputs $\mathcal{G}_{\text{obs}} = (V, A)$ and W.
- **D.3** Fix an edge threshold $\theta \in [0, 1]$. If $\mathcal{G}_{\text{obs}}$ is $\mathcal{G}_{\text{true}}$ with additive (respectively, subtractive) noise, we classify each edge (respectively, nonedge) $\{x, y\}$ as 'positive' if and only if $A_{\text{recons}}(x, y) > \theta$.

As was discussed in [13, Remark 4], a limitation of using NDR with `denoising` = `F` for network denoising is that the meaning of successful classification for subtractive-noise cases is an 'inversion' of its meaning for additive-noise cases. Namely, NDR with `denoising` = `F` may assign large weights to false edges and small weights to true edges. We demonstrate this issue

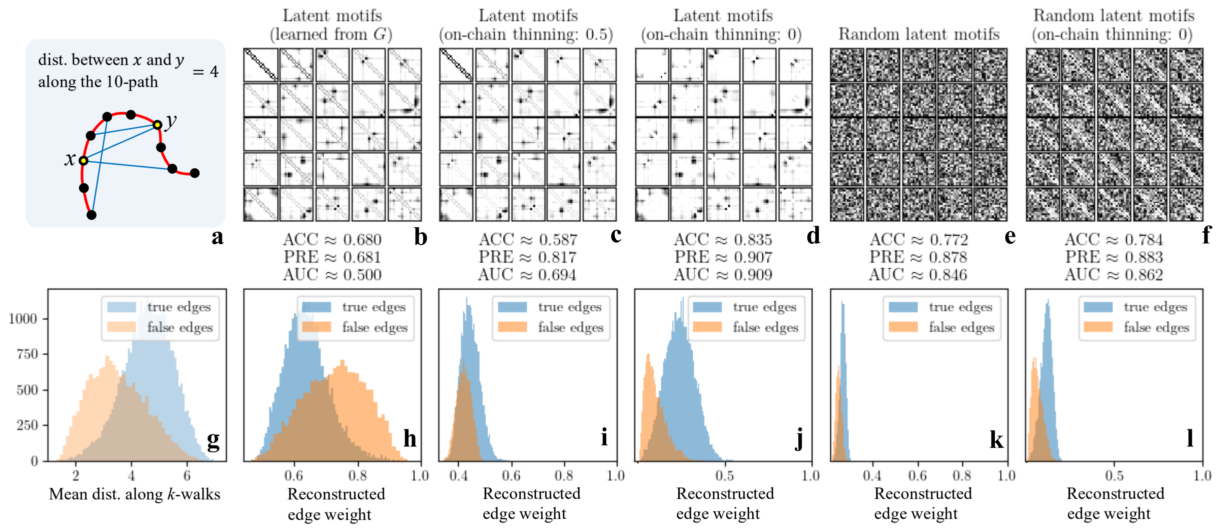

Supplementary Figure 4. Denoising Caltech corrupted by 50% additive noise of type ER. There are 16656 true (i.e., original) edges and 7854 false (i.e., added) edges to correctly classify. As we illustrate in (**a**), when a k-path connects two nodes x and y, we define the distance between x and y along the k-path to be the shortest-path distance between x and y. During reconstruction, we sample a sequence of k-paths of a network using a Markov-chain Monte Carlo (MCMC) algorithm (see Algorithm MP in the SI). Suppose that this sequence is $\mathbf{x}_1, \ldots, \mathbf{x}_T$. We compute the mean of the distances between x and y along the k-path $\mathbf{x}_t$ for all $t \in \{1, \ldots, T\}$ such that $\mathbf{x}_t$ connects x and y. In (**b**–**f**), we show the weighted adjacency matrices of five sets of 21-node latent motifs. We learn the 25 latent motifs in panel **b** from the corrupted network. We multiply the on-chain edge weights of these latent motifs by a thinning parameter $\xi = 0.5$ and $\xi = 0$ to obtain the matrices in panels **c** and **d**, respectively. We randomly choose the latent motifs in panel **e** by drawing each entry of its $k \times k$ weighted adjacency matrix independently and uniformly from $[0, 1]$. Setting the on-chain entries of these random latent motifs to 0 gives the matrices in panel **f**. In (**g**), we show histograms of the mean distances along the k-paths $\mathbf{x}_1, \ldots, \mathbf{x}_T$ between the two ends of true edges and the two ends of false edges. In (**h**–**l**), we show histograms of the edge weights for various reconstructions of the corrupted network using latent motifs in panels **b**–**f**. The classification accuracy (ACC) and precision (PRE) use the best threshold for truncating weighted edges in the reconstruction that we compute from a uniformly randomly chosen training set of edges (with 50% of the edges of the observed network). The AUC refers to the area under the ROC curve, which consists of points whose horizontal and vertical coordinates are the false-positive rates and true-positive rates, respectively.

in Supplementary Figure 4. As we can see in the histogram in Supplementary Figure 4**h**, when we denoise Caltech with +ER noise, the false edges have significantly larger weights than the true edges when we reconstruct the observed network using latent motifs that we learn from the corrupted network (see Supplementary Figure 4**b**). We thus obtain an AUC of at most 0.5 for our classification.

In the next two paragraphs, we discuss two other issues that occur when denoising additive noise for sparse real-world networks. (These issues do not arise in the image-denoising setting.) We also explain how NDR with `denoising` = `T` addresses these issues and allows us to use the unified classification scheme above for both additive and subtractive noise.

The first issue is that on-chain edges — regardless of whether they are true edges or false edges — tend to have large weights in the weighted reconstruction. Suppose that we obtain $\mathcal{G}_{\text{obs}}$ by adding false edges to a sparse unweighted network $\mathcal{G}_{\text{true}}$. The on-chain entries of the mesoscale patches $A_{\mathbf{x}}$ are always equal to 1. Therefore, the latent motifs that we learn from $\mathcal{G}_{\text{obs}}$ have the same on-chain entries. (See, e.g., Figure 4 of the main manuscript.) Consequently,

linearly approximating the mesoscale patches $A_{\mathbf{x}}$ of $\mathcal{G}_{\text{obs}}$ using the latent motifs that we learn from $\mathcal{G}_{\text{obs}}$ cannot distinguish between true and false on-chain entries. Furthermore, because $\mathcal{G}_{\text{obs}}$ is sparse, there are many fewer positive off-chain entries of $A_{\mathbf{x}}$ than on-chain entries of $A_{\mathbf{x}}$. Therefore, in a network reconstruction, linear approximations of $A_{\mathbf{x}}$ that use the latent motifs are likely to assign larger weights to on-chain entries of $A_{\mathbf{x}}$ than to off-chain entries. The resulting reconstruction of $\mathcal{G}_{\text{obs}}$ is thus similar to $\mathcal{G}_{\text{obs}}$, and it is very hard to detect false edges of $\mathcal{G}_{\text{obs}}$.

The second issue is that there is a bias in the numbers of edges in k-paths (i.e., in the 'distances' along those k-paths) that connect two nodes that are attached to a false edge. A uniformly random k-path, which we use throughout the denoising process, tends to use fewer edges to connect the nodes at the endpoints of false edges than to connect the nodes at the endpoints of true edges. In other words, if there is an edge between nodes x and y in an additively corrupted network and we uniformly randomly sample a k-path that uses both x and y, then the distance between between these two nodes along the sampled k-path tends to be small if the edge between x and y is false and tends to be large if it is true (see Supplementary Figure 4**g**). This indicates that there are not many ways to connect the nodes that are attached to the two ends of a false edge using a k-path that avoids that false edge. Consequently, of the edges between the nodes in a uniformly sampled k-path of an additively corrupted network, false edges are more likely to appear as on-chain edges than as off-chain edges. Therefore, as we see in Supplementary Figures 4**k**,**l**, we can reasonably successfully denoise the network `Caltech` with additive noise of type +ER using randomized latent motifs in which each we draw each entry of their associated weighted adjacency matrices independently and uniformly from $[0, 1]$. When we consider subtractive noise, an analogous observation holds for true nonedges and false nonedges.

One can address both issues by ignoring all on-chain entries for each sampled mesoscale patch $A_{\mathbf{x}}$ and for each latent motif in the network dictionary W that we use for denoising. To ignore the on-chain entries, we use the option `denoising` $=$ `T` in Algorithm NDR. By contrast, with the option `denoising` $=$ `F`, we keep all on-chain entries without any modification.

To discuss the effect of suppressing the on-chain edges with the option `denoising` $=$ `T` in more detail, we consider a modification of our network-reconstruction algorithm in which we thin out the on-chain edge weights of the latent motifs in W_{obs} prior to network reconstruction. Specifically, we multiply the weights of the on-chain edges of the latent motifs and in all sampled mesoscale patches by a scalar chain-edge 'thinning parameter' $\xi \in [0, 1]$. For instance, the latent motifs in Supplementary Figures 4**i**,**j** use $\xi = 0.5$ and $\xi = 0$, respectively. As we see in the histograms in Supplementary Figures 4**c**,**d**, the negative edges in the resulting reconstruction have significantly smaller weights than the positive edges. For example, with $\xi = 0$, we obtain an AUC of 0.91. Although the thinning parameter ξ can take any value in $[0, 1]$, in all of our experiments except the one in Supplementary Figure 4, we use only the extreme values $\xi = 0$ (i.e., `denoising` $=$ `T`) and $\xi = 1$ (i.e., `denoising` $=$ `F`). It seems to be unnecessary to use values of ξ in $(0, 1)$.

Appendix E. Experimental Details

E.1. **Figure 1.** The network in Figure 1**a** has 100 nodes and 216 edges. Of these edges, 164 are from the original network (see Figure 1**b**) and the remaining 51 are anomalous edges (see Figure 1**c**) that we generate using the $G(N, p)$ Erdős–Rényi (ER) network model with the same node set and edge probability $p = 0.01$. Specifically, we independently connect each pair of nonadjacent nodes by an anomalous edge with probability 0.01. We learn the $r = 25$ latent motifs in Figure 1**d** using the NDL algorithm (see Algorithm NDL) for a k-chain motif $F = ([k], A_F)$

at scale $k = 9$ for $T = 50$ iterations, $N = 100$ injective homomorphisms per iteration (so each iteration consists of sampling N injective homomorphisms and applying the online NMF update (13)), an L_1-regularizer with coefficient $\lambda = 1$, and the MCMC motif-sampling algorithm $\texttt{MCMC} = \texttt{PivotApprox}$.

To compute the weighted-network reconstruction of the observed network in Figure 1**e**, we use the NDR algorithm (see Algorithm NDR) with a k-chain motif $F = ([k], A_F)$ at scale $k = 9$ for $T = 10^5$ iterations, the 25 latent motifs in Figure 1**d**, an L_1-regularizer with coefficient $\lambda = 0$ (i.e., no regularization), the MCMC motif-sampling algorithm $\texttt{MCMC} = \texttt{PivotApprox}$, and $\texttt{denoising} = \texttt{F}$.

To evaluate our results, we split the data into training and test sets, with 50% of the 164 true edges and 50% of the 51 anomalous edges in each set. To maximize classification accuracy, we then determine an optimal threshold value θ to weight the edges of the reconstructed network. Specifically, we classify all edges in the test set as positive if their weight in the reconstructed network exceeded θ, and otherwise negative. In Figure 1**f**, we show all edges of the weighted reconstruction in Figure 1**e** whose weights are at most θ.

E.2. **Figure 2.** In Figure 2, we show six subgraphs that are induced by approximately uniformly random samples of k-paths with $k = 20$ (red edges) from the networks $\texttt{CALTECH}$, $\texttt{UCLA}$, $\texttt{ER}_1$, $\texttt{BA}_2$, $\texttt{WS}_2$, and $\texttt{SBM}_1$. To sample such k-paths, we use Algorithm MP with $\texttt{AcceptProb} = \texttt{Approximate}$. The red edges in each subgraph designate edges in the sampled k-paths (i.e., on-chain edges), and the blue edges designate edges (the off-chain edges) that connect nonadjacent nodes in sampled paths.

E.3. **Figure 3.** In Figure 3, we illustrate our low-rank network-reconstruction process using two sets of latent motifs. For both sets of latent motifs, we use 2500 k-paths (with $k = 7$) that we sample using the MCMC motif-sampling algorithm $\texttt{MCMC} = \texttt{PivotApprox}$ to compute the weighted reconstructions in panels **a4** and **b2**.

E.4. **Figure 4.** In Figure 4, we illustrate latent motifs that we learn from the networks UCLA and CALTECH and we compare these latent motifs to the elements of an image dictionary. The image in Figure 4**a** is from the collection DIE GRAPHIK ERNST LUDWIG KIRCHNERS BIS 1924, VON GUSTAV SCHIEFLER BAND I BIS 1916 (Accession Number 2007.141.9, Ernst Ludwig Kirchner, 1926). We use this image with permission from the National Gallery of Art in Washington, DC, USA.[2] We use a k-chain motif $F = ([k], A_F)$ and a scale $k = 21$ for $T = 100$ iterations, $N = 100$ injective homomorphisms per iteration, $r = 25$ latent motifs, an L_1-regularizer with coefficient $\lambda = 1$, and the MCMC motif-sampling algorithm $\texttt{MCMC} = \texttt{PivotApprox}$. The image dictionary for the artwork CYCLE in Figure 4 uses an algorithm that is similar to Algorithm NDL, except that we uniformly randomly sample image patches of size 21×21 instead of $k \times k$ mesoscale patches of a network.

E.5. **Figure 5.** To generate Figure 5, we first apply the NDL algorithm (see Algorithm NDL) to each network in the figure to learn $r = 25$ latent motifs for a k-chain motif $F = ([k], A_F)$ at scale $k = 21$ for $T = 100$ iterations, $N = 100$ injective homomorphisms per iteration, an L_1-regularizer with coefficient $\lambda = 1$, and the MCMC motif-sampling algorithm $\texttt{MCMC} = \texttt{PivotApprox}$. For each self-reconstruction $X \leftarrow X$ (see the caption of Figure 5), we apply the NDR algorithm (see Algorithm NDR) to a k-chain motif $F = ([k], A_F)$ at scale $k = 21$ for $T = \lfloor n \ln n \rfloor$ iterations (where n is the number of nodes of the network), $r = 25$ latent motifs, an L_1-regularizer with coefficient $\lambda = 0$ (i.e., no regularization), the MCMC motif-sampling

[2]See https://www.nga.gov/notices/open-access-policy.html for the open-access policy of the National Gallery of Art.

algorithm MCMC = PivotApprox, and denoising = F. For each cross-reconstruction $Y \leftarrow X$ (see the caption of Figure 5), we apply the NDR algorithm (see Algorithm NDR) to a k-chain motif with the corresponding network $F = ([k], A_F)$ at scale $k = 21$ for $T = (1+3\cdot\mathbb{1}(n < 1000))\lfloor n \ln n \rfloor$ iterations (where n is the number of nodes of the network), the edge threshold $\theta = 0.4$, an L_1-regularizer with coefficient $\lambda = 1$, the MCMC motif-sampling algorithm MCMC = PivotApprox, and denoising = InjHom = F. We use several choices of the number r of latent motifs; we indicate them in the caption of Figure 5.

In the main manuscript, we made several claims from the reconstruction accuracies in Figure 5 and the latent motifs in Figure 4 and Supplementary Figures 3, 8, and 10. We now justify these claims.

(1) The mesoscale structures of the network Caltech are rather different than those of Harvard, UCLA, and MIT at scale $k = 21$.

- In Figure 5**c**, we observe that the accuracy of the cross-reconstruction $X \leftarrow Y$ is consistently higher for $X \in$ {UCLA, Harvard, MIT} than for $X =$ Caltech for all values of r. For instance, for $r = 9$, we can reconstruct UCLA with more than 90% accuracy and we can reconstruct Harvard and MIT with more than 80% accuracy. However, the latent motifs that we learn from Caltech for $r = 9$ gives only about 80% accuracy for reconstructing UCLA and only about 70% accuracy for reconstructing MIT and UCLA. This indicates that the mesoscale structures of Caltech differ significantly from those of the other three universities' Facebook networks at scale $k = 21$. Indeed, from Figure 4 and Supplementary Figures 3 and 8, we see that the $r = 25$ latent motifs of Caltech at scale $k = 21$ have larger off-chain entries than those of UCLA, MIT, and Harvard.

(2) The mesoscale structures of Caltech at scale $k = 21$ are higher-dimensional than those of the other three universities' Facebook networks.

- Consider the cross-reconstructions Caltech $\leftarrow Y$ for $Y \in$ {UCLA, Harvard, MIT} in Figure 5**b**. With $r = 9$, the latent motifs that we learn from Caltech have accuracies as low as 64%. By contrast, the accuracies are 80% or higher for the self-reconstructions $X \leftarrow X$ for the Facebook networks of the other universities. In other words, $r = 9$ latent motifs at scale $k = 21$ do not approximate the mesoscale structures of Caltech as well as those of the other three universities' Facebook networks. This indicates that the dimension of the mesoscale structures of Caltech at scale $k = 21$ is larger than those of the other three universities' Facebook networks.

(3) The network $\mathtt{BA}_2$ is better than the networks $\mathtt{ER}_2$, $\mathtt{WS}_2$, and $\mathtt{SBM}_2$ at capturing the mesoscale structures of MIT, Harvard, and UCLA at scale $k = 21$. However, for $r \in \{9, 15, 25, 49\}$, the network $\mathtt{SBM}_2$ captures the mesoscale structures of Caltech better than all but one of the seven other networks in Figure 5**b**. (The only exception is Caltech itself.)

- From the reconstruction accuracies for $X \leftarrow Y$ in Figures 5**b**,**c**, where Y is one of the four synthetic networks ($\mathtt{ER}_2$, $\mathtt{WS}_2$, $\mathtt{BA}_2$, and $\mathtt{SBM}_2$), we observe that the two BA networks have higher accuracies then the networks from the ER and WS models for $Y \in$ {UCLA, Harvard, MIT}. This suggests that the mesoscale structures of UCLA, Harvard, and MIT are more similar in some respects to those of $\mathtt{BA}_2$ than to those of $\mathtt{ER}_2$, $\mathtt{WS}_2$, and $\mathtt{SBM}_2$. The latent motifs of $\mathtt{BA}_2$ in Supplementary Figures 3 and 10 at the scales $k \in \{11, 21\}$ have characteristics that we also observe in UCLA, Harvard, and MIT. (Specifically, they have nodes that are adjacent to many other nodes and off-chain entries that are much smaller — and hence in lighter shades — than the on-chain entries.) By contrast, in Supplementary Figure 11, we see that the latent motifs of $\mathtt{ER}_2$ have sparse but seemingly randomly distributed off-chain connections and that the latent motifs for $\mathtt{WS}_2$ have strongly interconnected communities of about 10 nodes.

These patterns differ from the ones that we observe in the latent motifs for UCLA, MIT, and Harvard (see Supplementary Figure 8). For the claim about $\texttt{SBM}_2$, observe that the cross-reconstruction accuracy of Caltech $\leftarrow$ $\texttt{SBM}_2$ in Figure 5 is larger than those of all other reconstructions Caltech $\leftarrow Y$ except Y = Caltech. Additionally, the theoretical lower bound of the Jaccard reconstruction accuracy for Caltech $\leftarrow$ $\texttt{SBM}_2$ in Figure 7**a** is larger than the corresponding lower bounds for all other Caltech $\leftarrow Y$ except Y = Caltech.

(4) If we uniformly randomly sample a path with $k = 21$ nodes, we are more likely to obtain communities with 10 or more nodes in an associated induced subgraph for Caltech than for UCLA, Harvard, and MIT.

- This observation manifests directly in the box plots in Supplementary Figure 2 for the community sizes in the latent motifs and subgraphs that are induced by k-paths. We can also indirectly justify this observation. From the reconstruction accuracies for $X \leftarrow Y$ in Figures 5**b**–**e**, where Y is one of the four synthetic networks ($\texttt{ER}_2$, $\texttt{WS}_2$, $\texttt{BA}_2$, and $\texttt{SBM}_2$), we observe that $\texttt{WS}_2$ is better than the BA and ER networks at reconstructing Caltech but that it is one of the worst-performing networks for reconstructing the Facebook networks of the other three universities. In other words, the nonnegative linear combinations of the latent motifs of $\texttt{WS}_2$ better approximate the mesoscale patches of Caltech than the mesoscale patches of UCLA, Harvard, and MIT. Recall that most latent motifs of $\texttt{WS}_2$ at scale $k = 21$ have communities with 10 or more nodes. It seems that this community structure is more likely to occur in subgraphs that are induced by uniformly random samples of k-paths in Caltech with $k = 21$ nodes than from such samples in UCLA, Harvard, or MIT.

E.6. **Figure 6.** In Figure 6, we compare the degree distributions and the mean local clustering coefficients of the original and the reconstructed networks that use r latent motifs at scale $k = 21$. We conduct this experiment for the five networks in Figure 5**a**. In Figure 6**a**, we use the unweighted reconstructed networks for Caltech with $r \in \{9, 16, 25, 64\}$ latent motifs that we used to compute the self-reconstruction accuracies in 5**b**. In Figures 6**b**–**e**, we use the unweighted reconstructed networks for Coronavirus, H. sapiens, SNAP FB, and arXiv with $r = 25$ latent motifs that we used to compute the self-reconstruction accuracies in Figures 5**b**–**e**.

E.7. **Figure 7.** For each experiment $X \leftarrow Y$ in Figures 7**a**–**d**, we plot

$$1 - \frac{\mathbb{E}_{\mathbf{x}\sim\pi}[\|A_{\mathbf{x}} - \hat{A}_{\mathbf{x};W}\|_1]}{2(k-1)}\,, \tag{15}$$

where $k = 21$ and W is the network dictionary of $r = 25$ latent motifs in network Y that we determine using our NDL algorithm (see Algorithm NDL) for a k-chain motif $F = ([k], A_F)$ for $T = 100$ iterations, $N = 100$ injective homomorphisms per iteration, an L_1-regularizer with coefficient $\lambda = 1$, and the MCMC motif-sampling algorithm $\texttt{MCMC} = \texttt{PivotApprox}$. The distribution π is the stationary distribution $\hat{\pi}_{F\hookrightarrow\mathcal{G}}$ (see (17)) of the injective MCMC motif-sampling algorithm (see Algorithm IM and Proposition F.9). For each mesoscale patch $A_{\mathbf{x}} \in \mathbb{R}^{k\times k}$, we compute the linear approximation $\hat{A}_{\mathbf{x};W}$ (see line 16 of Algorithm NDR). We approximate the expectation in the numerator of (15) using a Monte Carlo method. Specifically, from the convergence result in Proposition F.3, we have

$$\mathbb{E}_{\mathbf{x}\sim\pi}\left[\|A_{\mathbf{x}} - \hat{A}_{\mathbf{x};W}\|_1\right] = \lim_{N\to\infty}\frac{1}{N}\sum_{t=1}^{N}\|A_{\mathbf{x}_t} - \hat{A}_{\mathbf{x}_t;W}\|_1\,, \tag{16}$$

where $(\mathbf{x}_t)_{t\geq 0}$ is a sequence of injective homomorphisms $F \hookrightarrow \mathcal{G}$ that we sample using the injective MCMC motif-sampling algorithm (see Algorithm IM). We use the finite sample mean $\frac{1}{N}\sum_{t=1}^{N}\|A_{\mathbf{x}_t} - \hat{A}_{\mathbf{x}_t;W}\|_1$ with $N = 10^4$ as a proxy of the expectation in the left-hand side of (16).

E.8. **Figure 8.** To generate Figure 8, we first apply the NDL algorithm (see Algorithm NDL) to each corrupted network in the figure to learn $r = 25$ latent motifs for a k-chain motif $F = ([k], A_F)$ at scale $k = 21$ for $T = 400$ iterations, $N = 1000$ homomorphisms per iteration, an L_1-regularizer with coefficient $\lambda = 1$, and the MCMC motif-sampling algorithm `MCMC` = `PivotApprox`. The NDR algorithm (see Algorithm NDR) that we use to generate the results in Figure 8 uses $r \in \{2, 25\}$ latent motifs for a k-chain motif with the corresponding network $F = ([k], A_F)$ at scale $k = 21$ for $T = 4 \times 10^5$ iterations for `H. sapiens` and $T = 2 \times 10^5$ iterations for all other networks, an L_1-regularizer with coefficient $\lambda = 1$, the MCMC motif-sampling algorithm `MCMC` = `PivotApprox` with `InjHom` = `F` and `denoising` $\in$ {`T`, `F`}.

For Figure 8, we do not conduct the denoising experiment for `Coronavirus PPI` with -50% noise because the resulting network (with 1536 nodes and 1232 edges) cannot be connected. (To be connected, its spanning trees need to have 1535 edges.)

We implement several existing network-denoising methods — the Jaccard index, preferential attachment, the Adamic–Adar index, spectral embedding, DeepWalk, and node2vec — and compare the performance of our method to those of these existing approaches. Let $\mathcal{G} = (V, A)$ be an original network and let $\mathcal{G}' = (V, A)$ be the associated corrupted network. For our experiments in Figure 8, both of these networks are undirected and unweighted. In all cases, we obtain a network $\hat{\mathcal{G}} = (V, \hat{A})$ from $\mathcal{G}' = (V, A)$ without using $\mathcal{G}$. For each $x, y \in V$, we compute the 'confidence score' $\hat{A}(x, y)$ that the node pair (x, y) is an edge $\{x, y\}$ in the original network $\mathcal{G}$. Let $N(x)$ denote the set of neighbors of node x in $\mathcal{G}'$. (This set includes x itself when there is a self-edge at x.) For the Jaccard index, preferential attachment, and the Adamic–Adar index, we compute the confidence score $\hat{A}(x, y)$ by calculating $|N(x) \cap N(y)|/|N(x) \cup N(y)|$, $|N(x)| \cdot |N(y)|$, and $\sum_{z \in N(x)\cap N(y)} 1/\ln|N(z)|$, respectively. The Adamic–Adar index is not defined for nodes with self-edges, and the networks `arXiv`, `Coronavirus`, and `H. sapiens` have self-edges. Therefore, we do not include self-edges in the network-denoising experiments in Figure 8 (but we do not remove self-edges for any other experiment).

For spectral embedding, DeepWalk, and node2vec, we first obtain a 128-dimensional vector representation of the nodes of a network; this is a so-called 'node embedding' of the network. We then use this node embedding to obtain vector representations of the edges using binary operations. (We use the Hadamard product; see [15] for details.) We then use logistic regression (but one can alternatively employ some other algorithm for binary classification) to attempt to detect the false edges. Spectral Clustering uses the top 128 eigenvectors of the combinatorial Laplacian matrix of $\mathcal{G}'$ to learn vector embeddings of the nodes. (We obtain the combinatorial Laplacian matrix of $\mathcal{G}'$ by subtracting its adjacency matrix from the diagonal matrix of node degrees.) See [68] for details. DeepWalk and node2vec first sample sequences of random walks on $\mathcal{G}'$ and then apply the popular word-embedding algorithm word2vec [69]. In DeepWalk, each random walk is a standard random walk on the network $\mathcal{G}'$. For node2vec, we use the 16 choices of the 'return parameter' p and the 'in–out parameter' q with $(p, q) \in \{0.25, 0.5, 1, 2\}$. In addition to the random walk-sampling in these two methods, we use the following common choices (which are the same ones that were made in [15]) for both methods. Using each node of $\mathcal{G}'$ as a starting point, we independently sample 10 random walks of 80 steps, a context window size of 10, one stochastic-gradient epoch (i.e., 1 pass through the training data), and 8 workers (i.e., 8 parallel threads for training). See [15] for details.

For all approaches, we first split the data into a 25/25/50 split of training/validation/test sets. We then construct the network $\hat{\mathcal{G}} = (V, \hat{A})$. By varying the threshold parameter θ, we construct a receiver-operating characteristic (ROC) curve that consists of points whose horizontal and vertical coordinates are the false-positive rates and true-positive rates, respectively. For denoising noise of type −ER (respectively, +ER and +WS), we also infer an optimal value of θ for a 25% validation set of nonedges (respectively, edges) of $\mathcal{G}'$ with known labels and then use that value of θ to compute classification measures (such as accuracy and precision) for the test set.

E.9. **Figure 9.** In Figure 9, we illustrate cross-reconstruction experiments for images using mesoscale patches of size 21×21. The image that we seek to reconstruct is `Woman with a Parasol - Madame Monet and Her Son` (Claude Monet, 1875), which we show in Figure 9**a**. The image in Figure 9**b** is a reconstruction of this image using the dictionary with 25 basis images of size 21×21 pixels in Figure 9**c**, where we choose the color of each pixel uniformly at random from all possible colors (which we represent as vectors in $[0, 256]^3$ for red–green–blue (RGB) weights). The image in Figure 9**d** is a reconstruction of the image in Figure 9**a** using the dictionary with 25 basis images of size 21×21 pixels in Figure 9**e**. We learn this basis from the image in Figure 9**f** using NMF [25]. The image in Figure 9**f** is from the collection `Die Graphik Ernst Ludwig Kirchners bis 1924, von Gustav Schiefler Band I bis 1916` (Accession Number 2007.141.9, Ernst Ludwig Kirchner, 1926). We use the images in Figures 9**a**,**f** with permission from the National Gallery of Art in Washington, DC, USA. Their open-access policy is available at `https://www.nga.gov/notices/open-access-policy.html`.

E.10. **Figure 10.** In Figure 10, we compare 10^4 subgraphs (we show 33 of them) that are induced by approximately uniformly random (**a**) k-paths and (**b**) k-walks for the network `Coronavirus PPI` with $k = 10$. To sample k-walks, we use an MCMC motif-sampling algorithm (see Algorithm MP with `AcceptProb` = `Approximate`). To sample k-paths, we use the injective MCMC motif-sampling algorithm (see Algorithm IM).

To obtain the latent motifs in Figure 10**c**, we use the NDL Algorithm (see Algorithm NDL) with `MCMC` = `PivotApprox`. To obtain the latent motifs in Figure 10**d**, we instead use the NDL algorithm of Lyu et al. [13]. This algorithm is equivalent to our NDL algorithm (see Algorithm NDL) if we use all homomorphisms from the k-chain motif F to the network $\mathcal{G}$, rather than only the injective ones as in Algorithm NDL. For the experiments in Figures 10**c**,**d**, we use a k-chain motif $F = ([k], A_F)$ with $k = 21$, $T = 100$ iterations, $N = 100$ injective homomorphisms per iteration (so we sample a total of 10^4 injective homomorphisms), and an L_1-regularizer with coefficient $\lambda = 1$.

E.11. **Supplementary Figure 1.** In Supplementary Figure 1, we give a schematic illustration of the NDL algorithm (see Algorithm NDL).

E.12. **Supplementary Figure 3.** In this figure, we show latent motifs of the examined networks (which we described in the Methods section of the main manuscript) using Algorithm NDL with various parameter choices. In each column of this figure, we use a k-chain motif $F = ([k], A_F)$ for $T = 100$ iterations, $N = 100$ injective homomorphisms per iteration (so we sample a total of 10^4 injective homomorphisms), an L_1-regularizer with coefficient $\lambda = 1$, and the injective MCMC motif-sampling algorithm `MCMC` = `PivotApprox`. We specify the number r of latent motifs and the scale k in the caption of Supplementary Figure 3.

E.13. **Supplementary Figure 2.** In Supplementary Figure 2, we compare box plots of the community sizes of 10000 sampled subgraphs that are induced by approximately uniformly random paths of $k = 21$ nodes (in red) to the corresponding box plots of $r = 25$ latent motifs

of $k = 21$ nodes for various real-world and synthetic networks. To sample these paths, we use Algorithm MP with $\texttt{AcceptProb} = \texttt{Approximate}$. (See the Methods section of the main manuscript.) We determine latent motifs using NDL (see Algorithm NDL) with a k-chain motif $F = ([k], A_F)$ for $T = 100$ iterations, $N = 100$ injective homomorphisms per iteration (so we sample a total of 10^4 injective homomorphisms), an L_1-regularizer with coefficient $\lambda = 1$, and the injective MCMC motif-sampling algorithm $\texttt{MCMC} = \texttt{PivotApprox}$. We determine the communities of the subgraphs and the latent motifs using the locally-greedy Louvain algorithm for modularity maximization [66].

We perform statistical testing to compare the community sizes of the subgraphs ('sample 1') and the latent motifs ('sample 2'). We select a uniformly random subset of sample 1 to match the size of sample 2 and perform Mood's median test [67] to obtain a p-value. We repeat this experiment 100 times for each network and report the mean value of the resulting 100 p-values in parentheses in Supplementary Figure 2. A sufficiently small p-value indicates that there is statistically significant evidence that the two samples come from populations with distinct medians.

E.14. **Supplementary Figure 4.** In Supplementary Figure 4, we show histograms of various statistics for true and false edges when we denoise the network CALTECH after corrupting it with 50% additive noise of type +ER.

We say that a k-walk $\mathbf{x}_t$ 'visits' an edge between distinct nodes x and y if there exist indices a and b such that $1 \leq a < b \leq k$ with $\mathbf{x}_t(a) = x$ and $\mathbf{x}_t(b) = y$. We define the 'distance' between nodes x and y along the k-walk $\mathbf{x}_t$ that visits the edge between them by the minimum value of $|i - j|$, where i and j are integers in $\{1, \ldots, k\}$ such that $\mathbf{x}_t(i) = x$ and $\mathbf{x}_t(j) = y$. (See the illustration in Supplementary Figure 4**a**.) We then compute the mean of such distances between x and y for all $t \in \{1, \ldots, T\}$ for which $\mathbf{x}_t$ visits the edge between x and y. In Supplementary Figure 4**g**, we show the histogram of the mean distances along the k-walks $\mathbf{x}_1, \ldots, \mathbf{x}_T$ between the two ends of true edges and the two ends of false edges.

We compute the $r = 25$ latent motifs in Supplementary Figure 4**h** using NDL (see Algorithm NDL) with a k-chain motif with the corresponding network $F = ([k], A_F)$ for $T = 100$ iterations, $N = 100$ injective homomorphisms per iteration (so we sample a total of 10^4 injective homomorphisms), an L_1-regularizer with coefficient $\lambda = 1$, and the injective MCMC motif-sampling algorithm $\texttt{MCMC} = \texttt{PivotApprox}$.

For our reconstructions of the corrupted network CALTECH in Supplementary Figures 4**b**–**f**, which use the corresponding latent motifs in Supplementary Figures 4**h**–**l**, respectively, we apply the NDR algorithm (see Algorithm NDR) to a k-chain motif with the corresponding network $F = ([k], A_F)$ at scale $k = 21$ for $T = 2 \times 10^5$ iterations, $N = 100$ homomorphisms per iteration, $r = 25$ latent motifs, an L_1-regularizer with coefficient $\lambda = 0$ (i.e., no regularization), and the MCMC motif-sampling algorithm $\texttt{MCMC} = \texttt{PivotApprox}$ with $\texttt{InjHom} = \texttt{F}$ and $\texttt{denoising} = \texttt{F}$.

E.15. **Supplementary Figures 5, 6, and 7.** One summarizes the result of a binary classification using combinations of four quantities: TP (true positives), which is the number of positives that are classified as positive; TN (true negatives), which is the number of negatives that are classified as negative; FP (false positives), which is the number of positives that are classified as negative; and FN (false negatives), which is the number of negatives that are classified as positive. The total number of examples is the sum of these four quantities. Accuracy is $\frac{\text{TP+TN}}{\text{TP+TN+FP+FN}}$, precision is $\frac{\text{TP}}{\text{TP+FP}}$, recall is $\frac{\text{TP}}{\text{TP+FN}}$, negative predictive value (NPV) is $\frac{\text{TN}}{\text{TN+FN}}$, and specificity is $\frac{\text{TN}}{\text{TN+FP}}$. By relabeling positives as negatives and negatives as positives, precision becomes NPV and recall becomes specificity. See [70] for a discussion of NPV and specificity.

In Supplementary Figure 5, we show the accuracy, precision, and recall scores of the network-denoising experiments in Figure 8 at a fixed threshold θ. In Supplementary Figure 6, we show the dependence of the precision and recall scores of the network-denoising experiments in Figure 8 on the threshold θ. For denoising +WS and +ER noise, our approach yields larger AUCs for the precision–recall curves than all of the other examined network-denoising methods. Our approach performs competitively for denoising −ER noise, except for the network `H. Sapiens`. In Supplementary Figure 7, we show the dependence of the NPV and specificity scores of the network-denoising experiments in Figure 8 on the threshold θ. Our approach yields larger AUCs for the NPV–specificity curves than all of the other examined methods for denoising +WS and +ER noise, except for +ER for the network `Caltech`. For denoising −ER noise, our approach does not seem to be particularly effective at detecting unobserved edges. It is outperformed by other methods for `SNAP FB` (by all of them except PREFERENTIAL ATTACHMENT), `arXiv` (by all of them except PREFERENTIAL ATTACHMENT and SPECTRAL EMBEDDING), and `H. Sapiens` (by all of them except PREFERENTIAL ATTACHMENT and SPECTRAL EMBEDDING).

E.16. **Supplementary Figures 8, 9, 10, 11, 12, 13, 14, and 15.** In these figures, we show latent motifs of the examined networks (which we described in the Methods section of the main manuscript) using Algorithm NDL with various parameter choices. For each network, we use a k-chain motif with the corresponding network $F = ([k], A_F)$ for $T = 100$ iterations, $N = 100$ injective homomorphisms per iteration (so we sample a total of 10^4 injective homomorphisms), an L_1-regularizer with coefficient $\lambda = 1$, and the injective MCMC motif-sampling algorithm $\texttt{MCMC} = \texttt{PivotApprox}$. We specify the number r of latent motifs and the scale k in the caption of each figure.

Appendix F. Convergence Analysis

In this section, we give rigorous convergence guarantees for our main algorithms for NDL (see Theorems F.4 and F.7) and NDR (see Theorems F.10 and F.14). All of these results are novel. Lyu et al. [13] proposed a network-reconstruction algorithm that corresponds to our NDR algorithm NDR with the choice $\texttt{denoising} = \texttt{InjHom} = \texttt{F}$. They did not do any theoretical analysis of this network-reconstruction algorithm. Our most significant theoretical contribution is our guarantees about the NDR algorithm (see Algorithm NDR). Specifically, in Theorems F.10 and F.14, we establish convergence, exact formulas, and error bounds of the reconstructed networks for all four choices $(\texttt{denoising}, \texttt{InjHom}) \in \{\texttt{T}, \texttt{F}\}^2$ for both non-bipartite and bipartite networks. The most interesting aspect of these results is our bound for the Jaccard reconstruction error in terms of the mesoscale approximation error divided by the number of nodes of the subgraphs at that mesoscale (see Theorem F.10**(iii)**). Roughly speaking, this result guarantees that one can accurately reconstruct a network if one has a dictionary of latent motifs that can accurately approximate the subgraphs in the network at a fixed mesoscale. We illustrate this result with supporting experiments in Figure 7 of the main manuscript. A crucial feature of our proof of Theorem F.10**(iii)** is our use of an explicit formula for the weight matrix of the limiting reconstructed network as the number of iterations that we use for network reconstruction tends to infinity.

In [13, Corollary 6.1], Lyu et al. presented a convergence guarantee for the original NDL algorithm in [13] for non-bipartite networks $\mathcal{G}$. The key difference between our NDL algorithm (see Algorithm NDL) and the NDL algorithm in [13] is that we employ k-path motif sampling but they employ k-walk motif sampling. This results in different objective functions to minimize. (See (7) for our objective function.) Therefore, [13, Corollary 6.1] does not apply to our NDL algorithm (see Algorithm NDL). In Theorem, F.4, we establish a convergence result for our NDL algorithm. A key step in the proof of this result is guaranteeing convergence of the

injective MCMC motif-sampling algorithm (see Algorithm IM) for non-bipartite networks (see Proposition F.3). In Theorem F.7, we extend the convergence results for our NDL algorithm to bipartite networks. Convergence for bipartite networks was not established in [13] even for the original NDL algorithm. The key technical difficulty for bipartite networks is that Markov chains that are generated by the MCMC motif-sampling algorithms in Algorithms MP (for either option for `AcceptProb`) and MG are not irreducible, so one cannot apply the main convergence results for online NMF in [13, Thm. 1]. Our proof of Theorem F.7 uses a careful coupling argument between two reducible classes of Markov chains.

Let $F = ([k], A_F)$ be the network corresponding to a k-chain motif, and let $G = (V, A)$ be a network. Let $\Omega \subseteq V^{[k]}$ denote the set of all homomorphisms (which do not have to be injective) $\mathbf{x} : F \to \mathcal{G}$. Algorithm NDL generates three stochastic sequences. The first one is the sequence $(\mathbf{x}_t)_{t\geq 0}$ of injective homomorphisms $F \hookrightarrow \mathcal{G}$ that we obtain from the injective MCMC motif-sampling algorithm (see Algorithm IM). The second one is the sequence $(X_t)_{t\geq 0}$ of $k^2 \times N$ data matrices whose columns encode N mesoscale patches of $\mathcal{G}$. More precisely, for each $\mathbf{y}_1, \dots, \mathbf{y}_n \in \Omega$, we write $\Psi(\mathbf{y}_1, \dots, \mathbf{y}_n) \in \mathbb{R}^{k^2\times N}_{\geq 0}$ for the $k^2 \times N$ matrix whose i^{th} column is the vectorization (using Algorithm A4) of the corresponding $k \times k$ mesoscale patch $A_{\mathbf{y}_i}$ of $\mathcal{G}$ (see (5)). For each $\mathbf{y}_0 \in \Omega$, define

$$X^{(N)}(\mathbf{y}_0) := \Psi(\mathbf{y}_1, \dots, \mathbf{y}_N) \in \mathbb{R}^{k^2\times N}_{\geq 0} ,$$

where we generate $\mathbf{y}_1, \dots, \mathbf{y}_N$ iteratively using the injective MCMC motif-sampling algorithm (see Algorithm IM), which we initialize with the homomorphism $\mathbf{y}_0 : F \to \mathcal{G}$. It then follows that $X_t = X^{(N)}(\mathbf{x}_{Nt})$ for each $t \geq 1$, where $\mathbf{x}_{Nt}$ is the injective homomorphism $F \hookrightarrow \mathcal{G}$ that we obtain after Nt applications of Algorithm IM. For the third (and final) sequence that we generate using Algorithm NDL, let $(W_t)_{t\geq 0}$ denote the sequence of dictionary matrices, where we define each $W_t = W_t(\mathbf{x}_0)$ via (13) with an initial homomorphism $\mathbf{x}_0 : F \to \mathcal{G}$ that we sample using Algorithm A3.

F.1. **Convergence of the MCMC algorithms.** In this subsection, we establish the convergence properties of the MCMC algorithms (see Algorithms MP and MG) for sampling a k-walk according to the target distribution $\pi_{F\to\mathcal{G}}$ (see (2)). Recall that this distribution is the uniform distribution on the set of all homomorphisms $F \to \mathcal{G}$ when the motif F and the network $\mathcal{G}$ are both symmetric and unweighted (see (4)).

Proposition F.1. *Fix a network $\mathcal{G} = (V, A)$ and a k-chain motif $F = ([k], A_F)$. Let $(\mathbf{x}_t)_{t\geq 0}$ denote a sequence of homomorphisms $\mathbf{x}_t : F \to \mathcal{G}$ that we generate using the exact pivot chain (in which we use Algorithm MP with* `AcceptProb` $=$ `Exact`*) or the Glauber chain (in which we use Algorithm MG). Suppose that*

(a) *The weight matrix A is 'bidirectional' (i.e., $A(x, y) > 0$ implies that $A(y, x) > 0$ for all $x, y \in V$) and that the binary network $(V, \mathbb{1}(A > 0))$ is connected and non-bipartite.*

It then follows that $(\mathbf{x}_t)_{t\geq 0}$ is an irreducible and aperiodic Markov chain with the unique stationary distribution $\pi_{F\to\mathcal{G}}$ that we defined in (2).

Proof. This proposition was proved rigorously in [21, Thms. 5.7 and 5.8]. In the present paper, we sketch the proof for the exact pivot chain to illustrate the main idea behind the acceptance probability in (9). The trajectory of the first node $\mathbf{x}_t(1)$ of the homomorphism $\mathbf{x}$ gives a standard random walk on the network $\mathcal{G}$ that is modified by the Metropolis–Hastings algorithm (see, e.g., [61, Sec. 3.2]) so that it has the following marginal distribution as its unique stationary distribution:

$$\pi^{(1)}(x_1) = \frac{\sum_{x_2,\dots,x_k\in[n]} \prod_{i=2}^{k} A(x_{i-1}, x_i)}{\mathtt{Z}} ,$$

where the denominator $\mathtt{Z} = \mathtt{Z}(F, \mathcal{G})$ is the normalization constant that we call the 'homomorphism density' of F in $\mathcal{G}$ (see [55]) and the numerator is proportional to the probability of sampling $x_2, \dots, x_k \in [n]$ for $\mathbf{x}_t(2), \dots, \mathbf{x}_t(k)$. Fix a homomorphism $\mathbf{x} : F \to \mathcal{G}$ with $\mathbf{x}(i) = x_i$ for $i \in \{1, \dots, k\}$. We then obtain

$$\begin{aligned}\mathbb{P}(\mathbf{x}_t(1) = x_1, \dots, \mathbf{x}_t(k) = x_k) &= \mathbb{P}\left(\mathbf{x}_t(1) = x_1\right) \mathbb{P}(\mathbf{x}_t(2) = x_2, \dots, \mathbf{x}_t(k) = x_k \,|\, \mathbf{x}_t(1) = x_1) \\ &\approx \pi^{(1)}(x_1) \frac{\prod_{i=2}^k A(x_{i-1}, x_i)}{\sum_{y_2,\dots,y_k \in [n]} \prod_{i=2}^k A(y_{i-1}, y_i)} \\ &= \frac{\prod_{i=2}^k A(x_{i-1}, x_i)}{\mathtt{Z}} = \pi_{F \to \mathcal{G}}(\mathbf{x})\,,\end{aligned}$$

where the approximation in the second line above becomes exact as $t \to \infty$. □

We now prove Proposition F.2, which guarantees the convergence of the approximate pivot chain and gives an explicit formula for its unique stationary distribution.

Proposition F.2. *Fix a network $\mathcal{G} = (V, A)$ and a k-chain motif $F = ([k], A_F)$. Let $(\mathbf{x}_t)_{t \geq 0}$ denote a sequence of homomorphisms $\mathbf{x}_t : F \to \mathcal{G}$ that we generate using the approximate pivot chain (in which we use Algorithm MP with* `AcceptProb` = `Approximate`*). Suppose that*

(a) *The weight matrix A is bidirectional (i.e., $A(x, y) > 0$ implies that $A(y, x) > 0$ for all $x, y \in V$) and that the undirected and unweighted graph $(V, \mathbb{1}(A > 0))$ is connected and non-bipartite.*

It then follows that $(\mathbf{x}_t)_{t \geq 0}$ is an irreducible and aperiodic Markov chain with the unique stationary distribution $\hat{\pi}_{F \to \mathcal{G}}$ that we defined in (12).

Proof. We follow the proof of [21, Thm. 5.8]. Let $P : V^2 \to [0, 1]$ be a matrix with entries

$$P(x, y) := \frac{A(x, y)}{\sum_{c \in V} A(a, c)}\,, \quad x, y \in V\,.$$

The matrix P is the transition matrix of the standard random walk on the network $\mathcal{G}$. By hypothesis (a), P is irreducible and aperiodic. Using a result in [61, Ch. 9], it has the unique stationary distribution

$$\pi^{(1)}(v) := \sum_{c \in V} A(v, c) / \sum_{c, c' \in V} A(c, c')\,.$$

The approximate pivot chain generates a move $\mathbf{x}_t(1) \mapsto \mathbf{x}_{t+1}(1)$ of the pivot according to the distribution $P(\mathbf{x}_t(1), \cdot)$. We accept this move independently of everything else with the approximate acceptance probability α in (9). If we always accept each move of the pivot, then the pivot performs a random walk on $\mathcal{G}$ with the unique stationary distribution $\pi^{(1)}$. We compute the acceptance probability α using the Metropolis–Hastings algorithm (see [61, Sec. 3.3]), and we thereby modify the stationary distribution of the pivot from $\pi^{(1)}$ to the uniform distribution on V. (See the discussion in [21, Sec. 5].) Therefore, $(\mathbf{x}_t(1))_{t \geq 0}$ is an irreducible and aperiodic Markov chain on V that has the uniform distribution as its unique stationary distribution. Because we sample the locations $\mathbf{x}_{t+1}(i) \in V$ of the subsequent nodes $i \in \{2, 3, \dots, k\}$ independently, conditional on the location $\mathbf{x}_{t+1}(1)$ of the pivot, it follows that the approximate pivot chain $(\mathbf{x}_t)_{t \geq 0}$ is also an irreducible and aperiodic Markov chain with a unique stationary distribution, which we denote by $\hat{\pi}_{F \to \mathcal{G}}$.

To determine the stationary distribution $\hat{\pi}_{F \to \mathcal{G}}$, we decompose $\mathbf{x}_t$ into the return times of the pivot $\mathbf{x}_t(1)$ to a fixed node $x_1 \in V$ in $\mathcal{G}$. Specifically, let $\tau(j)$ be the jth return time of $\mathbf{x}_t(1)$

to x_1. By the independence of sampling $\mathbf{x}_t$ for $t \in \{2, \ldots, k\}$, the strong law of large numbers yields

$$\lim_{M\to\infty} \frac{1}{M} \sum_{j=1}^{M} \mathbb{1}(\mathbf{x}_{\tau(j)}(2) = x_2, \ldots, \mathbf{x}_{\tau(j)}(k) = x_k)$$
$$= \frac{\prod_{i=2}^{k} A(x_{i-1}, x_i)}{\sum_{y_2,\ldots,y_k \in V} \prod_{i=2}^{k} A(x_1, y_2)A(y_2, y_3)\cdots A(y_{k-1}, y_k)} .$$

For each fixed homomorphism $\mathbf{x} : F \to \mathcal{G}$, which maps $i \mapsto x_i$, we use the Markov-chain ergodic theorem (see, e.g., [71, Theorem 6.2.1 and Example 6.2.4] or [72, Theorem 17.1.7]) to obtain

$$\begin{aligned}
\hat{\pi}_{F\to\mathcal{G}}(\mathbf{x}) &= \lim_{N\to\infty} \frac{1}{N} \sum_{t=0}^{N} \mathbb{1}(\mathbf{x}_t = \mathbf{x}) \\
&= \lim_{N\to\infty} \frac{\sum_{t=0}^{N} \mathbb{1}(\mathbf{x}_t = \mathbf{x})}{\sum_{t=0}^{N} \mathbb{1}(\mathbf{x}_t(1) = x_1)} \, \frac{\sum_{t=0}^{N} \mathbb{1}(\mathbf{x}_t(1) = x_1)}{N} \\
&= \mathbb{P}\left(\mathbf{x}_t(2) = x_2, \ldots, \mathbf{x}_t(k) = x_k \,\middle|\, \mathbf{x}_t(1) = x_1\right) \pi^{(1)}(x_1) \\
&= \frac{\prod_{i=1}^{k} A(x_{i-1}, x_i)}{\sum_{y_2,\ldots,y_k \in V} \prod_{i=2}^{k} A(x_1, y_2)A(y_2, y_3)\cdots A(y_{k-1}, y_k)} \, \frac{1}{|V|} .
\end{aligned}$$

This proves the assertion. □

Finally, we establish the following asymptotic convergence result for the injective MCMC motif-sampling algorithm in Algorithm IM.

Proposition F.3 (Convergence of injective motif sampling)**.** *Fix a network $\mathcal{G} = (V, A)$ and a k-chain motif $F = ([k], A_F)$. Suppose that $\mathcal{G}$ has at least one k-path. Let $(\mathbf{x}_t)_{t\geq 0}$ denote a sequence of injective homomorphisms $\mathbf{x}_t : F \hookrightarrow \mathcal{G}$ that we generate using Algorithm IM. Suppose that*

(a) *The weight matrix A is bidirectional (i.e., $A(x, y) > 0$ implies that $A(y, x) > 0$ for all $x, y \in V$) and that the undirected and unweighted graph $(V, \mathbb{1}(A > 0))$ is connected and non-bipartite.*

The following statements hold:

(i) *If we use Algorithm MG or Algorithm MP with* `AcceptProb` = `Exact` *in Algorithm IM, then $(\mathbf{x}_t)_{t\geq 0}$ is an irreducible and aperiodic Markov chain with the unique stationary distribution $\pi_{F\hookrightarrow\mathcal{G}}$ that we defined in (3).*

(ii) *If we use Algorithm MP with* `AcceptProb` = `Approximate` *in Algorithm IM, then $(\mathbf{x}_t)_{t\geq 0}$ is an irreducible and aperiodic Markov chain with the unique stationary distribution $\hat{\pi}_{F\hookrightarrow\mathcal{G}}$ that is defined by*

$$\hat{\pi}_{F\hookrightarrow\mathcal{G}}(\mathbf{x}) = C' \hat{\pi}_{F\to\mathcal{G}}(\mathbf{x}) \, \mathbb{1}(\mathbf{x}(1), \ldots, \mathbf{x}(k) \text{ are distinct}) , \tag{17}$$

where $\hat{\pi}_{F\to\mathcal{G}}$ is the probability distribution on the set of homomorphisms $F \to \mathcal{G}$ in (12).

Proof. This assertion follows from standard Markov-chain theory (see, e.g., [61]) in conjunction with Propositions F.1 and F.2. □

F.2. **Convergence of the NDL algorithm.** Recall the problem statement for NDL in (7). Informally, we seek to learn r latent motifs $\mathcal{L}_1, \dots, \mathcal{L}_r \in \mathbb{R}^{k\times k}_{\geq 0}$ to minimize the expectation of the error of approximating the mesoscale patch $A_{\mathbf{x}}$ by a nonnegative combination of the motifs $\mathcal{L}_i$, where $\mathbf{x} : F \hookrightarrow \mathcal{G}$ is a random injective homomorphism that we sample from the distribution $\pi_{F\hookrightarrow\mathcal{G}}$ (3). We reformulate this problem as a matrix-factorization problem that generalizes (7). Let $\mathcal{C}^{\text{dict}}$ denote the set of all matrices $W \in \mathbb{R}^{k^2\times r}_{\geq 0}$ whose columns have a Frobenius norm of at most 1. The matrix-factorization problem is then

$$\operatorname*{arg\,min}_{W\in\mathcal{C}^{\text{dict}}\subseteq\mathbb{R}^{k^2\times r}_{\geq 0}} \left(f(W) := \mathbb{E}_{\mathbf{x}\sim\pi_{F\hookrightarrow\mathcal{G}}}\left[\ell(X^{(N)}(\mathbf{x}), W)\right]\right), \tag{18}$$

where we define the loss function

$$\ell(X, W) := \inf_{H\in\mathbb{R}^{r\times N}_{\geq 0}} \|X - WH\|_F^2 + \lambda\|H\|_1\,, \quad X \in \mathbb{R}^{k^2\times N}\,,\ W \in \mathbb{R}^{k^2\times r}\,. \tag{19}$$

The parameters $N \in \mathbb{N}$ and $\lambda \geq 0$ appear in Algorithm NDL. The former is the number of homomorphisms that we sample at each iteration of Algorithm NDL, and the latter is the coefficient of an L_1-regularizer that we use to find the code matrix H_t in (13). If $N = 1$ and $\lambda = 0$, then problem (18) is equivalent to problem (7) because $X^{(1)}(\mathbf{x})$ and the columns of W are vectorizations (using Algorithm A4) of the mesoscale patch $A_{\mathbf{x}}$ and the latent motifs $\mathcal{L}_1, \dots, \mathcal{L}_r$, respectively.

The objective function f in the optimization problem (18) for NDL is non-convex, so it is generally difficult to find a global optimum of f. However, local optima are often good enough for practical applications (such as in image restoration [22, 23]). We find that this is also the case for our network-denoising problem (see Figure 8). Theorems F.4 and F.7 guarantee that our NDL algorithm (see Algorithm NDL) finds a sequence $(W_t)_{t\geq 0}$ of dictionary matrices such that, almost surely, W_t is asymptotically a local optimum of the objective function f.

To make precise statements about asymptotic convergence of the NDL algorithm to a local optimum of the objective function in (7), we consider measures of the local optimality of non-convex constrained optimization problems. Suppose that we have a differentiable objective function $g : \mathbb{R}^p \to \mathbb{R}$ for some integer $p \geq 1$. Fix a parameter set $\mathbf{\Theta} \subseteq \mathbb{R}^p$. We say that $\mathbf{v}^* \in \mathbf{\Theta}$ is a *stationary point* of g in $\mathbf{\Theta}$ if

$$\inf_{\mathbf{v}\in\mathbf{\Theta}} \langle \nabla g(\mathbf{v}^*),\, \mathbf{v} - \mathbf{v}^*\rangle \geq 0\,,$$

where $\langle \cdot, \cdot\rangle$ denotes the dot project on $\mathbb{R}^p$. If $\mathbf{v}^*$ is a stationary point of g in $\mathbf{\Theta}$ and it is in the interior of $\mathbf{\Theta}$, then $\|\nabla g(\mathbf{v}^*)\| = 0$.

We are now ready to state the convergence result for our NDL algorithm (see Algorithm NDL) for non-bipartite networks.

Theorem F.4 (Convergence of the NDL Algorithm for Non-Bipartite Networks)**.** *Let $F = ([k], A_F)$ be a k-chain motif, and let $G = (V, A)$ be a network that satisfies the following properties:*

(a) The weight matrix A is bidirectional (i.e., $A(x,y) > 0$ implies that $A(y,x) > 0$ for all $x, y \in V$) and the binary network $(V, \mathbb{1}(A > 0))$ is connected and non-bipartite.

(b) For all $t \geq 0$, there exists a unique solution H_t in (13).

(c) For all $t \geq 0$, the eigenvalues of the positive semidefinite matrix A_t that is defined in (13) are at least as large as some constant $\kappa_1 > 0$.

Let $(W_t)_{t\geq 0}$ denote the sequence of dictionary matrices that we generate using Algorithm NDL. The following statements hold:

(i) *For* MCMC $\in$ {Pivot, Glauber}*, it is almost surely true as* $t \to \infty$ *that* W_t *converges to the set of stationary points of the objective function* f *that we defined in* (18)*. Furthermore, if* f *has finitely many stationary points in* $\mathcal{C}^{\mathrm{dict}}$*, it is then the case that* W_t *converges to a single stationary point of* f *almost surely as* $t \to \infty$*.*

(ii) *For* MCMC = PivotApprox*, it is almost surely true as* $t \to \infty$ *that* W_t *converges to the set of stationary points of the objective function*

$$\hat{f}(W) := \mathbb{E}_{\mathbf{x} \sim \hat{\pi}_{F \hookrightarrow \mathcal{G}}} \left[\ell(X^{(N)}(\mathbf{x}), W) \right] ,$$

where the distribution $\hat{\pi}_{F \hookrightarrow \mathcal{G}}$ *is defined in* (17) *and the loss function* ℓ *is defined in* (19)*. Furthermore, if* $\hat{f}$ *has finitely many stationary points in* $\mathcal{C}^{\mathrm{dict}}$*, it is then the case that* W_t *converges to a single stationary point of* $\hat{f}$ *almost surely as* $t \to \infty$*.*

Remark F.5. Assumptions (a)–(c) in Theorem F.4 are all reasonable and are easy to satisfy. Assumption (a) is satisfied if $\mathcal{G}$ is undirected, unweighted, and connected, which is the case for all of the networks that we study in the present paper. Assumptions (b) and (c) are standard assumptions in the study of online dictionary learning [13, 52, 73]. For instance, (b) is a common assumption in methods such as layer-wise adaptive-rate scaling (LARS) [62] that aim to find good solutions to problems of the form (19). See [52, Sec. 4.1] and [13, Sec. 4.1] for more detailed discussions of these assumptions.

Remark F.6. It is also possible to slightly modify both the optimization problem (18) and our NDL algorithm so that Theorem F.4 holds for the modified problem and the algorithm without needing to assume (b) and (c). The modified problem is

$$\underset{W \in \mathcal{C}^{\mathrm{dict}} \subseteq \mathbb{R}^{k^2 \times r}_{\geq 0}}{\arg\min} \left(\mathbb{E}_{\mathbf{x} \sim \pi} \left[\inf_{H \in \mathbb{R}^{r \times N}_{\geq 0}} \|X - WH\|_F^2 + \lambda \|H\|_1 + \kappa' \|H\|_F^2 + \lambda' \|W\|_F^2 \right] \right) , \tag{20}$$

where $\pi = \hat{\pi}_{F \to \mathcal{G}}$ if MCMC = PivotApprox and $\pi = \pi_{F \to \mathcal{G}}$ otherwise. The problem (20) is the same as problem (18) with additional quadratic penalization terms for both H and W in the loss function ℓ (see (19)). By contrast, consider the modification of the NDL algorithm (see Algorithm NDL) in which the objective function for H_t in Algorithm 13 has the additional term $\kappa' \|H\|_F^2$ and we replace P_t in Algorithm 13 by $P_t + \kappa' I$. When $\lambda' > 0$ and $\kappa' > 0$, the modified objective function for H_t is strictly convex, so H_t is unique. Therefore, H_t satisfies the uniqueness condition (b) in Theorem F.4. Additionally, the smallest eigenvalue of each matrix P_t that we compute using the modified NDL algorithm has a lower bound of κ_1 for all t, so it satisfies condition (c) in Theorem F.4. One can then show that all parts of Theorem F.4 for the modified problem (20) and the modified NDL algorithm hold without assumptions (b) and (c). The argument, which we omit, is almost identical to the proof of Theorem F.4.

Proof of Theorem F.4. The proof of the first part of **(i)** is almost identical to the proof of [13, Corollary 6.1]. Because Algorithm IM uses rejection sampling in addition to the pivot and the Glauber chains, we need to ensure that the following statements hold. First, we need the sequence of injective homomorphisms $(\mathbf{x}_t)_{t \geq 0}$ that we sample using Algorithm IM to be an irreducible and aperiodic Markov chain on the set of injective homomorphisms $F \hookrightarrow \mathcal{G}$. Second, we need $(\mathbf{x}_t)_{t \geq 0}$ to have a unique stationary distribution that coincides with $\pi_{F \hookrightarrow \mathcal{G}}$ (see (3)). We proved both of these statements in Proposition F.3.

To prove the first part of **(ii)**, we use the same essential argument as in the proof of [13, Corollary 6.1]. However, because our assertion is for the approximate pivot chain from the present article (see Algorithm MP with AcceptProb = Approximate), we need to use Proposition F.2 (instead of [21, Prop. 5.8]) to establish irreducibility and convergence of the associated

Markov chain. The proofs of the second parts of both **(i)** and **(ii)** are identical for exact and approximate pivot chains.

We give a detailed proof of **(ii)**. Let $\pi = \hat{\pi}_{F\hookrightarrow\mathcal{G}}$ if $\texttt{MCMC} = \texttt{PivotApprox}$ and $\pi = \pi_{F\hookrightarrow\mathcal{G}}$ for $\texttt{MCMC} \in \{\texttt{Pivot}, \texttt{Glauber}\}$ (see (2) and (12)). We define $(\mathbf{x}_t)_{t\geq 0}$, $(X_t)_{t\geq 1}$, and $(W_t)_{t\geq 0}$ as before. We use a general convergence result for online NMF for Markovian data [13, Theorem 4.1].

The matrices $X_t \in \mathbb{R}^{k^2\times N}_{\geq 0}$ that we compute in line 15 of Algorithm NDL do not necessarily form a Markov chain because the forward evolution of the Markov chain depends both on the induced mesoscale patches and on the actual homomorphisms $(\mathbf{x}_s)_{N(t-1)<s\leq Nt}$. However, if one considers the sequence $\overline{X}_t := (X_t, \mathbf{x}_{Nt})$, then $\overline{X}_t$ forms a Markov chain. Specifically, the distribution of X_{t+1} given X_t depends only on $\mathbf{x}_{Nt}$ and A. Indeed, $\mathbf{x}_{Nt}$ and A determine the distribution of the homomorphisms $(\mathbf{x}_s)_{Nt<s\leq N(t+1)}$, which in turn determine the $k^2 \times N$ matrix X_{t+1}.

With assumption (a), [21, Theorems 5.7 and 5.8] and Proposition F.3 imply that the Markov chain $(\mathbf{x}_t)_{t\geq 0}$ of injective homomorphisms $F \hookrightarrow \mathcal{G}$ is a finite-state Markov chain that is irreducible and aperiodic with a unique stationary distribution π (see (3)). This implies that the N-tuple of homomorphisms $(\mathbf{x}_s)_{N(t-1)<s\leq Nt}$ also yields a finite-state, irreducible, and aperiodic chain with a unique stationary distribution. Consequently, the Markov chain $(\overline{X}_t)_{t\geq 0}$ is also a finite-state, irreducible, and aperiodic chain with a unique stationary distribution. In this setting, one can regard Algorithm NDL as the online NMF algorithm in [13] for the input sequence $X_t = \varphi(\overline{X}_t)$, with $t \geq 1$, where $\varphi(X, \mathbf{x}) = X$ is the projection onto the first coordinate. Because $\overline{X}_t$ takes only finitely-many values, the range of φ is bounded. This verifies all hypotheses of [13, Theorem 4.1], so the first part of **(ii)** follows.

Now suppose that there are finitely many stationary points $W_1^*, \ldots, W_m^*$ of $\hat{f}$ in $\mathcal{C}^{\text{dict}}$. Because $\|W_{t-1} - W_t\|_F = O(1/t)$ (see [13, Prop. 7.5]), the first part of **(ii)** (which we proved above) implies that W_t converges to W_i^* almost surely for some unique index $i \in \{1, \ldots, m\}$. □

Our second convergence result for the NDL algorithm is similar to Theorem F.4, but it concerns the case in which the network $\mathcal{G}$ is bipartite. Suppose that $\mathcal{G}$ is bipartite and that F is a k-chain motif. Let $V_1 \cup V_2 = V$ be a bipartition of $\mathcal{G}$. Let Ω_0 denote the set of injective homomorphisms $F \hookrightarrow \mathcal{G}$. We can define a subset $\Omega_1 \subseteq \Omega_0$ of injective homomorphisms $F \hookrightarrow \mathcal{G}$ by $\mathbf{x} \in \Omega_1$ if and only if $\mathbf{x}(1) \in V_1$. Let $\Omega_2 = \Omega_0 \setminus \Omega_1$. Because $\mathcal{G}$ is bipartite, neither the pivot chain nor the Glauber chain is irreducible as Markov chains with the state space Ω. Consequently, the injective motif-sampling chains with the pivot chain or the Glauber chain (see Algorithm IM) are not irreducible on the state space Ω_0. However, they are irreducible when we restrict them to each Ω_i with a unique stationary distribution $\pi^{(i)}_{F\hookrightarrow\mathcal{G}}$. (See the proof of [21, Theorem 5.7].) More explicitly, we compute

$$\begin{aligned}\pi^{(i)}_{F\hookrightarrow\mathcal{G}}(\mathbf{x}) &:= \mathbb{P}_{\mathbf{y}\sim\pi_{F\hookrightarrow\mathcal{G}}}\left(\mathbf{y} = \mathbf{x} \,|\, \mathbf{y} \in \Omega_i\right) \\ &= \frac{1}{\mathtt{Z}_i}\left(\prod_{j\in\{2,\ldots,k\}} A(\mathbf{x}(j-1), \mathbf{x}(j))\right)\mathbb{1}(\mathbf{x} \text{ is injective}) = \frac{\mathtt{Z}_0}{\mathtt{Z}_i}\pi_{F\hookrightarrow\mathcal{G}}(\mathbf{x})\,, \quad i \in \{1, 2\}\,,\end{aligned} \tag{21}$$

where

$$\begin{aligned}\mathtt{Z}_i &= \sum_{\mathbf{x}\in\Omega_i}\prod_{j\in\{2,\ldots,k\}} A(\mathbf{x}(j-1), \mathbf{x}(j))\mathbb{1}(\mathbf{x} \text{ is injective}), \\ \mathtt{Z}_0 &= \sum_{\mathbf{x}\in\Omega_0}\prod_{j\in\{2,\ldots,k\}} A(\mathbf{x}(j-1), \mathbf{x}(j))\mathbb{1}(\mathbf{x} \text{ is injective}).\end{aligned}$$

We define two associated conditional expected loss functions

$$f^{(i)}(W) := \mathbb{E}_{\mathbf{x}\sim\pi^{(i)}_{F\hookrightarrow\mathcal{G}}}\left[\ell(X^{(N)}(\mathbf{x}), W)\right] = \mathbb{E}_{\mathbf{x}\sim\pi_{F\hookrightarrow\mathcal{G}}}\left[\ell(X^{(N)}(\mathbf{x}), W)\,\middle|\,\mathbf{x}\in\Omega_i\right], \quad i\in\{1,2\}, \tag{22}$$

where the equality follows from the first equality in (21). Because the Markov chain $(\mathbf{x}_t)_{t\geq 0}$ stays in Ω_i if it is initialized in Ω_i, the conditional expected loss functions $f^{(i)}$ are the natural objective function to minimize (instead of the expected loss function f in (18)). Similarly, for the approximate pivot chain, we define the distributions $\hat{\pi}^{(i)}_{F\hookrightarrow\mathcal{G}}$ and the conditional expected loss functions $\hat{f}^{(i)}$ as follows. For the probability distribution $\hat{\pi}_{F\hookrightarrow\mathcal{G}}$ (see (12)), let

$$\hat{\pi}^{(i)}_{F\hookrightarrow\mathcal{G}}(\mathbf{x}) := \mathbb{P}_{\mathbf{y}\sim\hat{\pi}_{F\hookrightarrow\mathcal{G}}}(\mathbf{y}=\mathbf{x}\,|\,\mathbf{y}\in\Omega_i) = \frac{1}{|V_i|}\left(\frac{\prod_{j\in\{2,\dots,k\}} A(\mathbf{x}(j-1),\mathbf{x}(j))}{\sum_{y_2,\dots,y_k\in V} A(\mathbf{x}(1),y_2)\prod_{i=3}^{k} A(y_{i-1},y_i)}\right) = \frac{|V|}{|V_1|}\hat{\pi}_{F\hookrightarrow\mathcal{G}}(\mathbf{x}),$$

$$f^{(i)}(W) := \mathbb{E}_{\mathbf{x}\sim\hat{\pi}^{(i)}_{F\hookrightarrow\mathcal{G}}}\left[\ell(X^{(N)}(\mathbf{x}), W)\right] = \mathbb{E}_{\mathbf{x}\sim\hat{\pi}_{F\hookrightarrow\mathcal{G}}}\left[\ell(X^{(N)}(\mathbf{x}), W)\,\middle|\,\mathbf{x}\in\Omega_i\right], \quad i\in\{1,2\}. \tag{23}$$

We now state our second convergence result for the NDL algorithm. This result is for bipartite networks. A convergence result that is analogous to the one in Theorem NDL holds for the associated conditional expected loss function. This implies that one can initialize homomorphisms in each Ω_i and compute sequences $W^{(i)}_t$ (with $i\in\{1,2\}$) of dictionary matrices to learn stationary points of both associated conditional expected loss functions $f^{(i)}$. However, when a k-chain motif $F=([k],A_F)$ has an even number of nodes, one only needs to compute one sequence of dictionary matrices, because one can obtain the other sequence by the algebraic operation of taking a 'mirror image' of a given square matrix. More precisely, define a map $\texttt{Flip}:\mathbb{R}^{k^2\times r}\to\mathbb{R}^{k^2\times r}$ that maps $W\mapsto\overline{W}$ with the jth column $\overline{W}(:,j)$ of $\overline{W}$ defined by

$$\bar{X}(:,j) := \texttt{vec}\circ\texttt{rev}\circ\texttt{reshape}(W(:,j)), \quad j\in\{1,\dots,r\},$$

where $W(:,j)$ denotes the jth column of W, the operator $\texttt{reshape}:\mathbb{R}^{k^2}\to\mathbb{R}^{k\times k}$ is the reshaping operator that we define in Algorithm A5, $\texttt{rev}$ maps a $k\times k$ matrix K to the $k\times k$ matrix $(\bar{K}_{ab})_{a,b\in\{1,\dots,k\}}$ with entries $\bar{K}_{ab}=K(k-a+1,k-b+1)$, and $\texttt{vec}$ denotes the vectorization operator in Algorithm A4. Applying $\texttt{Flip}$ twice gives the identity map.

Theorem F.7 (Convergence of the NDL Algorithm for Bipartite Networks)**.** *Let $F=([k],A_F)$ be a k-chain motif, and let $G=(V,A)$ be a network that satisfies the the following properties:*

(a') A is symmetric and the undirected and unweighted graph $(V,\mathbb{1}(A>0))$ is connected and bipartite.

(b) For all $t\geq 0$, there exists a unique solution H_t in (13).

(c) For all $t\geq 0$, the eigenvalues of the positive semidefinite matrix A_t in (13) are at least as large as some constant $\kappa_1>0$.

Let $(W_t)_{t\geq 0}$ denote the sequence of dictionary matrices that we generate using Algorithm NDL. The following statements hold:

(i) *Suppose that $\texttt{MCMC}\in\{\texttt{Pivot},\texttt{Glauber}\}$. For each $i\in\{1,2\}$, conditional on $\mathbf{x}_0\in\Omega_i$, the sequence of dictionary matrices W_t converges almost surely as $t\to\infty$ to the set of stationary points of the associated conditional expected loss function $f^{(i)}$ in (22). If $\texttt{MCMC}=$*

PivotApprox, *then the same statement holds with* $f^{(i)}$ *replaced by the function* $\hat{f}^{(i)}$ *in* (23). *If we also assume that* $f^{(i)}$ *(respectively,* $\hat{f}^{(i)}$*), with* $i \in \{1,2\}$*, has only finitely many stationary points in* $\mathcal{C}^{\text{dict}}$*, it then follows that* W_t *converges almost surely to a single stationary point of* $f^{(i)}$ *(respectively,* $\hat{f}^{(i)}$*) as* $t \to \infty$*.*

(ii) *Suppose that* MCMC = Glauber *in Algorithm* NDL *and that* k *is even. Assume that* $\mathbf{x}_0 \in \Omega_1$*. It then follows that, almost surely as* $t \to \infty$*, there is simultaneous convergence of* W_t *to the set of stationary points of* $f^{(1)}$ *and convergence of* $\overline{W}_t$ *to the set of stationary points of* $f^{(2)}$*. Moreover,* $f^{(1)}(W_t) = f^{(2)}(\overline{W_t})$ *for all* $t \geq 0$*. If we also assume that* $f^{(i)}$*, with* $i \in \{1,2\}$*, has only finitely many stationary points in* $\mathcal{C}^{\text{dict}}$*, it then follows that* W_t *converges to a stationary point of* $f^{(1)}$ *as* $t \to \infty$ *and* $\overline{W}_t$ *converges to a stationary point of* $f^{(2)}$ *as* $t \to \infty$*.*

Proof. We first prove **(i)**. Fix $i \in \{1,2\}$ and recall the conditional stationary distribution $\pi^{(i)}_{F\hookrightarrow\mathcal{G}}$ in (21). Conditional on $\mathbf{x}_0 \in \Omega_{i'}$, the Markov chain $(\mathbf{x}_t)_{t\geq 0}$ of injective homomorphisms is irreducible and aperiodic with a unique stationary distribution $\pi^{(i')}_{F\hookrightarrow\mathcal{G}}$. Recall that the conclusion of Theorem F.4 holds as long as the underlying Markov chain is irreducible. Therefore, W_t converges almost surely to the set of stationary points of the associated conditional expected loss function $f^{(i')}$ in (22). The same argument verifies the case with MCMC = PivotApprox.

We now verify **(ii)**. We first establish some notation and claims. Define $\mu_i := \pi^{(i)}_{F\hookrightarrow\mathcal{G}}$ and suppose that k is even. For each homomorphism $\mathbf{x} : F \to \mathcal{G}$, we define a map $\overline{\mathbf{x}} : [k] \to V$ by

$$\overline{\mathbf{x}}(j) := \mathbf{x}(k-j+1) \quad \text{for all} \quad j \in \{1,\dots,k\}\,.$$

Note that $\mathbf{x}$ is injective if and only if $\overline{\mathbf{x}}$ is injective. For even k, we have that $\mathbf{x} \in \Omega_1$ if and only if $\overline{\mathbf{x}} \in \Omega_2$. Because A is symmetric, it follows that

$$\prod_{j=1}^{k-1} A(\mathbf{x}(j),\mathbf{x}(j+1)) = \prod_{j=1}^{k-1} A(\mathbf{x}(j+1),\mathbf{x}(j)) = \prod_{j=1}^{k-1} A(\overline{\mathbf{x}}(j),\overline{\mathbf{x}}(j+1))\,.$$

Therefore, $\mathtt{Z}_1 = \mathtt{Z}_2 = \mathtt{Z}_0/2$. Consequently, for each $\mathbf{x} \in \Omega_1$, (21) implies that

$$\mu_1(\mathbf{x}) = \mu_2(\overline{\mathbf{x}}) = 2\pi_{F\hookrightarrow\mathcal{G}}(\mathbf{x})\,. \tag{24}$$

Consider two Glauber chains, $(\mathbf{z}_t)_{t\geq 0}$ and $(\mathbf{z}'_t)_{t\geq 0}$, where $\mathbf{z}_0 = \mathbf{y}$ and $\mathbf{z}'_0 = \overline{\mathbf{y}}$. We evolve these two Markov chains using a common source of randomness so that individually they have Glauber-chain trajectories; additionally, $\mathbf{z}'_t = \overline{\mathbf{z}_t}$ for all $t \geq 0$. We prove this by an induction on t. The claim clearly holds for $t = 0$. Suppose that $\mathbf{z}'_t = \overline{\mathbf{z}_t}$ for some $t \geq 0$. We want to show that $\mathbf{z}'_{t+1} = \overline{\mathbf{z}_{t+1}}$. For the update $\mathbf{z}_t \mapsto \mathbf{z}_{t+1}$ and $\mathbf{z}'_t \mapsto \mathbf{z}'_{t+1}$, we choose a node $v \in [k]$ uniformly at random and sample $z \in V$ according to the conditional distribution (11). We define

$$\begin{aligned} &\mathbf{z}_{t+1}(v) = z \quad \text{and} \quad \mathbf{z}_{t+1}(u) = \mathbf{z}_t(u) \quad \text{for} \quad u \neq v\,,\\ &\mathbf{z}'_{t+1}(k-v+1) = z \quad \text{and} \quad \mathbf{z}'_{t+1}(u) = \mathbf{z}_t(u) \quad \text{for} \quad u \neq k-v+1\,. \end{aligned}$$

The update $\mathbf{z}_t \mapsto \mathbf{z}_{t+1}$ follows the Glauber-chain update in Algorithm MG. Additionally, $\mathbf{z}'_{t+1} = \overline{\mathbf{z}}_{t+1}$ because

$$\begin{aligned} \mathbf{z}'_{t+1}(k-v+1) = z = \mathbf{z}_{t+1}(v) = \overline{\mathbf{z}}_{t+1}(k-v+1)\,,&\\ \mathbf{z}'_{t+1}(u) = \mathbf{z}'_t(u) = \overline{\mathbf{z}_t}(u) = \mathbf{z}_t(k-u+1) &= \mathbf{z}_{t+1}(k-u+1)\\ &= \overline{\mathbf{z}}_{t+1}(u) \quad \text{for} \quad u \neq k-v+1\,. \end{aligned}$$

Finally, we need to verify that $\mathbf{z}'_t \mapsto \mathbf{z}'_{t+1}$ also follows the Glauber-chain update in Algorithm MG. It suffices to check that $z \in V$ has the same distribution as $\mathbf{z}'_{t+1}(k-v+1)$. Because v is uniformly

distributed on $[k]$, so is $k-v+1$. The distribution of $z\in V$ is determined by

$$p(z)\propto\begin{cases}A(z,\mathbf{z}_t(2)))=A(z,\overline{\mathbf{z}_t}(k-1)))\,, & \text{if} \quad v=1\\ A(\mathbf{z}_t(v-1),z)A(z,\mathbf{z}_t(v+1)) & \\ \qquad\qquad =A(\overline{\mathbf{z}_t}(k-v),z)A(z,\overline{\mathbf{z}_t}(k-v+2))\,, & \text{if} \quad v\in\{2,\dots,k-1\}\\ A(\mathbf{z}_t(k-1),z))=A(\overline{\mathbf{z}_t}(2),z)\,, & \text{if} \quad v=k\,.\end{cases}$$

Because $\mathbf{z}_t'=\overline{\mathbf{z}_t}$, it follows that z follows the conditional distribution (11) of $\mathbf{z}_{t+1}'(k-v+1)$, as desired.

For the two Glauber chains, $\mathbf{z}_t$ and $\mathbf{z}_t'$, we observe that

$$\overline{X^{(N)}(\mathbf{y})}=X^{(N)}(\overline{\mathbf{y}}) \tag{25}$$

almost surely. This result follows from the facts that $\mathbf{z}_t'=\overline{\mathbf{z}_t}$ for all $t\geq 0$ and $\texttt{rev}(A_{\mathbf{z}})=A_{\overline{\mathbf{z}}}$ for all $\mathbf{z}\in\Omega$. (See (5) for the definition of $A_{\mathbf{z}}$.) From this, we note that

$$\begin{aligned} f^{(1)}(W)&=\mathbb{E}_{\mathbf{z}\sim\pi}\left[\ell(X^{(N)}(\mathbf{z}),W)\,\middle|\,\mathbf{z}\in\Omega_1\right] \qquad (26)\\ &=\mathbb{E}_{\mathbf{z}\sim\pi}\left[\ell\left(\overline{X^{(N)}(\mathbf{z})},\overline{W}\right)\,\middle|\,\overline{\mathbf{z}}\in\Omega_2\right]\\ &=\mathbb{E}_{\mathbf{z}\sim\pi}\left[\ell\left(X^{(N)}(\overline{\mathbf{z}}),\overline{W}\right)\,\middle|\,\overline{\mathbf{z}}\in\Omega_2\right]\\ &=\mathbb{E}_{\mathbf{z}\sim\pi}\left[\ell(X^{(N)}(\mathbf{z}),\overline{W})\,\middle|\,\mathbf{z}\in\Omega_2\right]=f^{(2)}(\overline{W})\,. \end{aligned}$$

The first and the last equalities use the second equality in (21). The second equality uses the fact that $\ell(X,W)=\ell(\overline{X},\overline{W})$. The third equality follows from (25). The fourth equality follows from the change of variables $\overline{\mathbf{z}}\mapsto\mathbf{z}$ and the fact that $\mathbf{z}\sim\pi$ if and only if $\overline{\mathbf{z}}\sim\pi$ (see (24))

We are now ready to prove **(ii)**. The first part of **(ii)** follows immediately from **(i)** and the above construction of Glauber chains $\mathbf{z}_t$ and $\mathbf{z}_t'$ that satisfy $\mathbf{z}_t'=\overline{\mathbf{z}_t}$ for all $t\geq 0$. Recall that the Markov chain of injective homomorphisms $\mathbf{x}_t$ is a subsequence of $\mathbf{z}_t$. Additionally, recall that $\mathbf{z}$ is injective if and only if $\overline{\mathbf{z}}$ is injective. Therefore, $\overline{\mathbf{x}}_t$ is the same subsequence of $\overline{z}$. That is, there exist integers t_l (with $l\geq 1$) such that $\mathbf{x}_l=\mathbf{z}_{t_l}$ and $\overline{\mathbf{x}}_l=\overline{\mathbf{z}_{t_l}}$. Let $W_t=W_t(\mathbf{x}_0)$ and $W_t'=W_t'(\mathbf{x}_0')$ denote the sequences of dictionary matrices that we compute using Algorithm NDL with initial (not necessarily injective) homomorphisms $\mathbf{x}_0$ and $\mathbf{x}_0'$, respectively. Suppose that $\mathbf{x}_0\in\Omega_1$, from which we see that $\mathbf{x}_0'=\overline{\mathbf{x}_0}\in\Omega_2$. By **(i)**, W_t and W_t' converge almost surely to the set of stationary points of the associated conditional expected loss functions $f^{(1)}$ and $f^{(2)}$, respectively. We complete the proof of the first part of **(ii)** by observing that, almost surely,

$$W_t'=\overline{W_t}\quad\text{for all}\quad t\geq 0\,. \tag{27}$$

The second part of **(ii)** follows immediately from (26).

We still need to verify (27). All $k\times k$ mesoscale patches $A_{\overline{\mathbf{x}_t}}=\texttt{ref}(A_{\mathbf{x}_t})$ have a reversed row and column ordering relative to their original ordering, so the $k\times k$ latent motifs that we train on such matrices also have this reversed ordering of rows and columns. One can check this claim by induction on t together with (25) and the uniqueness assumption (b). We omit the details. □

F.3. **Convergence and reconstruction guarantees of our NDR algorithm.** We prove various theoretical guarantees for our NDR algorithm (see Algorithm NDR) in Theorem F.10. Specifically, we show that the reconstructed network that we obtain using Algorithm NDR at iteration t converges almost surely to some limiting network as $t\to\infty$, and we give a closed-form

expression of the limiting network. We also derive an upper bound of the reconstruction error. Roughly, we state the bound as follows:

$$\text{Jaccard reconstruction error} \leq \frac{\text{mesoscale approximation error}}{2(k-1)},$$

where k denotes the number of nodes of a k-chain motif. The parameter k is effectively a scale parameter. In (34), we give a precise statement of the above bound.

Before stating our mathematical results, we first introduce some notation. Fix a network $\mathcal{G} = (V, A)$, a k-chain motif $F = ([k], A_F)$, and a homomorphism $\mathbf{x} : F \to \mathcal{G}$. In this discussion, we do not assume that a homomorphism $F \to \mathcal{G}$ is injective, as we use all sampled homomorphisms for our network reconstruction using our NDR algorithm (see Algorithm NDR), unlike in our NDL algorithm (see Algorithm NDL) for learning latent motifs. Let `denoising` denote the Boolean variable in Algorithm NDR. For each matrix $B : V^2 \to [0, \infty)$ and a node map $\mathbf{x} : [k] \to V$, define the $k \times k$ matrix $B_{\mathbf{x}}$ by

$$B_{\mathbf{x}}(a, b) := B(\mathbf{x}(a), \mathbf{x}(b)) \quad \text{for all} \quad a, b \in \{1, \dots, k\}.$$

If $B = A$, then $B_{\mathbf{x}} = A_{\mathbf{x}}$ is the same as the mesoscale patch of $\mathcal{G}$ that is induced by $\mathbf{x}$ (see (5)). Additionally, given a network $\mathcal{G} = (V, A)$, a k-chain motif $F = ([k], A_F)$, a homomorphism $\mathbf{x} : F \to \mathcal{G}$, and a nonnegative matrix $W \in \mathbb{R}^{k^2 \times r}_{\geq 0}$, let $\hat{A}_{\mathbf{x};W}$ denote the $k \times k$ matrix that we defined in line 16 of Algorithm NDR. This matrix depends on the Boolean variable `denoising`. Recall that $\hat{A}_{\mathbf{x};W}$ is a nonnegative linear approximation of $A_{\mathbf{x}}$ that uses W.

We consider the event $(x, y) \overset{\mathbf{x}}{\hookleftarrow} (a, b)$ using the following indicator function:

$$\begin{aligned} \mathbb{1}\left((x,y) \overset{\mathbf{x}}{\hookleftarrow} (a,b)\right) &:= \mathbb{1}(\mathbf{x}(a) = x,\, \mathbf{x}(b) = y) \\ &\quad \times \mathbb{1}\begin{pmatrix} \texttt{InjHom} = \texttt{F} \\ \text{or } \mathbf{x} \text{ is injective} \end{pmatrix} \mathbb{1}\begin{pmatrix} \texttt{denoising} = \texttt{F} \\ \text{or } A_F(a,b) = 0 \end{pmatrix}. \end{aligned} \tag{28}$$

For each homomorphism $\mathbf{x} : F \to \mathcal{G}$ and $x, y \in V$, we say that the pair (x, y) is *visited by* (a, b) *through* $\mathbf{x}$ whenever the indicator on the left-hand side of (28) is 1. Additionally,

$$N_{xy}(\mathbf{x}) := \sum_{a,b \in \{1,\dots,k\}} \mathbb{1}\left((x,y) \overset{\mathbf{x}}{\hookleftarrow} (a,b)\right) \tag{29}$$

is the total number of visits to (x, y) through $\mathbf{x}$. When $N_{xy}(\mathbf{x}) > 0$, we say that the pair (x, y) is *visited by* $\mathbf{x}$. In Algorithm NDR, observe that both $A_{\text{count}}(x, y)$ and $A_{\text{recons}}(x, y)$ change at iteration t if and only if $N_{xy}(\mathbf{x}_t) > 0$. Let

$$\Omega_{xy} := \left\{\mathbf{x} : F \to \mathcal{G} \,\middle|\, N_{xy}(\mathbf{x}) > 0\right\} \tag{30}$$

denote the set of all homomorphisms $\mathbf{x} : F \to \mathcal{G}$ that visit the pair (x, y).

Suppose that $\hat{G} = (V, \hat{A})$ is a reconstructed network for $\mathcal{G} = (V, A)$. Fix a probability distribution π on the set of homomorphisms $F \to \mathcal{G}$. For a matrix $Q : V^2 \to \mathbb{R}$, define the weighted L_1 norm

$$\|Q\|_{1,\pi} := \sum_{x,y \in V} |Q(x,y)|\, \mathbb{E}_{\mathbf{x} \sim \pi}[N_{xy}(\mathbf{x})].$$

We define the following two quantities:

$$\mathrm{JD}_\pi(\mathcal{G}, \hat{\mathcal{G}}) := \frac{\|A - \hat{A}\|_{1,\pi}}{\|A \vee \hat{A}\|_{1,\pi}}, \quad \mathrm{JD}(\mathcal{G}, \hat{\mathcal{G}}) := \frac{\|A - \hat{A}\|_1}{\|A \vee \hat{A}\|_1}, \tag{31}$$

where $A \vee \hat{A}$ is defined as $(A \vee \hat{A})(x,y) = A(x,y) \vee \hat{A}(x,y) = \max\{A(x,y), \hat{A}(x,y)\}$ for $x, y \in V$. We refer to $\mathrm{JD}_\pi(\mathcal{G}, \hat{\mathcal{G}})$ as the *Jaccard distance* between $\mathcal{G} = (V, A)$ and $\hat{G} = (V, \hat{A})$ with respect to π. We refer to $\mathrm{JD}(\mathcal{G}, \hat{\mathcal{G}})$ as the *unweighted Jaccard distance* between $\mathcal{G} = (V, A)$ and $\hat{G} = (V, \hat{A})$.

To make sense of the definitions in (31), consider the special case in which the weight matrices A and $\hat{A}$ are both symmetric and binary with 0 diagonal entries (i.e., no self-edges). We also assume that $k = 2$ and that π is the uniform distribution on the set of homomorphisms $F \to \mathcal{G}$. We then have that

$$\mathrm{JD}_\pi(\mathcal{G}, \hat{\mathcal{G}}) = \mathrm{JD}(\mathcal{G}, \hat{\mathcal{G}}) = 1 - \frac{\text{number of edges in both } \mathcal{G} \text{ and } \hat{\mathcal{G}}}{\text{number of edges in } \mathcal{G} \text{ or } \hat{\mathcal{G}}}\,. \tag{32}$$

The fraction in the right-hand side of (32) equals the Jaccard index between the edge sets of $\mathcal{G}$ and $\hat{\mathcal{G}}$. We used this Jaccard index as a reconstruction-accuracy measure in Figure 5 of the main manuscript. Consequently, it is reasonable to view the Jaccard distances $\mathrm{JD}_\pi(\mathcal{G}, \hat{\mathcal{G}})$ and $\mathrm{JD}(\mathcal{G}, \hat{\mathcal{G}})$, which coincide in this case, as the 'Jaccard reconstruction error' of reconstructing $\mathcal{G}$ as $\hat{\mathcal{G}}$.

In the following proposition, we show that, under mild conditions, the Jaccard distance $\mathrm{JD}_\pi(\mathcal{G}, \mathcal{G}')$ with respect to π is close to the unweighted Jaccard distance $\mathrm{JD}(\mathcal{G}, \mathcal{G}')$ if the weights $\mathbb{E}_{\mathbf{x}\sim\pi}[N_{xy}(\mathbf{x})]$ do not vary much for node pairs $(x, y) \in V^2$ with $|\Omega_{xy}| \geq 1$.

Proposition F.8. *Let $F = ([k], A_F)$ be a k-chain motif, and fix connected and symmetric networks $\mathcal{G} = (V, A)$ and $\hat{G} = (V, \hat{A})$ with the same node set V. Let π be a probability distribution on the set of homomorphisms $F \to \mathcal{G}$. Suppose that the following conditions hold:*

(a) If $x, y \in V$ satisfies $|\Omega_{xy}| \geq 1$, it follows that $\hat{A}(x, y) = 0$;

(b) Consider a homomorphism $\mathbf{x} : F \to \mathcal{G}$ with $\pi(\mathbf{x}) > 0$.

Let ρ denote the maximum value of the ratio $\mathbb{E}_{\mathbf{x}\sim\pi}[N_{xy}(\mathbf{x})]/\mathbb{E}_{\mathbf{x}\sim\pi}[N_{x'y'}(\mathbf{x})]$ for $x, x', y, y' \in V$ and suppose that $\mathbb{E}_{\mathbf{x}\sim\pi}[N_{x'y'}(\mathbf{x})] \neq 0$. We then have that

$$\rho^{-1}\mathrm{JD}(\mathcal{G}, \hat{\mathcal{G}}) \leq \mathrm{JD}_\pi(\mathcal{G}, \hat{\mathcal{G}}) \leq \rho\mathrm{JD}(\mathcal{G}, \hat{\mathcal{G}})\,.$$

Proof. Consider two nodes $x, y \in V$, and let $d_\mathcal{G}(x, y)$ denote the shortest-path distance between them in $\mathcal{G}$. This distance is the minimum number of edges in a walk on $\mathcal{G}$ that connects x and y. If $d_\mathcal{G}(x, y) > k$, we then have that $A(x, y) = \hat{A}(x, y) = 0$. Moreover, because $\mathcal{G}$ is symmetric, $d_\mathcal{G}(x, y) \leq k$ implies that there exists a homomorphism $\mathbf{x} : F \to \mathcal{G}$. By condition (b), it follows that $\mathbb{E}_{\mathbf{x}\sim\pi}\left[N_{xy}(\mathbf{x})\right] > 0$ if $d_\mathcal{G}(x, y) \leq k$. Let $c := \min_{x,y\in V,\, |\Omega_{pq}|\geq 1} \mathbb{E}_{\mathbf{x}\sim\pi}[N_{xy}(\mathbf{x})]$. We then have

$$\begin{aligned} c\,\|A - \hat{A}\|_1 &= \sum_{x,y\in V,\, d_\mathcal{G}(x,y)\leq k} \left|A(x,y) - \hat{A}(x,y)\right| c \\ &\leq \sum_{x,y\in V,\, d_\mathcal{G}(x,y)\leq k} \left|A(x,y) - \hat{A}(x,y)\right| \mathbb{E}_{\mathbf{x}\sim\pi}[N_{xy}(\mathbf{x})] \\ &= \sum_{x,y\in V} \left|A(x,y) - \hat{A}(x,y)\right| \mathbb{E}_{\mathbf{x}\sim\pi}[N_{xy}(\mathbf{x})] = \|A - \hat{A}\|_{1,\pi}\,. \end{aligned}$$

If we instead take $C := \max_{x,y\in V,\, |\Omega_{pq}|\geq 1} \mathbb{E}_{\mathbf{x}\sim\pi}[N_{xy}(\mathbf{x})]$, we obtain

$$\begin{aligned} C\|A - \hat{A}\|_1 &= \sum_{x,y\in V,\, d_\mathcal{G}(x,y)\leq k} \left|A(x,y) - \hat{A}(x,y)\right| C \\ &\geq \sum_{x,y\in V,\, d_\mathcal{G}(x,y)\leq k} \left|A(x,y) - \hat{A}(x,y)\right| \mathbb{E}_{\mathbf{x}\sim\pi}[N_{xy}(\mathbf{x})] = \|A - \hat{A}\|_{1,\pi}\,. \end{aligned}$$

Therefore, $C\|A-\hat{A}\|_1 \geq \|A-\hat{A}\|_{1,\pi}$. Moreover, by using a similar argument, we obtain $c\|A\vee\hat{A}\|_1 \leq \|A-\hat{A}\|_{1,\pi} \leq C\|A\vee\hat{A}\|_1$. We then let $\rho = C/c$ to complete the proof. □

Proposition F.9. *Let $G=(V,A)$ be a symmetric and connected network, and let $F=([k],A_F)$ be a k-chain motif. Suppose that $2(k-1) \leq \mathrm{diam}(\mathcal{G})$. For each node $x \in V$, it then follows that there exists an injective homomorphism $\mathbf{x}: F \hookrightarrow \mathcal{G}$ with $x \in \{\mathbf{x}(1),\dots,\mathbf{x}(k)\}$.*

Proof. Our proof proceeds by contradiction. Suppose that there is a node $x \in V$ for which there does not exist an injective homomorphism $\mathbf{x}: F \hookrightarrow \mathcal{G}$ with $x \in \{\mathbf{x}(1),\dots,\mathbf{x}(k)\}$. We will show that this implies that $\mathrm{diam}(\mathcal{G}) < 2(k-1)$, which contradicts the hypothesis.

Fix two nodes $y, y' \in V$. Consider a walk $(y_1,\dots,y_{\bar{a}})$ from x to y (with $y_1 = x$ and $y_{\bar{a}} = y$) and another walk $(z_1,\dots,z_{\bar{b}})$ from z to x (with $z_1 = y'$ and $z_{\bar{b}} = x$). We choose these walks so that the integers $\bar{a}, \bar{b} \geq 1$ are as small as possible. By minimality, we know that these walks are paths (i.e., all y_i are distinct and all z_i are distinct). If $\bar{a} \geq k$, then $(y_1,\dots,y_k)$ gives a k-path, so $\mathbf{x}_y : [k] \to V$ with $\mathbf{x}_y(i) = y_i$ for $i \in \{1,\dots,k\}$ is an injective homomorphism $F \hookrightarrow \mathcal{G}$ and $\mathbf{x}_y(1) = y_1 = x$. This contradicts our assumption about the node x that there is no injective homomorphism $\mathbf{x}: F \hookrightarrow \mathcal{G}$ with $x \in \{\mathbf{x}(1),\dots,\mathbf{x}(k)\}$. Therefore, $\bar{a} < k$. By a similar argument, we know that $\bar{b} < k$. Consequently, $(z_1,\dots,z_{\bar{b}}, y_1,\dots,y_{\bar{a}})$ is a $(\bar{b}+\bar{a})$-path from y' to y. This implies that $d_{\mathcal{G}}(z,y) \leq \bar{b}+\bar{a} < 2(k-1)$. Because the nodes y and y' are arbitrary, this implies that $\mathrm{diam}(\mathcal{G}) < 2(k-1)$, which contradicts the hypothesis. This completes the proof. □

We now state and prove our main theoretical result about our NDR algorithm (see Algorithm NDR).

Theorem F.10 (Guarantees of the NDR Algorithm (see Algorithm NDR) for Non-Bipartite Networks)**.** *Let $F=([k],A_F)$ be a k-chain motif, and fix a network $\mathcal{G}=(V,A)$ and a network dictionary $W \in \mathbb{R}^{k^2\times r}$. We use Algorithm NDR with inputs $\mathcal{G}$, F, and W and the parameter value $T=\infty$. Let $\hat{\mathcal{G}}_t = (V,\hat{A}_t)$ denote the network that we reconstruct at iteration t, and suppose that $\mathcal{G}$ satisfies assumption (a) of Theorem F.4. Let*

$$\pi := \begin{cases} \pi_{F\to\mathcal{G}} & \text{if } \mathtt{MCMC} \in \{\mathtt{Glauber},\mathtt{pivot}\} \text{ and } \mathtt{InjHom} = \mathtt{F} \\ \pi_{F\hookrightarrow\mathcal{G}} & \text{if } \mathtt{MCMC} \in \{\mathtt{Glauber},\mathtt{pivot}\} \text{ and } \mathtt{InjHom} = \mathtt{T} \\ \hat{\pi}_{F\to\mathcal{G}} & \text{if } \mathtt{MCMC} = \mathtt{PivotApprox} \text{ and } \mathtt{InjHom} = \mathtt{F} \\ \hat{\pi}_{F\hookrightarrow\mathcal{G}} & \text{if } \mathtt{MCMC} = \mathtt{PivotApprox} \text{ and } \mathtt{InjHom} = \mathtt{T}\,. \end{cases}$$

The following statements hold:

(i) *(Convergence of the network reconstruction) The network $\hat{\mathcal{G}}_t$ converges almost surely to some limiting network $\hat{\mathcal{G}}_\infty = (V,\hat{A}_\infty)$ in the sense that*

$$\lim_{t\to\infty} \hat{A}_t(x,y) = \hat{A}_\infty(x,y) \in [0,\infty) \quad \textit{almost surely for all } x,y \in V\,.$$

(ii) *(Limiting reconstructed network) Let $\hat{A}_\infty$ denote the limiting matrix in* **(i)***. For each $x,y \in V$, we then have that*

$$\hat{A}_\infty(x,y) = \sum_{\mathbf{y}\in\Omega_{xy}} \left[\sum_{a,b\in\{1,\dots,k\}} \hat{A}_{\mathbf{y};W}(a,b)\,\mathbb{1}\Big((x,y) \overset{\mathbf{y}}{\hookleftarrow} (a,b)\Big) \right] \frac{\pi(\mathbf{y})}{\mathbb{E}_{\mathbf{x}\sim\pi}\left[N_{xy}(\mathbf{x})\right]}\,, \tag{33}$$

where we regard the right-hand side to be 0 when $|\Omega_{xy}| = 0$.

(iii) *(Bounds for the Jaccard reconstruction error) Suppose that the range of A is contained in $\{0\}\cup[1,\infty)$ and assume that $\mathtt{denoising} = F$. Let $\hat{A}_\infty$ denote the limiting matrix in* **(i)***.*

We then have that

$$\rho^{-1}\mathrm{JD}(\mathcal{G},\hat{\mathcal{G}}) \le \mathrm{JD}_{\pi}(\mathcal{G},\hat{\mathcal{G}}) \le \frac{\mathbb{E}_{\mathbf{x}\sim\pi}\left[\|A_{\mathbf{x}} - \hat{A}_{\mathbf{x};W}\|_1\right]}{2(k-1)}\,, \tag{34}$$

where the constant $\rho > 0$ is as in Proposition F.8.

Proof. Let $\mathbf{x}$ denote a random homomorphism $F \to \mathcal{G}$ with distribution π, let $\mathbb{P}$ denote the associated probability measure, and let $\mathbb{E}$ denote the associated expectation.

We first verify **(i)** and **(ii)** simultaneously. Let $(\mathbf{x}_t)_{t\ge 0}$ denote the Markov chain that we generate during the reconstruction process (see Algorithm NDR). Each $\mathbf{x}_t$ is an injective homomorphism $F \hookrightarrow \mathcal{G}$ if $\texttt{InjHom} = \texttt{T}$, and each $\mathbf{x}_t$ is a homomorphism $F \to \mathcal{G}$ that may or may not be injective if $\texttt{InjHom} = \texttt{F}$. We fix $x, y \in V$ and let

$$M_t = \sum_{s=1}^{t} \sum_{a,b\in\{1,\dots,k\}} \mathbb{1}\left((x,y) \overset{\mathbf{x}_s}{\hookleftarrow} (a,b)\right) = \sum_{s=1}^{t} N_{xy}(\mathbf{x}_s)\,,$$

where we defined the indicator $\mathbb{1}\big((x,y) \overset{\mathbf{x}}{\hookleftarrow} (a,b)\big)$ in (28). If $M_t = 0$, then $\hat{A}_t(x,y) = 0$. Suppose that $M_t \ge 1$. The key observation is that

$$\begin{aligned}\hat{A}_t(x,y) &= \frac{1}{M_t}\sum_{s=1}^{t} \sum_{a,b\in\{1,\dots,k\}} \hat{A}_{\mathbf{x}_s;W}(a,b)\mathbb{1}\left((x,y) \overset{\mathbf{x}_s}{\hookleftarrow} (a,b)\right) \\ &= \sum_{a,b\in\{1,\dots,k\}} \frac{1}{M_t}\sum_{s=1}^{t} \sum_{\mathbf{y}\in\Omega_{xy}} \hat{A}_{\mathbf{y};W}(a,b)\mathbb{1}\left((x,y) \overset{\mathbf{x}_s}{\hookleftarrow} (a,b)\right)\mathbb{1}(\mathbf{x}_s = \mathbf{y}) \\ &= \sum_{\mathbf{y}\in\Omega_{xy}} \sum_{a,b\in\{1,\dots,k\}} \hat{A}_{\mathbf{y};W}(a,b)\mathbb{1}\left((x,y) \overset{\mathbf{y}}{\hookleftarrow} (a,b)\right) \frac{t}{M_t}\frac{1}{t}\sum_{s=1}^{t}\mathbb{1}(\mathbf{x}_s = \mathbf{y})\,.\end{aligned} \tag{35}$$

With assumption (a), the Markov chain $(\mathbf{x}_t)_{t\ge 0}$ of homomorphisms $F \to \mathcal{G}$ is irreducible and aperiodic with the unique stationary distribution π (see (2)). By the Markov-chain ergodic theorem (see, e.g., [71, Theorem 6.2.1 and Example 6.2.4] or [72, Theorem 17.1.7]), it follows that

$$\lim_{t\to\infty} \frac{t}{M_t}\frac{1}{t}\sum_{s=1}^{t}\mathbb{1}(\mathbf{x}_s = \mathbf{y}) = \frac{\mathbb{P}\left(\mathbf{x} = \mathbf{y}\right)}{\mathbb{E}\left[N_{xy}(\mathbf{x})\right]}\,.$$

By the definition of the probability distribution π, we have that $\pi(\mathbf{x}) > 0$ for all injective homomorphism $\mathbf{x} : F \hookrightarrow \mathcal{G}$. Therefore, $\mathbb{E}_{\mathbf{x}\sim\pi}[N_{xy}(\mathbf{x})] > 0$ if and only if $|\Omega_{xy}| \ge 1$. This proves both **(i)** and **(ii)**.

We now prove **(iii)**. Conditions (a) and (b) of Proposition F.8 are satisfied for $\hat{G} = \hat{G}_\infty$ with the assumed choice of π. Therefore, the first inequality in (34) follows immediately from Proposition F.8. To verify the second inequality, we prove a slightly more general result. Let $\mathcal{G}' = (V, B)$ be a network with the same node set V as $\mathcal{G} = (V, A)$. Assume that B is symmetric and that its range is contained in $\{0\} \cup [1, \infty)$. For each $a, b \in \{1, \dots, k\}$, define the indicator function

$$\mathbb{1}_{ab} := \mathbb{1}\begin{pmatrix}\texttt{denoising} = \texttt{F} \\ \text{or } A_F(a,b) = 0\end{pmatrix}\,. \tag{36}$$

We will show that

$$\frac{\|B-\hat{A}_\infty\|_{1,\pi}}{\|B\vee\hat{A}_\infty\|_{1,\pi}} \le \frac{1}{2(k-1)}\sum_{\mathbf{y}\in\Omega}\sum_{a,b\in\{1,\dots,k\}}\left|B_{\mathbf{y}}(a,b)\mathbb{1}_{ab}-\hat{A}_{\mathbf{y};W}(a,b)\mathbb{1}_{ab}\right|\pi(\mathbf{y})\,. \tag{37}$$

If $\texttt{denoising} = \texttt{F}$, the right-hand side of (37) reduces to $\frac{1}{2(k-1)}\mathbb{E}_{\mathbf{x}\sim\pi}[\|B_{\mathbf{x}}-\hat{A}_{\mathbf{x};W}\|_1]$, so **(iii)** is a special case of (37).

To verify (37), we first claim that

$$\|B-\hat{A}_\infty\|_{1,\pi} \le \sum_{\mathbf{y}\in\Omega}\sum_{a,b\in\{1,\dots,k\}}\left|B_{\mathbf{y}}(a,b)-\hat{A}_{\mathbf{y};W}(a,b)\right|\pi(\mathbf{y})\,. \tag{38}$$

For each $a,b\in\{1,\dots,k\}$ and $x,y\in\{1,\dots,n\}$, let $\Omega_{ab\to xy}$ denote the set of homomorphisms $\mathbf{x}:F\to\mathcal{G}$ such that $\mathbb{1}\big((x,y)\overset{\mathbf{x}}{\hookleftarrow}(a,b)\big)=1$. By changing the order of the sums, we rewrite the formula in (33) as

$$\hat{A}_\infty(x,y) = \sum_{a,b\in\{1,\dots,k\}}\sum_{\mathbf{y}\in\Omega_{ab\to xy}}\hat{A}_{\mathbf{y};W}(a,b)\frac{\mathbb{P}(\mathbf{x}=\mathbf{y})}{\mathbb{E}[N_{xy}(\mathbf{x})]}\,.$$

Additionally, observe that

$$\begin{aligned}\mathbb{E}[N_{xy}(\mathbf{x})] &= \mathbb{E}\left[\sum_{a,b\in\{1,\dots,k\}}\mathbb{1}(\mathbf{x}(a)=x,\,\mathbf{x}(b)=y)\,\mathbb{1}\begin{pmatrix}\texttt{InjHom}=\texttt{F}\\ \text{or } \mathbf{x} \text{ is injective}\end{pmatrix}\right]\\ &= \sum_{a,b\in\{1,\dots,k\}}\sum_{\mathbf{y}\in\Omega_{ab\to xy}}\mathbb{P}(\mathbf{x}=\mathbf{y})\,.\end{aligned}$$

We now calculate

$$\begin{aligned}&\sum_{x,y\in V}\left|B(x,y)-\hat{A}_\infty(x,y)\right|\mathbb{E}[N_{xy}(\mathbf{x})]\\ &\quad=\sum_{x,y\in V}\left|B(x,y)\,\mathbb{E}[N_{xy}(\mathbf{x})]-\sum_{a,b\in\{1,\dots,k\}}\sum_{\mathbf{y}\in\Omega_{ab\to xy}}\hat{A}_{\mathbf{y};W}(a,b)\,\mathbb{P}(\mathbf{x}=\mathbf{y})\right|\\ &\quad=\sum_{x,y\in V}\left|\sum_{a,b\in\{1,\dots,k\}}\sum_{\mathbf{y}\in\Omega_{ab\to xy}}\Big(B(x,y)-\hat{A}_{\mathbf{y};W}(a,b)\Big)\,\mathbb{P}(\mathbf{x}=\mathbf{y})\right|\\ &\quad\le\sum_{x,y\in V}\sum_{a,b\in\{1,\dots,k\}}\sum_{\mathbf{y}\in\Omega_{ab\to xy}}\left|B(\mathbf{y}(a),\mathbf{y}(b))-\hat{A}_{\mathbf{y};W}(a,b)\right|\mathbb{P}(\mathbf{x}=\mathbf{y})\\ &\quad=\sum_{x,y\in V}\sum_{a,b\in\{1,\dots,k\}}\sum_{\mathbf{y}\in\Omega}\left|B(\mathbf{y}(a),\mathbf{y}(b))-\hat{A}_{\mathbf{y};W}(a,b)\right|\\ &\qquad\qquad\times\,\mathbb{P}(\mathbf{x}=\mathbf{y})\,\mathbb{1}(\mathbf{y}(a)=x,\,\mathbf{y}(b)=y)\\ &\quad=\sum_{\mathbf{y}\in\Omega}\mathbb{P}(\mathbf{x}=\mathbf{y})\sum_{a,b\in\{1,\dots,k\}}\left|B_{\mathbf{y}}(a,b)-\hat{A}_{\mathbf{y};W}(a,b)\right|\sum_{x,y\in V}\mathbb{1}(\mathbf{y}(a)=x,\,\mathbf{y}(b)=y)\\ &\quad=\sum_{\mathbf{y}\in\Omega}\sum_{a,b\in\{1,\dots,k\}}\left|B_{\mathbf{y}}(a,b)-\hat{A}_{\mathbf{y};W}(a,b)\right|\pi(\mathbf{y})\,,\end{aligned}$$

where the indicator $\mathbb{1}_{ab}$ is defined in (36). This verifies the claim (38).

It now suffices to show that

$$\|B \vee \hat{A}_\infty\|_{1,\pi} \geq 2\|A_F\|_1 = 2(k-1)\,, \tag{39}$$

where the equality uses the fact that F is a k-chain motif. For each $a, b \in \{1, \dots, k\}$ and a homomorphism $\mathbf{x} : F \to \mathcal{G}$, observe that

$$(A_F(a,b) + A_F(b,a)) \sum_{x,y \in V} B(x,y) \sum_{\mathbf{y} \in \Omega_{ab\to xy}} \mathbb{1}(\mathbf{x} = \mathbf{y}) \geq A_F(a,b) + A_F(b,a)\,. \tag{40}$$

The inequality (40) uses the fact that $\mathbf{x}$ is a homomorphism. Therefore, $A_F(a,b) + A_F(b,a) > 0$ and $\{\mathbf{x}(a), \mathbf{x}(b)\} = \{x, y\}$ implies that $B(x,y) + B(y,x) > 0$. Because we assume that B is symmetric and that the range of B is contained in $\{0\} \cup [1, \infty)$, it follows that $B(x,y) \geq 1$ and thus verifies the inequality (40).

Recall the notation $\Omega_{ab\to xy}$ below the inequality (38), and observe that

$$\begin{aligned}
\|B \vee \hat{A}_\infty\|_{1,\pi} &= \sum_{x,y\in V} \left| B(x,y) \vee \hat{A}_\infty(x,y) \right| \mathbb{E}_{\mathbf{x}\sim\pi}\left[N_{xy}(\mathbf{x})\right] \\
&= \mathbb{E}_{\mathbf{x}\sim\pi}\left[\sum_{x,y\in V} \left| B(x,y) \vee \hat{A}_\infty(x,y) \right| N_{xy}(\mathbf{x}) \right] \\
&= \mathbb{E}_{\mathbf{x}\sim\pi}\left[\sum_{x,y\in V} \Big(B(x,y) \vee \hat{A}_\infty(x,y) \Big) \sum_{a,b\in\{1,\dots,k\}} \sum_{\mathbf{y}\in\Omega_{ab\to xy}} \mathbb{1}(\mathbf{x} = \mathbf{y}) \right] \\
&\geq \mathbb{E}_{\mathbf{x}\sim\pi}\left[\sum_{a,b\in\{1,\dots,k\}} \sum_{x,y\in V} B(x,y) \sum_{\mathbf{y}\in\Omega_{ab\to xy}} \mathbb{1}(\mathbf{x} = \mathbf{y}) \right] \\
&\geq \mathbb{E}_{\mathbf{x}\sim\pi}\left[\sum_{a,b\in\{1,\dots,k\}} (A_F(a,b) + A_F(b,a)) \sum_{x,y\in V} B(x,y) \sum_{\mathbf{y}\in\Omega_{ab\to xy}} \mathbb{1}(\mathbf{x} = \mathbf{y}) \right] \\
&= \mathbb{E}_{\mathbf{x}\sim\pi}\left[\sum_{a,b\in\{1,\dots,k\}} (A_F(a,b) + A_F(b,a)) \right] = 2\|A_F\|_1\,.
\end{aligned}$$

For the last inequality, we have used the fact that $(A_F(a,b) + A_F(b,a)) \in \{0, 1\}$. This proves (39), as desired. □

Remark F.11. Suppose that the original network $\mathcal{G} = (V, A)$ is binary. The bound on the Jaccard reconstruction error in Theorem F.10**(iii)** is for a direct comparison between the weighted reconstructed network $\hat{G} = (V, \hat{A})$ and the original binary network $\mathcal{G} = (V, A)$. In Figure 5 of the main manuscript, we instead used binary reconstructed networks $\hat{G}(\theta) := (V, \mathbb{1}(\hat{A} > \theta))$ that we obtain by thresholding the edge weights $\hat{A}(x,y)$ at some threshold $\theta \in (0,1)$. By modifying the argument in the proof of Theorem F.10**(iii)**, we obtain a similar bound for the Jaccard reconstruction error for the thresholded reconstructed network $\hat{G}(\theta) := (V, \mathbb{1}(\hat{A} > \theta))$. We now sketch the argument.

Let $\theta' := \min\{1-\theta,\, \theta\}$. For $\tilde{a} \in \{0,1\}$ and $\hat{a} \in [0,1]$, we obtain the inequalities

$$\theta' |\tilde{a} - \mathbb{1}(\hat{a} > \theta)| \leq |(\tilde{a} - \hat{a})\mathbb{1}(|\tilde{a} - \hat{a}| > \theta')| \leq |\tilde{a} - \hat{a}|\,.$$

Because $A : V^2 \to \{0,1\}$ and $\hat{A} : V^2 \to [0,1]$, it follows that

$$\theta' \|A - \mathbb{1}(\hat{A} > \theta)\|_{1,\pi} \leq \|(A - \hat{A})\mathbb{1}(|A - \hat{A}| > \theta')\|_{1,\pi} \leq \|A - \hat{A}\|_{1,\pi}\,.$$

By modifying the argument in the proof of Theorem F.10**(iii)**, one can show that

$$\theta' \mathrm{JD}_{\pi}(\mathcal{G}, \hat{\mathcal{G}}(\theta)) \leq \frac{\|(A-\hat{A})\mathbb{1}(|A-\hat{A}|>\theta')\|_1}{\|A\|_{1,\pi}} \overset{(*)}{\leq} \frac{\|A-\hat{A}\|_1}{\|A\|_{1,\pi}} \leq \frac{\mathbb{E}_{\mathbf{x}\sim\pi}\left[\|A_{\mathbf{x}} - \hat{A}_{\mathbf{x};W}\|_1\right]}{2(k-1)} . \quad (41)$$

By using Proposition F.8, one can also deduce that

$$\mathrm{JD}(\mathcal{G}, \hat{\mathcal{G}}(\theta)) \leq (\rho/\theta') \frac{\mathbb{E}_{\mathbf{x}\sim\pi}\left[\|A_{\mathbf{x}} - \hat{A}_{\mathbf{x};W}\|_1\right]}{2(k-1)} . \quad (42)$$

The inequality (42) gives a bound for the unweighted Jaccard distance between the original binary network $\mathcal{G}$ and the thresholded binary reconstructed network $\hat{\mathcal{G}}(\theta)$. However, the bound (42) is not sharp because of the possibly large constant ρ/θ' (which is at least 2.5 for $\theta = 0.4$). For instance, in Figure 7**b**, we see that if $\mathcal{G}$ is `UCLA` and W is the network dictionary of $r = 9$ latent motifs of `UCLA`, then the right-hand side of (42) is at least $2.5 \times 0.2 = 0.5$ for $\theta = 0.4$. However, in Figure 5**c**, we see the empirical value of the left-hand side of (42) is about 0.05. To obtain some insight into this discrepancy, observe that the second inequality in (41) (which we mark with $(*)$) becomes very crude if many entries of A and $\hat{A}$ do not differ by more than θ, which appears to be the case in our numerical computations.

Remark F.12. We discuss two implications of Theorem F.10**(iii)**.

The first implication is that we expect a network dictionary that tends to be effective at approximating the mesoscale patches of a network to also be effective at approximating the entire network. Suppose that `denoising` = `F` in Theorem F.10**(iii)**. Recall that the columns of W encode r latent motifs $\mathcal{L}_1, \ldots, \mathcal{L}_r \in \mathbb{R}^{k\times k}_{\geq 0}$ (see Appendix A.4). According to (34), we have a perfect reconstruction $\mathcal{G} = \hat{\mathcal{G}}_{\infty}$ if the right-hand side of (34) is 0. This is the case if $\sup_{\mathbf{x}:F\to\mathcal{G}} \ell(\texttt{vec}(A_{\mathbf{x}}), W) = 0$, which implies that W can perfectly approximate all mesoscale patches $A_{\mathbf{x}}$ of $\mathcal{G}$. However, the right-hand side of (34) can still be small if the worst-case approximation error $\sup_{\mathbf{x}:F\to\mathcal{G}} \ell(\texttt{vec}(A_{\mathbf{x}}), W)$ is large but the expected approximation error $\mathbb{E}_{\mathbf{x}\sim\pi}[\ell(\texttt{vec}(A_{\mathbf{x}}), W)]$ is small (i.e., when W is effective at approximating most of the mesoscale patches).

How can we find a network dictionary W that minimizes the right-hand side of (34)? Although it is difficult to find a globally optimal network dictionary W that minimizes the non-convex objective function on the right-hand side of (34), Theorems F.4 and F.7 guarantee that our NDL algorithm (see Algorithm NDL) always finds a locally optimal network dictionary. From these theorems, we know that the NDL algorithm with $N = 1$ computes a network dictionary W that is approximately a local optimum of the expected loss function

$$f(W) = \mathbb{E}_{\mathbf{x}\sim\pi}[\ell(\texttt{vec}(A_{\mathbf{x}}), W)] , \quad (43)$$

where $\pi = \hat{\pi}_{F\hookrightarrow\mathcal{G}}$ if `MCMC` = `PivotApprox` and $\pi = \pi_{F\hookrightarrow\mathcal{G}}$ if `MCMC` $\in$ {`Glauber`, `pivot`}. The function f in (43) is similar to the one in the upper bound in (34). In our experiments, we find that our NDL algorithm produces network dictionaries that are efficient at minimizing the reconstruction error. See Figure 5 and the left-hand sides of (34) and (42).

The second implication is that reconstructing a corrupted network using a network dictionary that is trained from an uncorrupted network will yield a network that is similar to the uncorrupted network. Consider an uncorrupted network $\mathcal{G}' = (V, B)$ and a corrupted network $\mathcal{G} = (V, A)$. Additionally, suppose that we have trained the network dictionary W for the uncorrupted network $\mathcal{G}'$, but that we use it to reconstruct the corrupted network $\mathcal{G}$. Even if $\hat{A}_{\mathbf{x};W}$ is a nonnegative linear approximation of the $k \times k$ matrix $A_{\mathbf{x}}$ of a mesoscale patch of the corrupted network $\mathcal{G}$, it may be close to the corresponding mesoscale patch $B_{\mathbf{x}}$ of the uncorrupted network

$\mathcal{G}'$ because we use the network dictionary W that we learned from the uncorrupted network $\mathcal{G}'$. Theorem F.10**(iii)** guarantees that the network $\hat{\mathcal{G}}_\infty$ that we reconstruct for the corrupted network $\mathcal{G}$ using the uncorrupted-network dictionary W is close to the uncorrupted network $\mathcal{G}'$.

Remark F.13. The update step (see line 17) for the reconstruction in Algorithm NDR indicates that we loop over all node pairs (a, b) in a k-chain motif and that we update the weight of the edge $\{\mathbf{x}_t(a), \mathbf{x}_t(b)\}$ in the reconstructed network using the homomorphism $\mathbf{x} : F \to \mathcal{G}$.

There may be multiple node pairs (a, b) in F that contribute to the edge $\{x, y\}$ in the reconstructed network because $\mathbf{x}_t(a) = x$ and $\mathbf{x}_t(b) = y$ can occur for multiple choices of (a, b). The output of this update step does not depend on the ordering of $a, b \in \{1, \dots, k\}$, as one can see from the expressions in (35).

One can also consider the following alternative update step for the reconstruction. In this alternative, we first choose two nodes, x and y, of the reconstructed network in the image $\{\mathbf{x}_t(j) \,|\, j \in \{1, \dots, k\}\}$ of the homomorphism $\mathbf{x}_t$ and average over all pairs $(a, b) \in \{1, \dots, k\}^2$ such that (x, y) is visited by (a, b) through $\mathbf{x}_t$. We then update the weight of (x, y) in the reconstructed network with this mean contribution from $\mathbf{x}_t$. Specifically, for each $a, b \in \{1, \dots, k\}$, let $\mathbb{1}\big((x, y) \overset{\mathbf{x}_t}{\hookleftarrow} (a, b)\big)$ denote the indicator in (28) and let $N_{xy}(\mathbf{x}_t) \geq 0$ denote the number of visits of $\mathbf{x}_t$ to (x, y) (see (29)). We can then replace lines 17–19 in Algorithm NDR with the following lines:

Alternative update for reconstruction:

For $x, y \in V$ such that $N_{xy}(\mathbf{x}_t) > 0$:

$$\widetilde{A}_{\mathbf{x}_t;W}(x, y) \leftarrow \frac{\sum_{1 \leq a,b \leq k} \hat{A}_{\mathbf{x}_t;W}(a, b) \mathbb{1}\big((x, y) \overset{\mathbf{x}}{\hookleftarrow} (a, b)\big)}{\sum_{1 \leq a,b \leq k} \mathbb{1}\big((x, y) \overset{\mathbf{x}}{\hookleftarrow} (a, b)\big)}\,, \qquad j \leftarrow A_{\text{count}}(x, y) + 1$$

$$A_{\text{recons}}(x, y) \leftarrow (1 - j^{-1}) A_{\text{recons}}(x, y) + j^{-1} \widetilde{A}_{\mathbf{x}_t;W}(x, y)\,.$$

For the alternative NDR algorithm that we just described, we can establish a convergence result that is similar to Theorem F.10 using a similar argument as the one in our proof of Theorem F.10. Specifically, **(i)** holds for the alternative NDR algorithm, so there exists a limiting reconstructed network. In **(ii)**, the formula for the limiting reconstructed network is now

$$\hat{A}_\infty(x, y) = \sum_{\mathbf{y} \in \Omega_{xy}} \widetilde{A}_{\mathbf{y};W}(x, y)\, \mathbb{P}_{\mathbf{x} \sim \pi}\left(\mathbf{x} = \mathbf{y} \,\middle|\, \mathbf{x} \in \Omega_{xy}\right) \quad \text{for all} \quad x, y \in V\,,$$

where Ω_{xy} is the set of all homomorphisms that visit (x, y) (see (30)). In particular, if $\mathcal{G}$ is an undirected and unweighted graph, then

$$\hat{A}_\infty(x, y) = \frac{1}{|\Omega_{xy}|} \sum_{\mathbf{y} \in \Omega_{xy}} \widetilde{A}_{\mathbf{y};W}(x, y) \quad \text{for all} \quad x, y \in V\,.$$

In the proof of **(iii)**, the same error bounds hold with $\mathbb{E}_{\mathbf{x} \sim \pi}[N_{xy}(\mathbf{x})]$ replaced by $\mathbb{P}_{\mathbf{x} \sim \pi}(\mathbf{x} \in \Omega_{xy})$. We omit the details of the proofs of the above statements for this alternative NDR algorithm.

We now discuss the convergence results of Algorithm NDR for a bipartite network $\mathcal{G}$. Recall our notation and our discussion of bipartite networks above Theorem F.7. Additionally, for bipartite networks, recall that there exist disjoint subsets Ω_1 and Ω_2 of the set Ω of all homomorphisms $F \to \mathcal{G}$ such that (1) $\Omega = \Omega_1 \cup \Omega_2$ and (2) the Markov chain $(\mathbf{x})_{t \geq 0}$ restricted to each Ω_i (with $i \in \{1, 2\}$) is irreducible but is not irreducible on the set Ω.

Theorem F.14 (Convergence of our NDR Algorithm (see Algorithm NDR) for Bipartite Networks)**.** *Let $F = ([k], A_F)$ be a k-chain motif, and let $\mathcal{G} = (V, A)$ be a network that satisfies*

assumption (a') in Theorem F.7. Let $\hat{\mathcal{G}}_t = (V, \hat{A}_t)$ denote the network that we reconstruct using Algorithm NDR at iteration t with a fixed network dictionary $W \in \mathbb{R}^{k^2 \times r}$. Fix $i \in \{1, 2\}$ and an initial (not necessarily injective) homomorphism $\mathbf{x}_0 \in \Omega_i$. Let $\pi = \hat{\pi}_{F\to\mathcal{G}}$ if $\texttt{MCMC} = \texttt{PivotApprox}$ *and $\pi = \pi_{F\to\mathcal{G}}$ for* $\texttt{MCMC} \in \{\texttt{Glauber}, \texttt{pivot}\}$*. The following properties hold:*

(i) *(Convergence of the network reconstruction) The network $\hat{\mathcal{G}}_t$ converges almost surely to some limiting network $\hat{\mathcal{G}}_\infty = (V, \hat{A}_\infty)$ in the sense that*

$$\lim_{t\to\infty} \hat{A}_t(x, y) = \hat{A}_\infty(x, y) \quad \text{almost surely for all } x, y \in V\,.$$

(ii)–(iii) *The same statements as in statements* **(ii)–(iii)** *of Theorem F.10 hold with the expectation $\mathbb{E}_{\mathbf{x}\sim\pi}$ replaced by the conditional expectation $\mathbb{E}_{\mathbf{x}\sim\pi}[\cdot \,|\, \mathbf{x} \in \Omega_i]$.*

(iv) *The results in* **(i)–(iii)** *do not depend on $i \in \{1, 2\}$ if k is even.*

Proof. The proofs of statements **(i)–(iii)** are identical to those for Theorem F.10. Statement **(iv)** follows from a similar argument as in the proof of Theorem F.7**(ii)** by constructing coupled Markov chains $(\mathbf{x}_t)_{t\geq 0}$ and $(\mathbf{x}'_t)_{t\geq 0}$ such that $\mathbf{x}'_t = \overline{\mathbf{x}_t}$ for all $t \geq 0$. □

Remark F.15. In Theorem F.14**(ii)**, let $\hat{G}^{(i)}_\infty = (V, \hat{A}^{(i)}_\infty)$, with $i \in \{1, 2\}$, denote the limiting reconstructed network for $\mathcal{G}$ conditional on initializing the Markov chain in the subset Ω_i. When k is even, Theorem F.14**(iv)** implies that $\hat{\mathcal{G}}^{(1)}_\infty = \hat{\mathcal{G}}^{(2)}_\infty$. When k is odd, we run the NDR algorithm (see Algorithm NDR) twice with the Markov chain initialized once in Ω_1 and once in Ω_2. We then define the network $\hat{\mathcal{G}}_\infty = (V, (\hat{A}^{(1)}_\infty + \hat{A}^{(2)}_\infty)/2)$ whose weight matrix is the mean of those of the two limiting reconstructed networks $\hat{\mathcal{G}}^{(i)}_\infty$ for $i \in \{1, 2\}$. We obtain a similar error bound as in Theorem F.14**(iii)** for the mean limiting reconstructed network $\hat{\mathcal{G}}_\infty$. In practice, one can obtain a sequence of reconstructed networks that converges to $\hat{\mathcal{G}}_\infty$ by reinitializing the Markov chain every τ iterations of the reconstruction procedure for any fixed τ.

Appendix G. Auxiliary Algorithms

We now present auxiliary algorithms for solving various subproblems of Algorithms NDL and NDR. Let Π_S denote the projection operator onto a subset S of a space. For each matrix A, let $[A]_{\bullet i}$ (respectively, $[A]_{i\bullet}$) denote the ith column (respectively, i^{th} row) of A.

Algorithm A1. Coding

Input: Data matrix $X \in \mathbb{R}^{d\times d'}$, dictionary matrix $W \in \mathbb{R}^{d\times r}$

Parameters: $T \in \mathbb{N}$ (the number of iterations), $\lambda > 0$ (the coefficient of an L_1-regularizer), $\mathcal{C}^{\text{code}} \subseteq \mathbb{R}^{r\times d'}$ (a convex constraint set of codes)

For $t = 1, \ldots, T$**:**

Do:

$$H \leftarrow \Pi_{\mathcal{C}^{\text{code}}}\left(H - \frac{1}{\text{tr}(W^T W)}(W^T W H - W^T X + \lambda \mathbf{1}_{d\times d'})\right),$$

where $\mathbf{1}_{d\times d'} \subseteq \mathbb{R}^{d\times d'}$ is the matrix with all 1 entries

Output: $H \in \mathcal{C}^{\text{code}} \subseteq \mathbb{R}^{r\times d'}$

Algorithm A2. Dictionary-Matrix Update

Input: Previous dictionary matrix $W_{t-1} \in \mathbb{R}^{k^2 \times r}$, previous aggregate matrices $(P_t, Q_t) \in \mathbb{R}^{r \times r} \times \mathbb{R}^{r \times N}$

Parameters: $\mathcal{C}^{\text{dict}} \subseteq \mathbb{R}^{k^2 \times r}$ (compactness and convexity constraint for dictionary matrices), $T \in \mathbb{N}$ (the number of iterations)

For $t = 1, \ldots, T$**:**

  $W \leftarrow W_{t-1}$

  For $j = 1, 2, \ldots, N$**:**

$$W(:, j) \leftarrow \Pi_{\mathcal{C}^{\text{dict}}} \left(W(:, j) - \frac{1}{A_t(j, j) + 1} (W P_t(:, j) - Q_t^T(:, j)) \right)$$

Output: $W_t = W \in \mathcal{C}^{\text{dict}} \subseteq \mathbb{R}_{\geq 0}^{k^2 \times r}$

Algorithm A3. Rejection Sampling of Homomorphisms

Input: Network $\mathcal{G} = (V, A)$, a k-chain motif $F = ([k], A_F)$

(▷ This algorithm works for all motifs, but we specialize it to k-chain motifs.)

Requirement: There exists at least one homomorphism $F \to \mathcal{G}$

Repeat: Sample a sequence $\mathbf{x} = [\mathbf{x}(1), \mathbf{x}(2), \ldots, \mathbf{x}(k)] \in V^{[k]}$ such that $\mathbf{x}(1), \ldots, \mathbf{x}(k)$ are independent and they each are sampled uniformly from V

  If $\prod_{i,j \in \{1,\ldots,k\}} A(\mathbf{x}(i), \mathbf{x}(j))^{A_F(i,j)} > 0$

    Return $\mathbf{x} : F \to \mathcal{G}$ and **Terminate**

Output: Homomorphism $\mathbf{x} : F \to \mathcal{G}$

Algorithm A4. Vectorization

Input: Matrix $X \in \mathbb{R}^{k_1 \times k_2}$

Output: Matrix $\texttt{vec}(X) := Y \in \mathbb{R}^{k_1 k_2 \times 1}$, where

$$Y(k_2(j-1) + i, 1) = X(i, j) \quad \text{for all } i \in \{1, \ldots, k_1\} \text{ and } j \in \{1, \ldots, k_2\}$$

Algorithm A5. Reshaping

Input: Matrix $X \in \mathbb{R}^{k_1 k_2 \times 1}$, a pair (k_1, k_2) of integers

Output: Matrix $\texttt{reshape}(X) := Y \in \mathbb{R}^{k_1 \times k_2}$, where

$$Y(i, j) = X(k_2(j-1) + i, 1) \quad \text{for all } i \in \{1, \ldots, k_1\} \text{ and } j \in \{1, \ldots, k_2\}$$

Appendix H. Additional Figures

In Supplementary Figures 5–7, we show additional binary-classification measures for the network-denoising experiments in Figure 8. In Supplementary Figures 8–15, we show latent motifs of all networks we consider in this work at various choices of parameters.

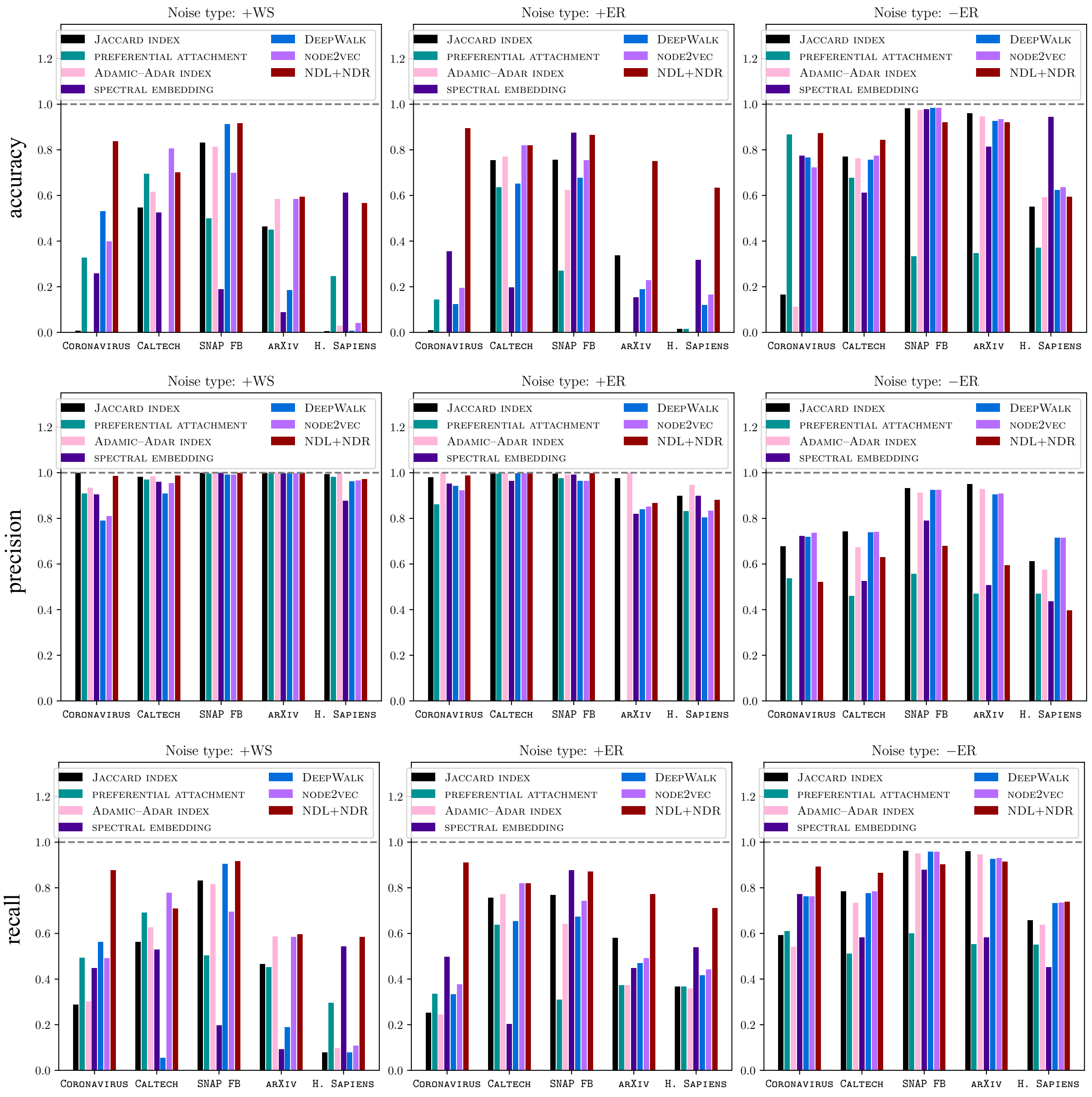

Supplementary Figure 5. The accuracy, precision, and recall scores for the network-denoising experiments in Figure 8 of the main manuscript. For convenience, we recall the definitions of these quantities, which one can use for binary classification. One summarizes the result of a binary classification using combinations of four quantities: TP (true positives), which is the number of positives that are classified as positive; TN (true negatives), which is the number of negatives that are classified as negative; FP (false positives), which is the number of positives that are classified as negative; and FN (false negatives), which is the number of negatives that are classified as positive. The total number of examples is the sum of these four quantities. Accuracy is $\frac{\text{TP}+\text{TN}}{\text{TP}+\text{TN}+\text{FP}+\text{FN}}$, precision is $\frac{\text{TP}}{\text{TP}+\text{FP}}$, and recall is $\frac{\text{TP}}{\text{TP}+\text{FN}}$.

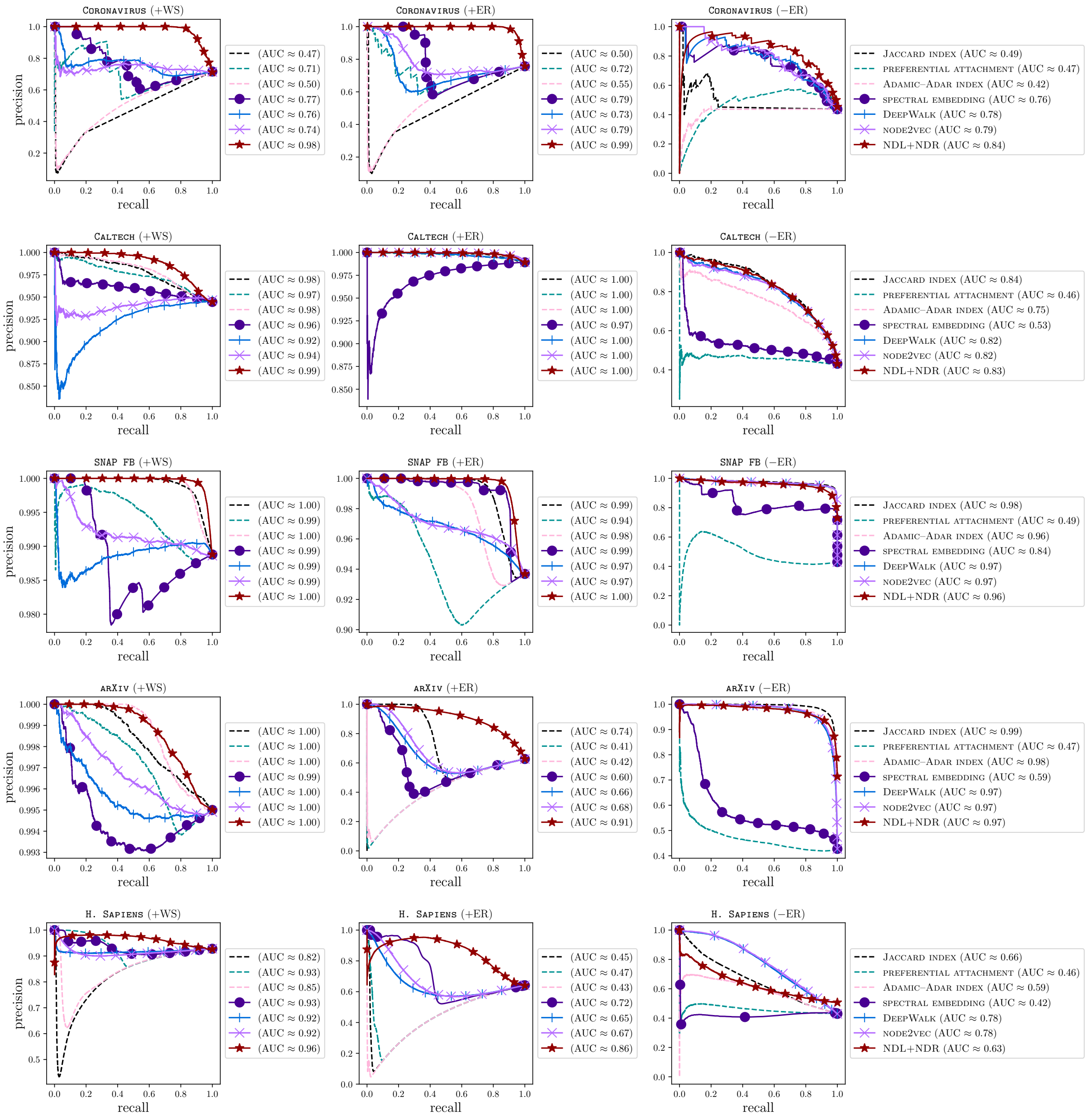

Supplementary Figure 6. The curves of precision versus recall for the network-denoising experiments in Figure 8 of the main manuscript. See the caption of Supplementary Figure 5 for the definition of precision and recall.

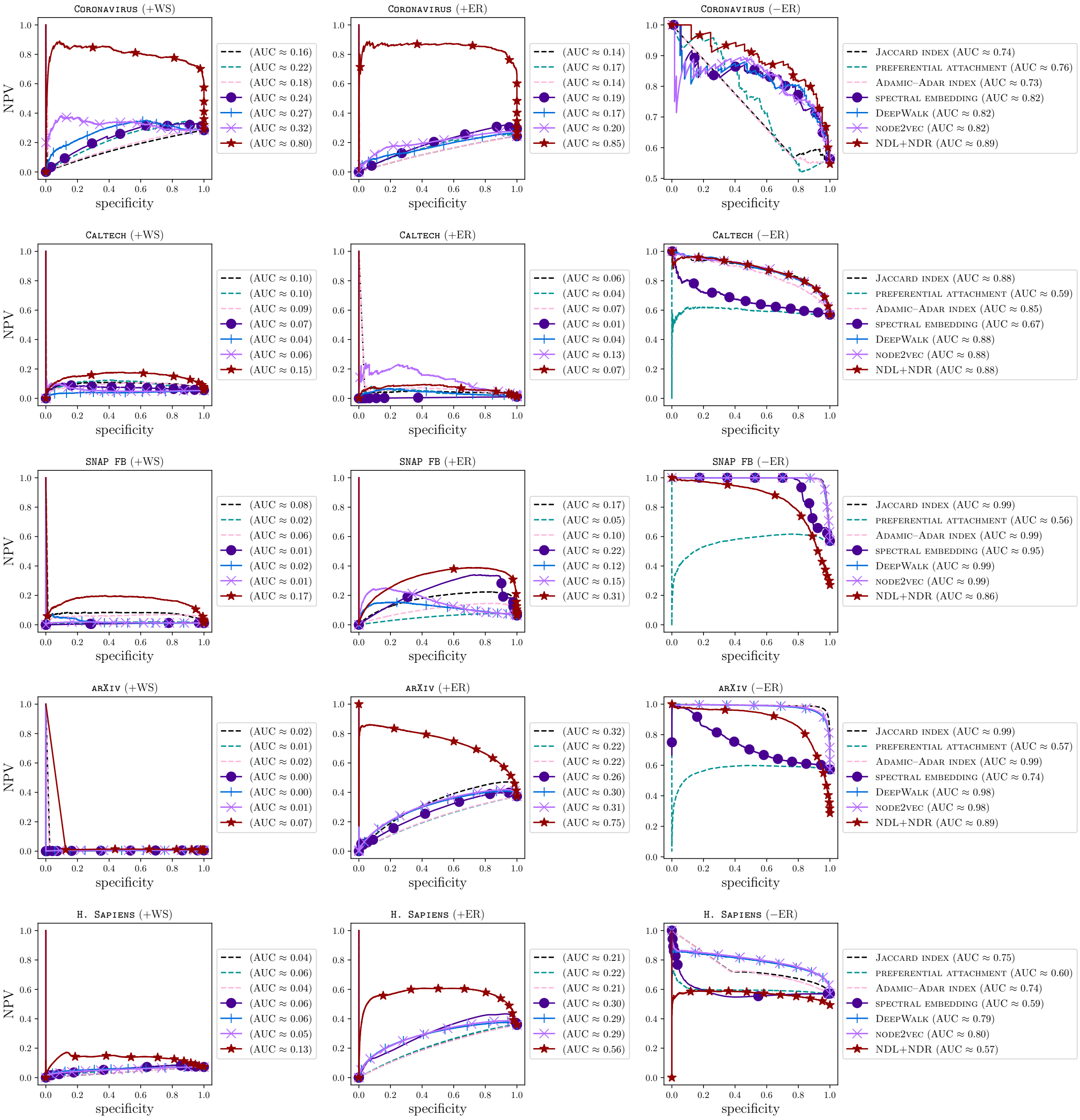

Supplementary Figure 7. The curves of negative predictive value (NPV) versus specificity for the network-denoising experiments in Figure 8 of the main manuscript. The NPV is $\frac{\mathrm{TN}}{\mathrm{TN}+\mathrm{FN}}$ and specificity is $\frac{\mathrm{TN}}{\mathrm{TN}+\mathrm{FP}}$. See [70] for a discussion of NPV and specificity.

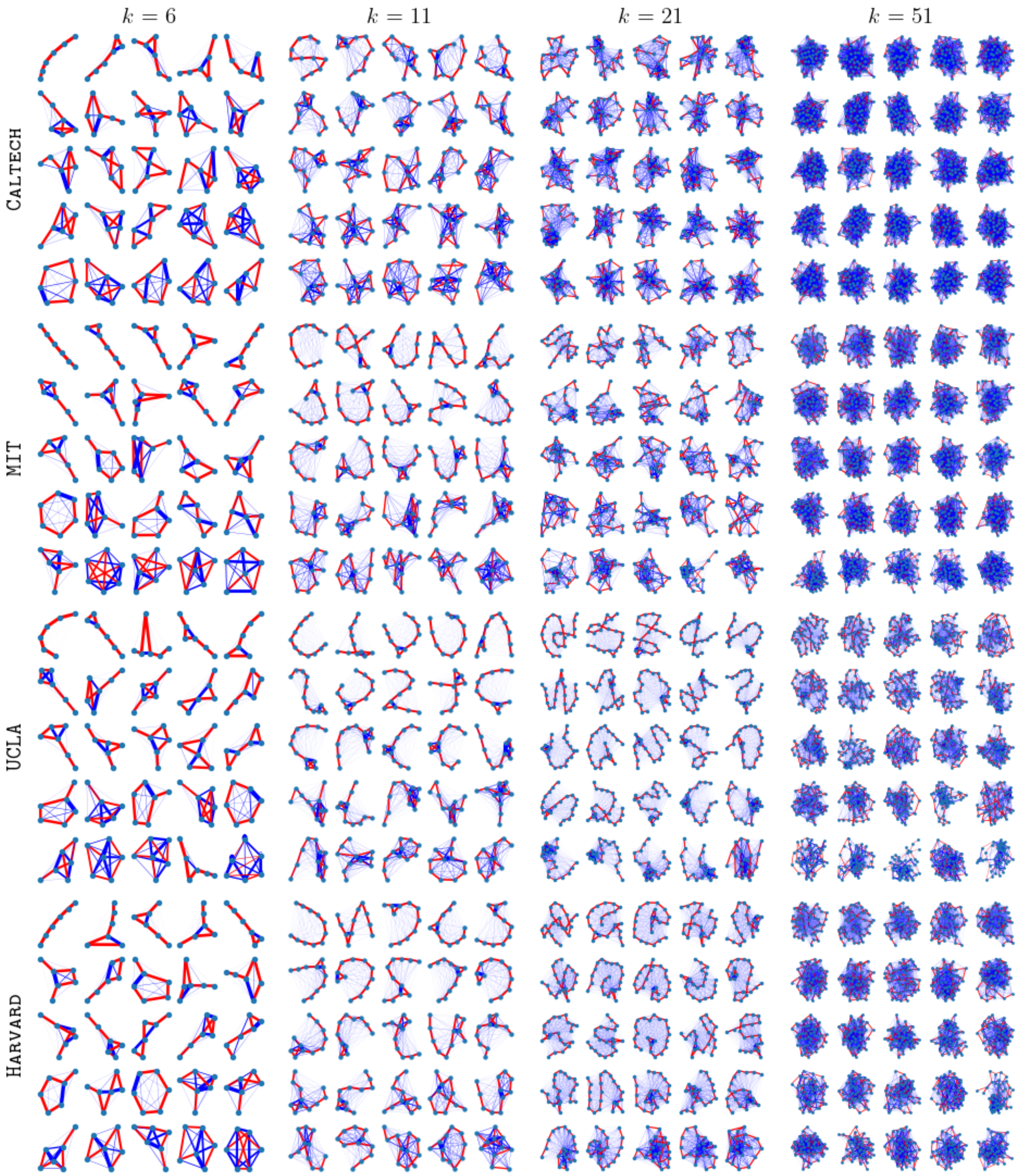

SUPPLEMENTARY FIGURE 8. The $r = 25$ latent motifs at scales $k = 6$, $k = 11$, $k = 21$, and $k = 51$ that we learn from the networks CALTECH, MIT, UCLA, and HARVARD. See Appendix E for the details of these experiments.

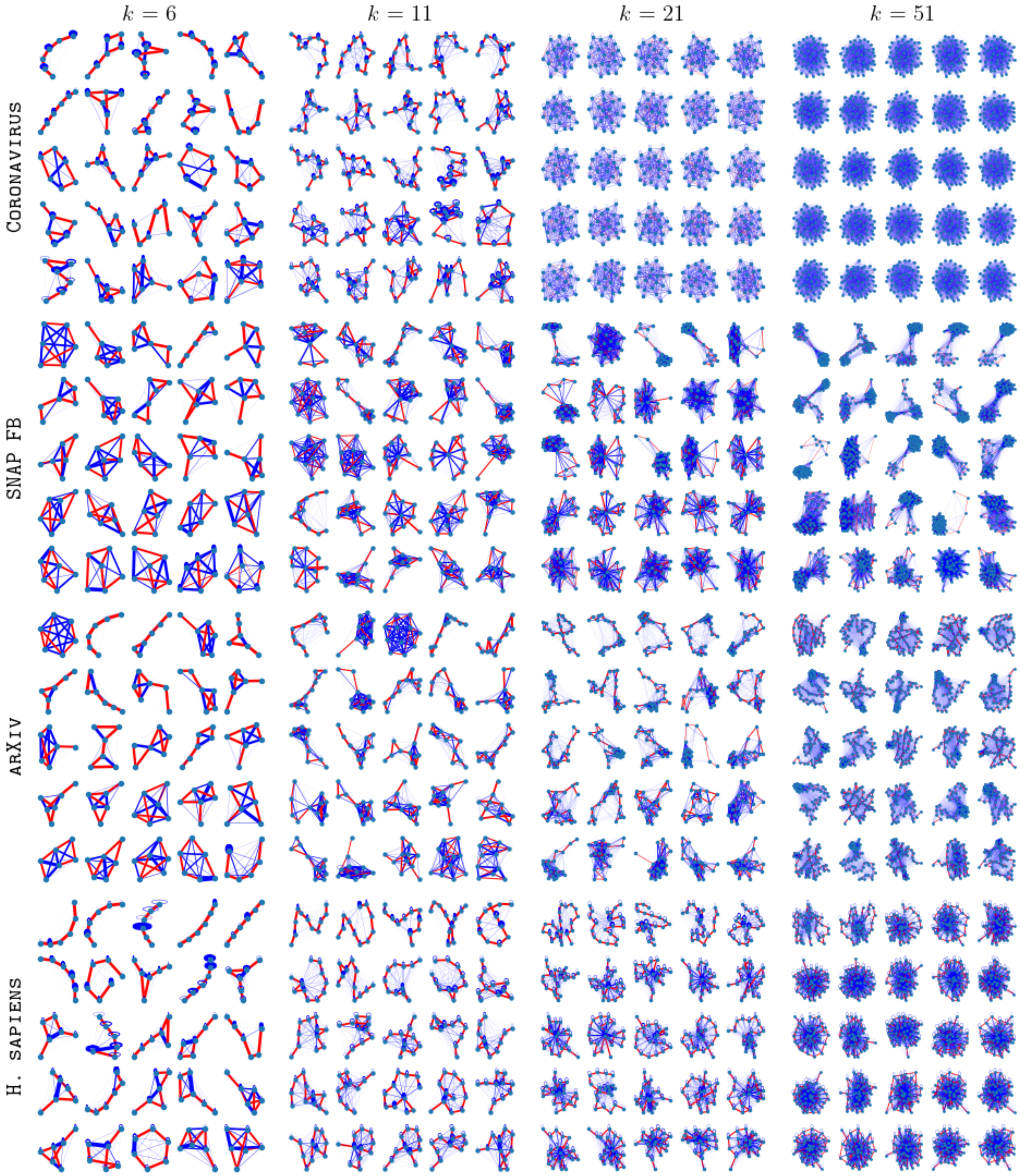

Supplementary Figure 9. The $r = 25$ latent motifs at scales $k = 6$, $k = 11$, $k = 21$, and $k = 51$ that we learn from the networks Coronavirus PPI, SNAP Facebook, arXiv ASTRO-PH, and Homo sapiens PPI. See Appendix E for the details of these experiments.

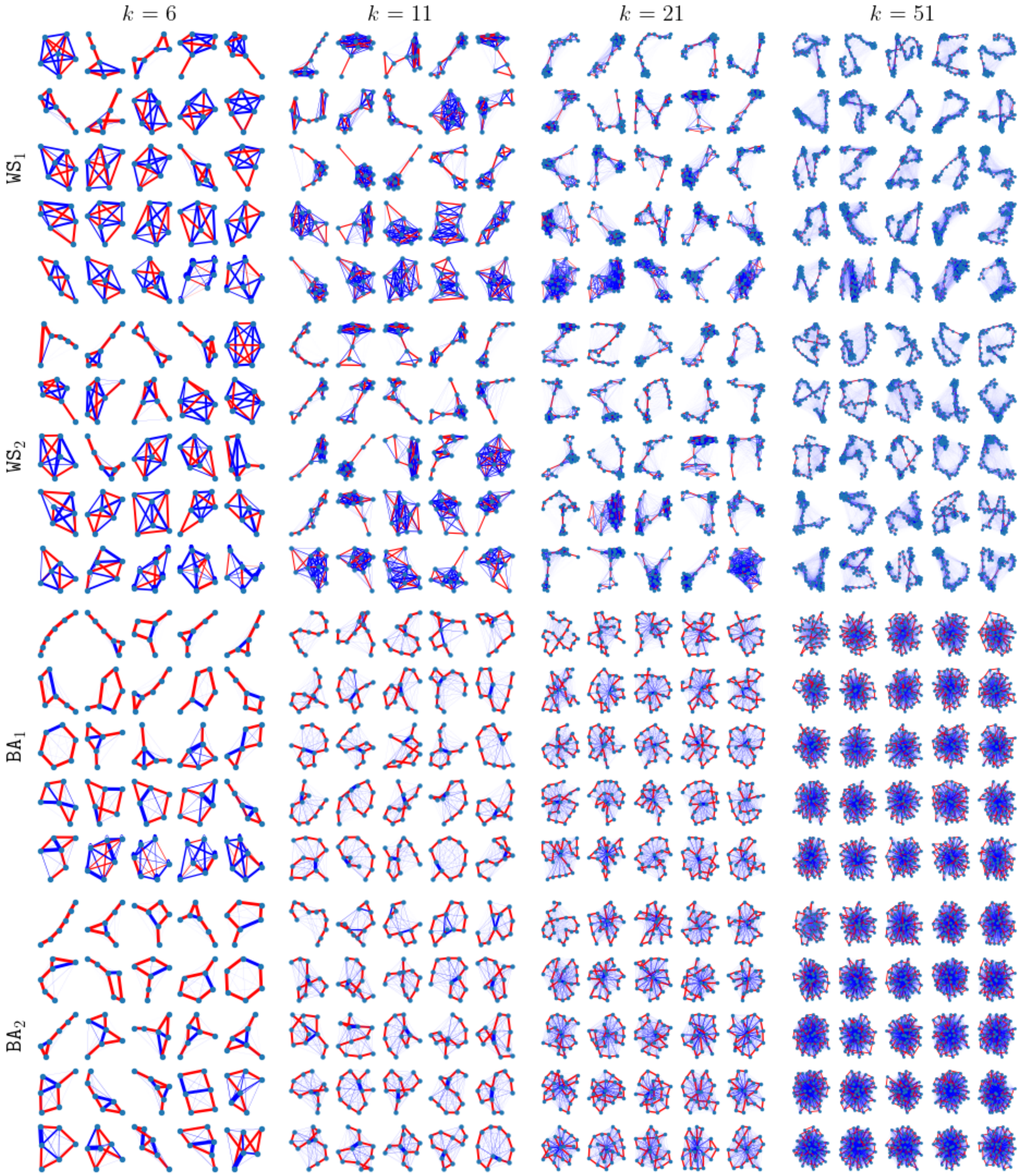

Supplementary Figure 10. The $r = 25$ latent motifs at scales $k = 6$, $k = 11$, $k = 21$, and $k = 51$ that we learn from the networks WS$_1$, WS$_2$, BA$_1$, and BA$_2$. See Appendix E for the details of these experiments.

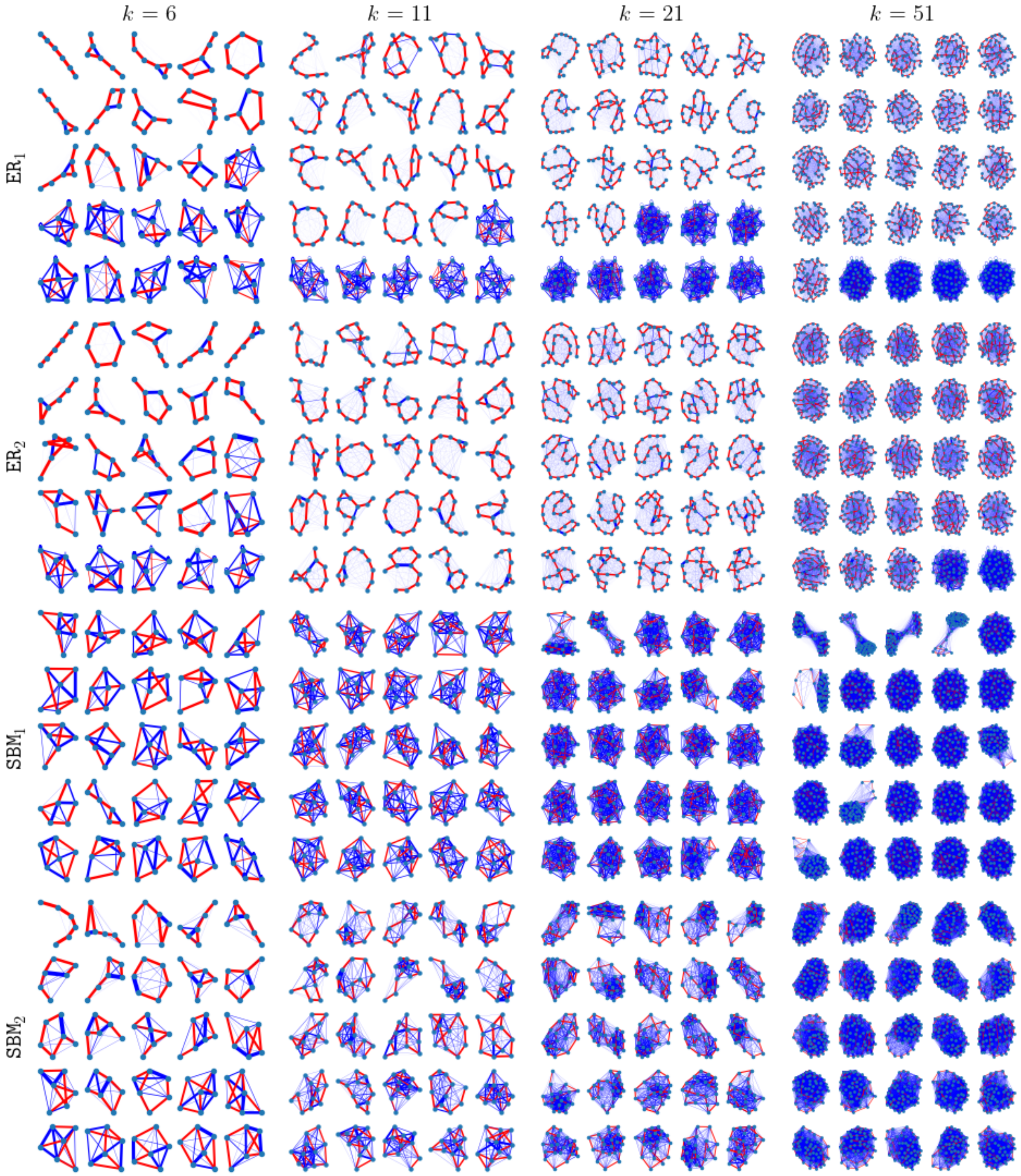

Supplementary Figure 11. The $r = 25$ latent motifs at scales $k = 6$, $k = 11$, $k = 21$, and $k = 51$ that we learn from the networks $\texttt{ER}_1$, $\texttt{ER}_2$, $\texttt{SBM}_1$, and $\texttt{SBM}_2$. See Appendix E for the details of these experiments.

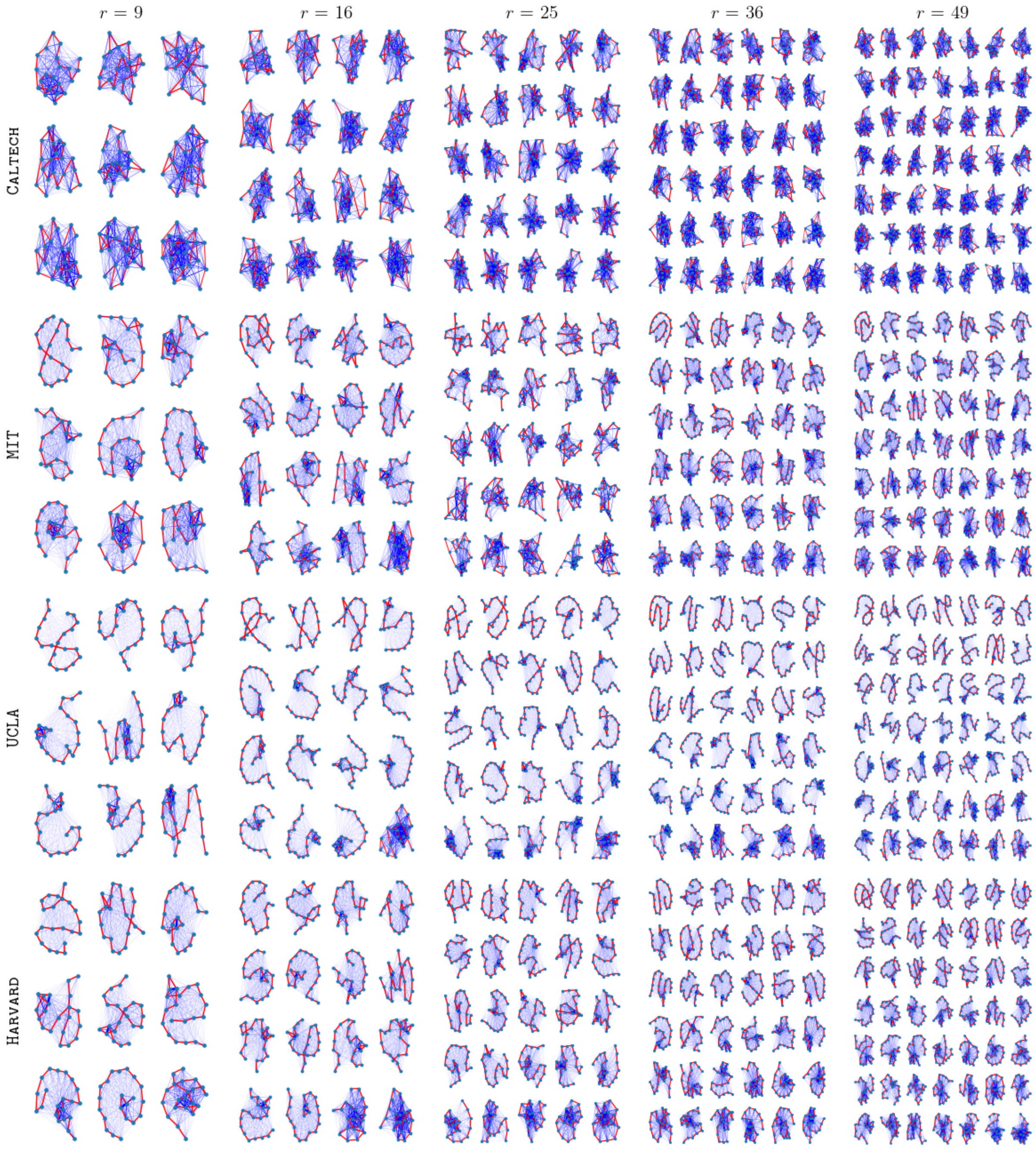

SUPPLEMENTARY FIGURE 12. The $r \in \{9, 16, 25, 36, 49\}$ latent motifs at scale $k = 21$ that we learn from the networks CALTECH, MIT, and UCLA. The $r = 25$ column is identical to the $k = 21$ column in Supplementary Figure 8. See Appendix E for the details of these experiments.

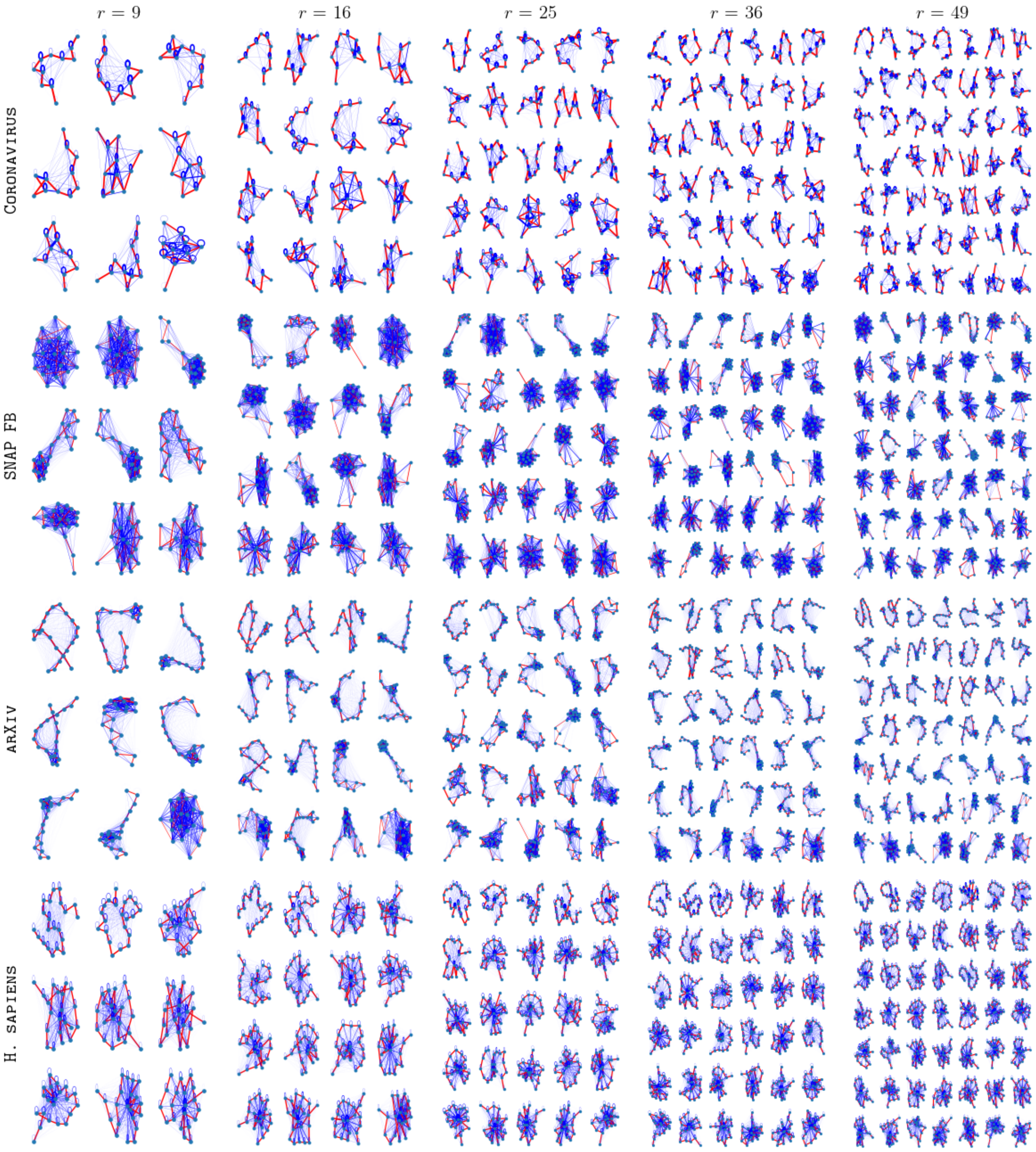

Supplementary Figure 13. The $r \in \{9, 16, 25, 36, 49\}$ latent motifs that we learn from the networks SNAP Facebook, arXiv ASTRO-PH, and Homo sapiens PPI at scale $k = 21$ and from Coronavirus PPI at scale $k = 11$. The $r = 25$ for column is identical to the $k = 21$ column in Supplementary Figure 9, except for Coronavirus PPI. See Appendix E for the details of these experiments.

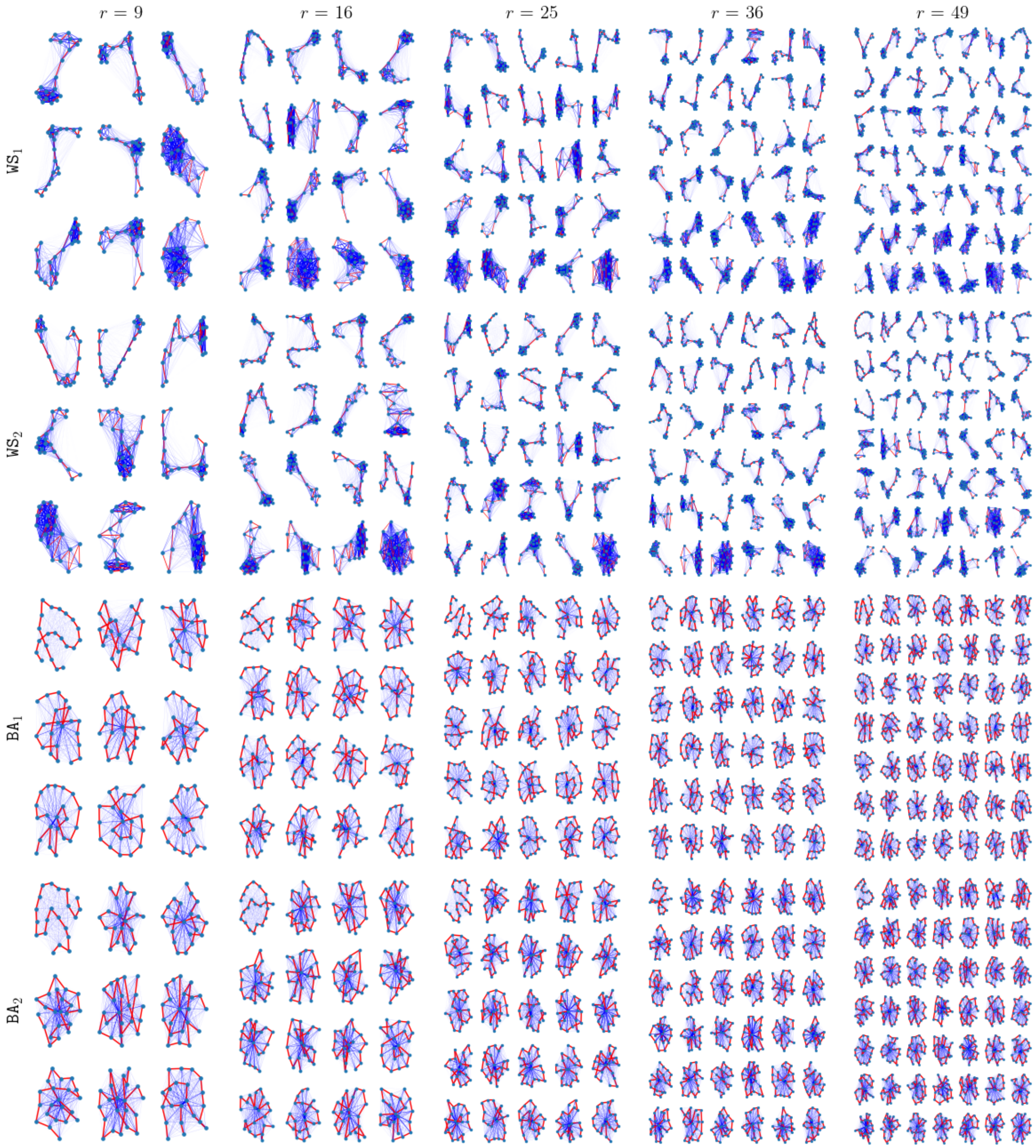

SUPPLEMENTARY FIGURE 14. The $r \in \{9, 16, 25, 36, 49\}$ latent motifs at scale $k = 21$ that we learn from the networks $\mathtt{WS}_1$, $\mathtt{WS}_2$, $\mathtt{BA}_1$, and $\mathtt{BA}_2$. The $r = 25$ column is identical to the $k = 21$ column in Supplementary Figure 10. See Appendix E for the details of these experiments.

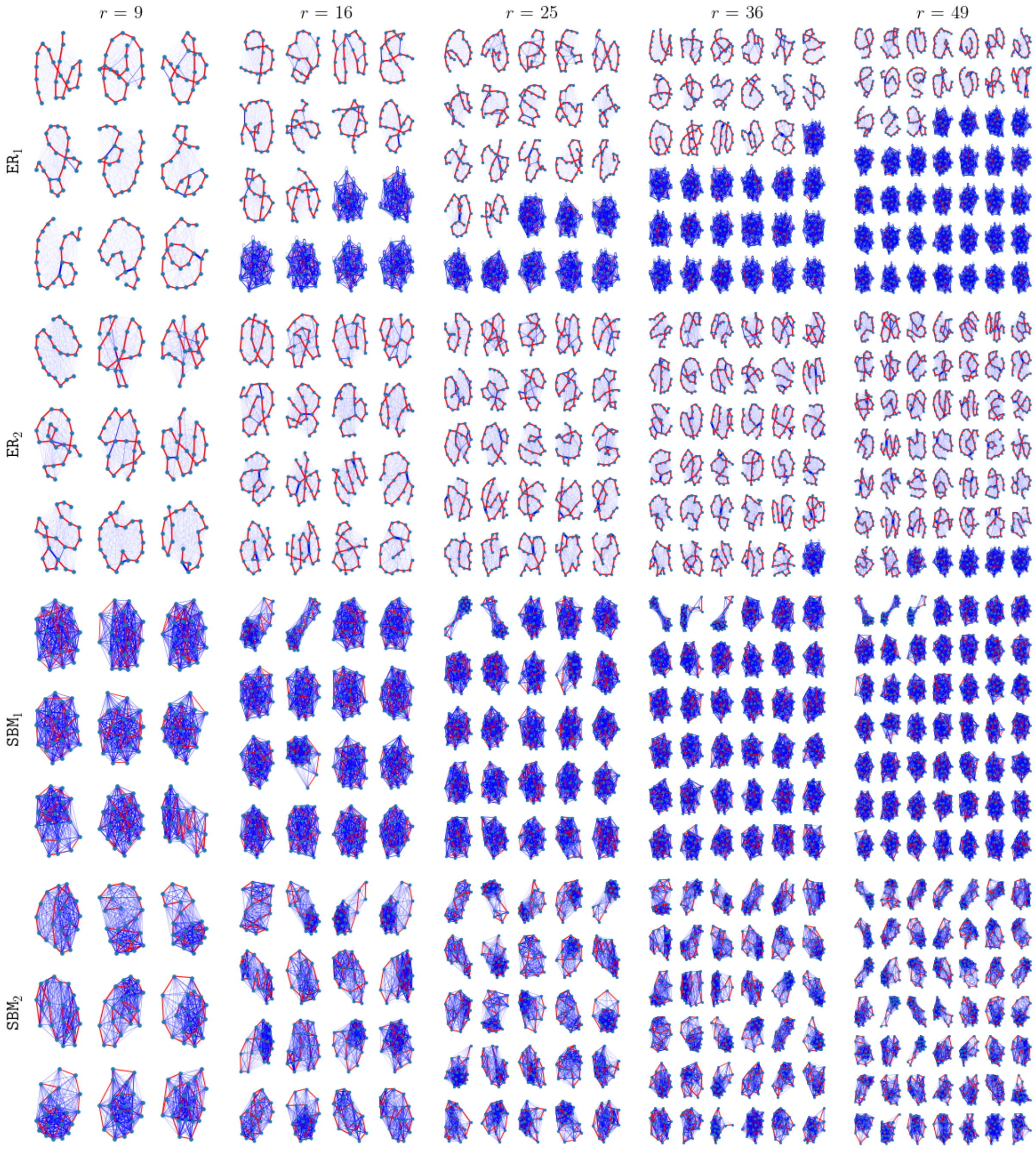

SUPPLEMENTARY FIGURE 15. The $r \in \{9, 16, 25, 36, 49\}$ latent motifs at scale $k = 21$ that we learn from the networks $\texttt{ER}_1$, $\texttt{ER}_2$, $\texttt{SBM}_1$, and $\texttt{SBM}_2$. The $r = 25$ column is identical to the $k = 21$ column in Supplementary Figure 10. See Appendix E for the details of these experiments.

Supplementary References

1. Newman, M. E. J. *Networks,* second edition (Oxford University Press, Oxford, UK, 2018).
2. Schwarze, A. C. & Porter, M. A. Motifs for processes on networks. *SIAM Journal on Applied Dynamical Systems* **20,** 2516–2557 (2021).
3. Milo, R. *et al.* Network motifs: Simple building blocks of complex networks. *Science* **298,** 824–827 (2002).
4. Conant, G. C. & Wagner, A. Convergent evolution of gene circuits. *Nature Genetics* **34,** 264–266 (2003).
5. Rip, J. M. K., McCann, K. S., Lynn, D. H. & Fawcett, S. An experimental test of a fundamental food web motif. *Proceedings of the Royal Society B: Biological Sciences* **277,** 1743–1749 (2010).
6. Sporns, O., Kötter, R. & Friston, K. J. Motifs in brain networks. *PLoS Biology* **2,** e369 (2004).
7. Ristl, K., Plitzko, S. J. & Drossel, B. Complex response of a food-web module to symmetric and asymmetric migration between several patches. *Journal of Theoretical Biology* **354,** 54–59 (2014).
8. Alon, U. Network motifs: Theory and experimental approaches. *Nature Reviews Genetics* **8,** 450–461 (2007).
9. Xu, H.-l., Yan, H.-b., Gao, C.-f. & Zhu, P. Social network analysis based on network motifs. *Journal of Applied Mathematics* **2014,** 874708 (2014).
10. Juszczyszyn, K., Kazienko, P. & Gabrys, B. Temporal changes in local topology of an email-based social network. *Computing and Informatics* **28,** 763–779 (2009).
11. Ohnishi, T., Takayasu, H. & Takayasu, M. Network motifs in an inter-firm network. *Journal of Economic Interaction and Coordination* **5,** 171–180 (2010).
12. Takes, F. W., Kosters, W. A., Witte, B. & Heemskerk, E. M. Multiplex network motifs as building blocks of corporate networks. *Applied Network Science* **3,** 39 (2018).
13. Lyu, H., Needell, D. & Balzano, L. Online matrix factorization for Markovian data and applications to network dictionary learning. *Journal of Machine Learning Research* **21,** 10148–10196 (2020).
14. Perozzi, B., Al-Rfou, R. & Skiena, S. *DeepWalk: Online learning of social representations* in *Proceedings of the 20th ACM SIGKDD International Conference on Knowledge Discovery and Data Mining* (2014), 701–710.
15. Grover, A. & Leskovec, J. *node2vec: Scalable feature learning for networks* in *Proceedings of the 22nd ACM SIGKDD International Conference on Knowledge Discovery and Data Mining* (2016), 855–864.
16. Seshadhri, C., Sharma, A., Stolman, A. & Goel, A. The impossibility of low-rank representations for triangle-rich complex networks. *Proceedings of the National Academy of Sciences of the United States of America* **117,** 5631–5637 (2020).
17. Akoglu, L., Tong, H. & Koutra, D. Graph based anomaly detection and description: A survey. *Data Mining and Knowledge Discovery* **29,** 626–688 (2015).
18. Noble, C. C. & Cook, D. J. *Graph-based anomaly detection* in *Proceedings of the Ninth ACM SIGKDD International Conference on Knowledge Discovery and Data Mining* (2003), 631–636.
19. Miller, B. A., Beard, M. S., Wolfe, P. J. & Bliss, N. T. A spectral framework for anomalous subgraph detection. *IEEE Transactions on Signal Processing* **63,** 4191–4206 (2015).
20. Ma, X. *et al.* A comprehensive survey on graph anomaly detection with deep learning. *IEEE Transactions on Knowledge and Data Engineering* **35,** 12012–12038 (2023).

21. Lyu, H., Mémoli, F. & Sivakoff, D. Sampling random graph homomorphisms and applications to network data analysis. *Journal of Machine Learning Research* **24,** 9 (pp. 1–79) (2023).
22. Elad, M. & Aharon, M. Image denoising via sparse and redundant representations over learned dictionaries. *IEEE Transactions on Image Processing* **15,** 3736–3745 (2006).
23. Mairal, J., Elad, M. & Sapiro, G. Sparse representation for color image restoration. *IEEE Transactions on Image Processing* **17,** 53–69 (2008).
24. Peyré, G. Sparse modeling of textures. *Journal of Mathematical Imaging and Vision* **34,** 17–31 (2009).
25. Lee, D. D. & Seung, H. S. Learning the parts of objects by non-negative matrix factorization. *Nature* **401,** 788–791 (1999).
26. Red, V., Kelsic, E. D., Mucha, P. J. & Porter, M. A. Comparing community structure to characteristics in online collegiate social networks. *SIAM Review* **53,** 526–543 (2011).
27. Traud, A. L., Mucha, P. J. & Porter, M. A. Social structure of Facebook networks. *Physica A* **391,** 4165–4180 (2012).
28. Porter, M. A., Onnela, J.-P. & Mucha, P. J. Communities in networks. *Notices of the American Mathematical Society* **56,** 1082–1097, 1164–1166 (2009).
29. Fortunato, S. & Hric, D. Community detection in networks: A user guide. *Physics Reports* **659,** 1–44 (2016).
30. Oughtred, R. *et al.* The BioGRID interaction database: 2019 update. *Nucleic Acids Research* **47,** D529–D541 (2019).
31. theBiogrid.org. Coronavirus PPI network. Retrieved from https://wiki.thebiogrid.org/doku.php/covid (downloaded 24 July 2020, Ver. 3.5.187.tab3) (2020).
32. Gordon, D. E. *et al.* A SARS-CoV-2 protein interaction map reveals targets for drug repurposing. *Nature* **583,** 459–468 (2020).
33. Leskovec, J. & McAuley, J. J. *Learning to discover social circles in ego networks* in *Proceedings of the 25th International Conference on Neural Information Processing Systems — Volume 1* (2012), 539–547.
34. Leskovec, J. & Krevl, A. SNAP Datasets: Stanford Large Network Dataset Collection. Retreived from http://snap.stanford.edu/data (downloaded 24 July 2020) (2020).
35. Erdős, P. & Rényi, A. On random graphs. I. *Publicationes Mathematicae* **6,** 290–297 (1959).
36. Watts, D. J. & Strogatz, S. H. Collective dynamics of 'small-world' networks. *Nature* **393,** 440–442 (1998).
37. Barabási, A.-L. & Albert, R. Emergence of scaling in random networks. *Science* **286,** 509–512 (1999).
38. Holland, P. W., Laskey, K. B. & Leinhardt, S. Stochastic blockmodels: First steps. *Social Networks* **5,** 109–137 (1983).
39. Rand, W. M. Objective criteria for the evaluation of clustering methods. *Journal of the American Statistical Association* **66,** 846–850 (1971).
40. Jeub, L. G. S., Balachandran, P., Porter, M. A., Mucha, P. J. & Mahoney, M. W. Think locally, act locally: Detection of small, medium-sized, and large large networks. *Physical Review E* **91,** 012821 (2015).
41. Correia, F. B., Coelho, E. D., Oliveira, J. L. & Arrais, J. P. Handling noise in protein interaction networks. *BioMed Research International* **2019,** 8984248 (2019).
42. Menon, A. K. & Elkan, C. *Link prediction via matrix factorization* in *Machine Learning and Knowledge Discovery in Databases* (eds Gunopulos, D., Hofmann, T., Malerba, D. & Vazirgiannis, M.) (Springer-Verlag, Heidelberg, Germany, 2011), 437–452.
43. Zhou, T. Progresses and challenges in link prediction. *iScience* **24,** 103217 (2021).

44. Liben-Nowell, D. & Kleinberg, J. The link-prediction problem for social networks. *Journal of the American Society for Information Science and Technology* **58,** 1019–1031 (2007).
45. Kovács, I. A. *et al.* Network-based prediction of protein interactions. *Nature Communications* **10,** 1240 (2019).
46. Guimerà, R. One model to rule them all in network science? *Proceedings of the National Academy of Sciences of the United States of America* **117,** 25195–25197 (2020).
47. Hasan, M. A. & Zaki, M. J. *A survey of link prediction in social networks* in *Social Network Data Analytics* (ed Aggarwal, C. C.) (Springer-Verlag, Heidelberg, Germany, 2011), 243–275.
48. Markovsky, I. & Usevich, K. *Low Rank Approximation* (Springer-Verlag, Heidelberg, Germany, 2012).
49. Paranjape, A., Benson, A. R. & Leskovec, J. *Motifs in temporal networks* in *Proceedings of the Tenth ACM International Conference on Web Search and Data Mining* (2017), 601–610.
50. Battiston, F., Nicosia, V., Chavez, M. & Latora, V. Multilayer motif analysis of brain networks. *Chaos: An Interdisciplinary Journal of Nonlinear Science* **27,** 047404 (2017).
51. Lee, D. D. & Seung, H. S. *Algorithms for non-negative matrix factorization* in *Proceedings of the 13th International Conference on Neural Information Processing Systems* (2001), 556–562.
52. Mairal, J., Bach, F., Ponce, J. & Sapiro, G. Online learning for matrix factorization and sparse coding. *Journal of Machine Learning Research* **11,** 19–60 (2010).
53. Mairal, J., Bach, F., Ponce, J., Sapiro, G. & Zisserman, A. *Non-local sparse models for image restoration* in (2009), 2272–2279.
54. theBiogrid.org. *Homo sapiens* PPI network. Retrieved from https://wiki.thebiogrid.org/doku.php/covid (downloaded 24 July 2020, Ver. 3.5.180.tab2) (2020).
55. Lovász, L. *Large Networks and Graph Limits* 475 (American Mathematical Society, Providence, RI, USA, 2012).
56. Bressan, M. *Efficient and near-optimal algorithms for sampling connected subgraphs* in *Proceedings of the 53rd Annual ACM SIGACT Symposium on Theory of Computing* (2021), 1132–1143.
57. Kashtan, N., Itzkovitz, S., Milo, R. & Alon, U. Efficient sampling algorithm for estimating subgraph concentrations and detecting network motifs. *Bioinformatics* **20,** 1746–1758 (2004).
58. Wernicke, S. Efficient detection of network motifs. *IEEE/ACM Transactions on Computational Biology and Bioinformatics* **3,** 347–359 (2006).
59. Leskovec, J. & Faloutsos, C. *Sampling from large graphs* in *Proceedings of the 12th ACM SIGKDD International Conference on Knowledge Discovery and Data Mining* (2006), 631–636.
60. Glasserman, P. *Monte Carlo Methods in Financial Engineering* (Springer-Verlag, Heidelberg, Germany, 2004).
61. Levin, D. A. & Peres, Y. *Markov Chains and Mixing Times* (American Mathematical Society, Providence, RI, USA, 2017).
62. Efron, B., Hastie, T., Johnstone, I. & Tibshirani, R. Least angle regression. *The Annals of Statistics* **32,** 407–499 (2004).
63. Tibshirani, R. Regression shrinkage and selection via the lasso. *Journal of the Royal Statistical Society: Series B (Methodological)* **58,** 267–288 (1996).
64. Lee, H., Battle, A., Raina, R. & Ng, A. Y. *Efficient sparse coding algorithms* in *Advances in Neural Information Processing Systems* (2007), 801–808.
65. Horn, R. A. & Johnson, C. R. *Matrix Analysis,* second edition (Cambridge University Press, Cambridge, UK, 2012).

66. Blondel, V. D., Guillaume, J.-L., Lambiotte, R. & Lefebvre, E. Fast unfolding of communities in large networks. *Journal of Statistical Mechanics: Theory and Experiment* **2008,** P10008 (2008).
67. Brown, G. W. & Mood, A. M. *On median tests for linear hypotheses* in *Proceedings of the Second Berkeley Symposium on Mathematical Statistics and Probability* **2** (Berkeley, CA, USA, 1951), 159–166.
68. Tang, L. & Liu, H. Leveraging social media networks for classification. *Data Mining and Knowledge Discovery* **23,** 447–478 (2011).
69. Mikolov, T., Sutskever, I., Chen, K., Corrado, G. S. & Dean, J. *Distributed representations of words and phrases and their compositionality* in *Proceedings of the 26th International Conference on Neural Information Processing Systems — Volume 2* (2013), 3111–3119.
70. Parikh, R., Mathai, A., Parikh, S., Sekhar, G. C. & Thomas, R. Understanding and using sensitivity, specificity and predictive values. *Indian Journal of Ophthalmology* **56,** 45 (2008).
71. Durrett, R. T. *Probability: Theory and Examples,* fourth edition (Cambridge University Press, Cambridge, UK, 2010).
72. Meyn, S. P. & Tweedie, R. L. *Markov Chains and Stochastic Stability* (Springer-Verlag, Heidelberg, Germany, 2012).
73. Mairal, J. *Stochastic majorization–minimization algorithms for large-scale optimization* in *Proceedings of the 26th International Conference on Neural Information Processing Systems — Volume 2* (2013), 2283–2291.
